# Supplementary material for: A push-pull strategy to control the western flower thrips, Frankliniella occidentalis, using alarm and aggregation pheromones
Source: PLoS One. 2023 Feb 24;18(2):e0279646. doi: 10.1371/journal.pone.0279646 (PMC9956899; doi:10.1371/journal.pone.0279646)
Supplement: S1 Fig — LA and LMB were synthesized from the precursor, lavandulol (1) in dichloromethane, which was reacted with acetyl chloride to yield LA, and was reacted with isovaleryl chloride to yield LMB, respectively, under the catalytic activity of trimethylamine. NMB was reacted with geraniol (‘4’) and isovaleryl chloride under a catalytic activity of trimethylamine. The purity of the compounds was analyzed by gas chromatography (8860 GC, Agilent, Santa Clara, CA, USA) with a DB-1 column (15 m × 0.350 mm, Agilent) at an oven temperature of 280°C and a flow rate of 1.0 mL/min. (DOCX) [file pone.0279646.s001.docx]

**S1 Fig.** Chemical synthesis of the aggregation pheromone components of thrips: lavandulyl acetate (LA, 2), lavandulyl methylbutanoate (LMB, 3), and neryl methylbutanoate (NMB, 5). LA and LMB were synthesized from the precursor, lavandulol (1) in dichloromethane, which was reacted with acetyl chloride to yield LA, and was reacted with isovaleryl chloride to yield LMB, respectively, under the catalytic activity of trimethylamine. NMB was reacted with geraniol (‘4’) and isovaleryl chloride under a catalytic activity of trimethylamine. The purity of the compounds was analyzed by gas chromatography (8860 GC, Agilent, Santa Clara, CA, USA) with a DB-1 column (15 m × 0.350 mm, Agilent) at an oven temperature of 280 ℃ and a flow rate of 1.0 mL/min.


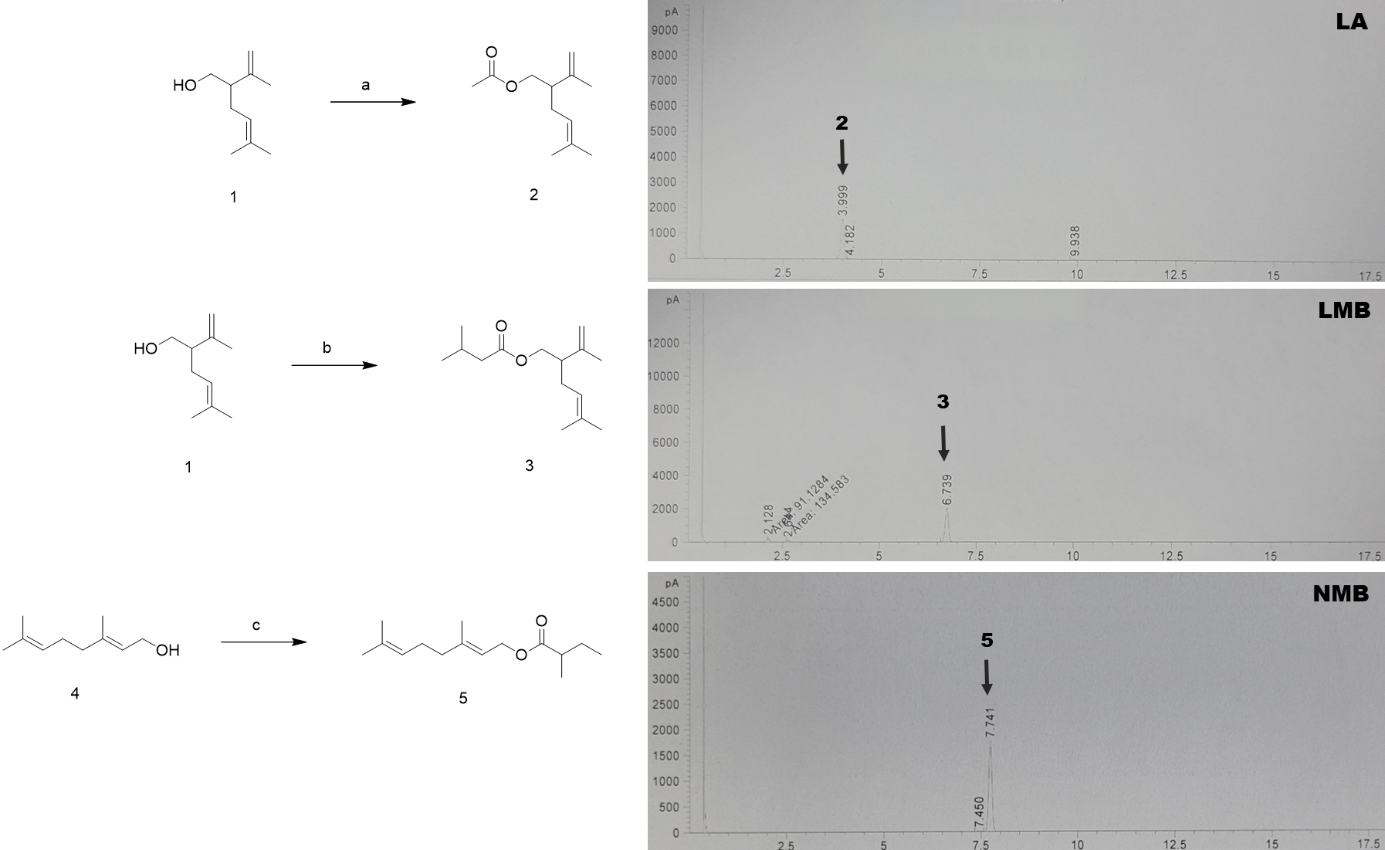


**S1 Fig**
