# Supplementary material for: A push-pull strategy to control the western flower thrips, Frankliniella occidentalis, using alarm and aggregation pheromones
Source: PLoS One. 2023 Feb 24;18(2):e0279646. doi: 10.1371/journal.pone.0279646 (PMC9956899; doi:10.1371/journal.pone.0279646)
Supplement: S2 Fig — The test pheromone components included LA (lavandulyl acetate), LMB (lavandulyl methylbutanoate), and NMB (neryl methylbutanoate). Each response (experimental unit) used 10 adults (< 3-days-old after emergence). Each treatment was replicated four times. (DOCX) [file pone.0279646.s002.docx]

**S2 Fig.** Tests of two flower thrips (*F. occidentalis* (‘Fo’) and *F. intonsa* (‘Fi’)) to aggregation pheromone components using a Y-tube olfactometer. The test pheromone components included LA (lavandulyl acetate), LMB (lavandulyl methylbutanoate), and NMB (neryl methylbutanoate). Each response (experimental unit) used 10 adults (< 3-days-old after emergence). Each treatment was replicated four times.


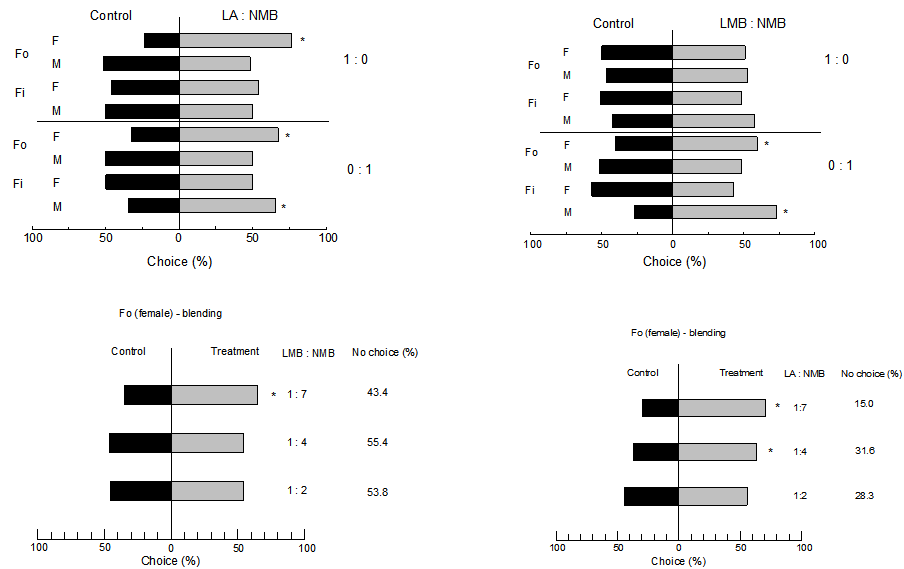


**S2 Fig**
