# Supplementary material for: A push-pull strategy to control the western flower thrips, Frankliniella occidentalis, using alarm and aggregation pheromones
Source: PLoS One. 2023 Feb 24;18(2):e0279646. doi: 10.1371/journal.pone.0279646 (PMC9956899; doi:10.1371/journal.pone.0279646)
Supplement: Attachment — Submitted filename: PONE-D-22-23616_R1 (3).pdf [file pone.0279646.s005.pdf]

PLOS ONE

A push-pull strategy to control the western flower thrips, Frankliniella occidentalis, using alarm and aggregation pheromones

--Manuscript Draft--

**Manuscript Number:**	PONE-D-22-23616R1
**Article Type:**	Research Article
**Full Title:**	A push-pull strategy to control the western flower thrips, Frankliniella occidentalis, using alarm and aggregation pheromones
**Short Title:**	Push-pull strategy
**Corresponding Author:**	Yonggyun Kim<br>Andong National University<br>Andong, KOREA, REPUBLIC OF
**Keywords:**	Frankliniella occidentalis; aggregation pheromone; alarm pheromone; push-pull strategy; hot pepper
**Abstract:**	Since the first report in 1993 in Korea, the western flower thrips, Frankliniella occidentalis, has been found in various crops throughout the country. Although more than 20 different chemical insecticides are registered to control this insect pest, its outbreaks seriously damage crop yields, especially in greenhouses. This study developed a non-chemical technique to control F. occidentalis infesting hot peppers cultivated in greenhouses. The method was based on behavioral control using an alarm pheromone ("Push") to prevent the entry of the thrips into greenhouses and an aggregation pheromone ("Pull") for mass trapping inside the greenhouses. The greenhouse fences were treated with a wax formulation of the alarm pheromone and a yellow CAN trap covered with sticky material containing the aggregation pheromone was constructed and deployed inside the greenhouses. Field assay demonstrated the efficacy of the push-pull tactics by reducing thrips density in flowers of the hot peppers as well as in the monitoring traps. Especially, the enhanced mass trapping to the CAN trap compared to the conventional yellow sticky trap led to significant reduction in the thrips population. This novel push-pull technique would be applicable to effectively control F. occidentalis in field conditions.
**Order of Authors:**	Chul-Young Kim
	Falguni Khan
	Yonggyun Kim
**Response to Reviewers:**	[Reviewer #1]<br><br>Comment #1-1: Introduction. I believe that you need a much strong justification for studying the western flower thrips, Frankliniella occidentalis as pest I did not find any strong justification for using F. occidentalis compared to other insects such as aphids for example!<br>Response: The thrips as a serious pest is explained by direct feeding damage and indirect TSWV transmission. We did not find the direct analysis on the statistical data of the relative damage compared to aphids. Thus we did not add the further information.<br><br>Comment #1-2: Materials and methods. The overall level of details for the Materials and Methods is appropriate, however there are some informations should be add in the text:<br>Line 98 page 5: Please precise if it was the same laboratory conditions described below.<br>Response: It is clarified as follows: "…. reared in the laboratory conditions."<br><br>Comment #1-3: Line 103-105 page 5: please give the purity of the molecules: Lavandulyl acetate (LA), lavandulyl methylbutanoate (LMB), and neryl methylbutanoate (NMB).<br>Response: Added

Powered by Editorial Manager® and ProduXion Manager® from Aries Systems Corporation

Comment #1-4: Line 108-110 page 6: Purity of molecules are missing here.
Response: Added

Comment #1-5: Line 114 page 6: add the purity of the alarm pheromone components
Response: added

Comment #1-6: Line 115: please correct Signal-Aldrich >> Sigma-Aldrich
Response: Corrected as suggested

Comment #1-7: Line 125: I did not find any justification for using hexane as solvent please explain.
Response: It is rephrased as follows: "To mix the pheromone with sticky material, 2 mg of pheromone components and 12 g of sticky material (Tanglefoot, Contech Electronics, Rochester, NY, USA) were dissolved in 6 mL of hexane. This mixture was then poured to a sticky plastic zipper bag (30 30 cm, HG Pack, Gunpo, Korea)."

Comment #1-8: Line 132: Give the purity of α-tocopherol.
Response: Added

Comment #1-9: Line 138: explain the use of a dark room
Response: Added as follows: "to avoid visual cue for the choice test"

Comment #1-10: Line 143: here, normally the Y-tube should be cleaned with methanol after each replication not only before and after the bio assay.
Response: As you see, we wrote the cleaning steps.

Comment #1-11: Line 143: give the sex of individuals: only females? Please justify
Response: Sex was discriminated. This information is added as follows: "Each test used 10 30 individuals of each sex and was replicated four times by changing the source and control for replication."

Comment #1-12: Line 182: information about pesticides used in fields are missing here. Please add the active molecules of these insecticides.
Response: Added as follows: "Both fields were frequently treated with chemical insecticides including spinosad during the assay."

Comment #1-13: Line 206-211: add references about used primers.
Response: Added

Comment #1-14: Line 218: reference is missing.
Response: Added

Comment #1-15: Line 220: Do you check the normality of values before using the one-way analysis of variance (ANOVA) ? please add the information.
Response: Added as follows "Percentage data were arsine-transformed and confirmed in normality using PROC UNIVARIATE in the SAS program [29]."

[Reviewer #2]

Comment #2-1: The manuscript does not provide a proper paragraph of Conclusions which must be drawn appropriately based on the data presented in the text. The data obtained in the study support the conclusions. All the experiments, trials have been conducted rigorously , with appropriate control and replication. So that also the descriptions of Material and Methods must be precse and complete. Although the manuscript has been presented in a correct and standard English, it might be useful to ask for a revision of the text by a mother language lecturer.
Response:
(1) At the end of Discussion, the conclusion is described as follows: "This control technique effectively suppressed the outbreak of F. occidentalis in a field assay and also prevented the occurrence of plant disease caused by TSWV in hot peppers. Although our current study directly evaluated the pulling efficacy of the attracting CAN trap, the pushing efficacy of the repellent fence treatment was indirectly predicted from a small-scale greenhouse experiment. Subsequent studies are needed to assess thrips occurrence outside of a test greenhouse treated with the alarm pheromone to improve

Powered by Editorial Manager® and ProduXion Manager® from Aries Systems Corporation

the estimation of the pushing efficacy in field conditions." This also explains a limitation of this technology in field conditions.
(2) Some details are added in M&M. These are marked in red color.
(3) Text is cleaned by a English-editing company, Harrisco, co.

[Reviewer #3]

Comment #3-1: Results section appears to be missing the F-values, and degrees of freedom in the reporting of the results of the ANOVA. There appear to be sections of the results section that might actually belong in the methods section, for example the details about the trap types and configuration.
Response: All F values are added. The trap was newly designed. Thus in M&M, the structure and detailed configuration were explained. In Results, the background rationale to construct the trap was explained.

Comment #3-2: The results reported for the trap comparison test use a Chi-squared value rather than an F-value which would be expected from the ANOVA that was purported to be used to analyze the results. The statistics reported in the results section do not match the statistical tests that were delineated in the methods section. Lines 304-306 only a P-value is reported, what statistical test was used here and what was the associated value? Additionally, were the data tested to confirm that they meet the assumptions of the statistical tests chosen for analysis? Did the data transformation correct for normality?
Response: All the percent data were transformed by arcsine and tested by PROC UNIVARIATE for normality. This information is added. All the F test values are added.

Comment #3-3: In the discussion section there is a lack of discussion about any potential shortcomings/weaknesses of the study and the discussion section should have more time spent discussing future directions.
Response: At the end of Discussion, the information is described as follows: "This control technique effectively suppressed the outbreak of F. occidentalis in a field assay and also prevented the occurrence of plant disease caused by TSWV in hot peppers. Although our current study directly evaluated the pulling efficacy of the attracting CAN trap, the pushing efficacy of the repellent fence treatment was indirectly predicted from a small-scale greenhouse experiment. Subsequent studies are needed to assess thrips occurrence outside of a test greenhouse treated with the alarm pheromone to improve the estimation of the pushing efficacy in field conditions." This also explains a limitation of this technology in field conditions.

Comment #3-4: The abstract could use more information regarding the results of the study.
Response: The additional information is added as follows: "Field assay demonstrated the efficacy of the push-pull tactics by reducing thrips density in flowers of the hot peppers as well as in the monitoring traps. Especially, the enhanced mass trapping to the CAN trap compared to the conventional yellow sticky trap led to significant reduction in the thrips population. This novel push-pull technique would be applicable to effectively control F. occidentalis in field conditions."

Comment #3-5: In the methods section under thrips rearing, it is stated that the thrips were fed a kidney bean diet for five days after germination. This could use some clarification as I believe the authors meant after hatching.
Response: Corrected as follows: "All thrips were fed a kidney bean (Phaseolus coccineus L.) diet according to the method described in our earlier study [12]."

Comment #3-6: Y-tube methods (line 137-146) could use more explanation. It says that thrips were used three days after emergence, is that referring to emergence from the egg (hatching), or emergence as adults from the pupal stage? Additionally, the methods state that each test used 10-30 individuals, were these individuals tested separately (one at a time in the olfactometer) or were they all places into the olfactometer at the same time, please clarify.
Response: Some uncertainty is rephrased as follows: "A Y-type olfactometer (the main Y-tube length, 5 cm; two branches 2 cm long; a 45o angle between the branches; inner branch diameter, 5 cm) was placed in a dark room at 25 ± 1oC to avoid visual cue for

the choice test. A flow of clean (charcoal-filtered) air at a rate of 0.6 L/min was split and passed through two glass vessels containing either an odor source or control (hexane) stimuli and entered each of the branches of the Y-tube. An air supply system (Power Air Pump, Seoul, Korea) was used for air filtration and flow rate control. Before and after the bioassay, the Y-tube was cleaned with 100% methanol. Test thrips were used three days after adult emergence. Each test used 10 30 individuals of each sex and was replicated four times by changing the source and control for replication.”

Comment #3-7: Line 189-190 discussing the sampling of flowers states: “in flowers in 10 randomly selected flowers was counted in three fields three times a week for 190 one month (June 13 July 13) once in a week”. This statement is confusing, was the sampling conducted three times a week or one time per week?
Response: This is rephrased as follows: “Number of F. occidentalis in flowers was counted from 10 randomly selected flowers with three replications in each field. The monitoring was performed once a week for one month (June 13 July 13).”

Comment #3-8: Line 222- should be arcsine perhaps?
Response: Corrected as suggested

Comment #3-9: In discussing the results of the field study of the push and pull components, it is stated that the Frankliniella occidentalis numbers are lower in the experimental greenhouse, but what were the results for each life stage and sex? Was it only the total number of all life stages and males and females that were reduced, or was there a difference in the response of the different life stages (larvae versus adults) or sexes?
Response: We did not examine larval density because we assessed the efficacy mostly based on the trapping adults on the yellow sticky trap. However, the figure 6C suggests the total number of larvae and adults. Thus the reduction by the push-pull can be explained by the total reduction not only by adults.

Additional Information:

Question	Response
**Financial Disclosure**<br><br>Enter a financial disclosure statement that describes the sources of funding for the work included in this submission. Review the submission guidelines for detailed requirements. View published research articles from *PLOS ONE* for specific examples.<br><br>This statement is required for submission and **will appear in the published article** if the submission is accepted. Please make sure it is accurate.	The funders had no role in study design, data collection and analysis, decision to publish, or preparation of the manuscript.

**Unfunded studies**<br>Enter: *The author(s) received no specific funding for this work.*<br><br>**Funded studies**<br>Enter a statement with the following details:<br>• Initials of the authors who received each award<br>• Grant numbers awarded to each author<br>• The full name of each funder<br>• URL of each funder website<br>• Did the sponsors or funders play any role in the study design, data collection and analysis, decision to publish, or preparation of the manuscript?<br>• **NO** - Include this sentence at the end of your statement: *The funders had no role in study design, data collection and analysis, decision to publish, or preparation of the manuscript.*<br>• **YES** - Specify the role(s) played.<br><br>* typeset	
**Competing Interests**<br><br>Use the instructions below to enter a competing interest statement for this submission. On behalf of all authors, disclose any competing interests that could be perceived to bias this work—acknowledging all financial support and any other relevant financial or non-financial competing interests.<br><br>This statement is required for submission and **will appear in the published article** if the submission is accepted. Please make sure it is accurate and that any funding sources listed in your Funding Information later in the submission form are also declared in your Financial Disclosure statement.<br><br>View published research articles from *PLOS ONE* for specific examples.	The authors have declared that no competing interests exist.

**NO authors have competing interests**<br><br>Enter: *The authors have declared that no competing interests exist.*<br><br>**Authors with competing interests**<br><br>Enter competing interest details beginning with this statement:<br><br>*I have read the journal's policy and the authors of this manuscript have the following competing interests: [insert competing interests here]*<br><br>* typeset	
**Ethics Statement**<br><br>Enter an ethics statement for this submission. This statement is required if the study involved:<br><br>• Human participants<br>• Human specimens or tissue<br>• Vertebrate animals or cephalopods<br>• Vertebrate embryos or tissues<br>• Field research<br><br>Write "N/A" if the submission does not require an ethics statement.<br><br>General guidance is provided below. Consult the submission guidelines for detailed instructions. **Make sure that all information entered here is included in the Methods section of the manuscript.**	N/A

**Format for specific study types**<br><br>**Human Subject Research (involving human participants and/or tissue)**<br>• Give the name of the institutional review board or ethics committee that approved the study<br>• Include the approval number and/or a statement indicating approval of this research<br>• Indicate the form of consent obtained (written/oral) or the reason that consent was not obtained (e.g. the data were analyzed anonymously)<br><br>**Animal Research (involving vertebrate animals, embryos or tissues)**<br>• Provide the name of the Institutional Animal Care and Use Committee (IACUC) or other relevant ethics board that reviewed the study protocol, and indicate whether they approved this research or granted a formal waiver of ethical approval<br>• Include an approval number if one was obtained<br>• If the study involved *non-human primates*, add *additional details* about animal welfare and steps taken to ameliorate suffering<br>• If anesthesia, euthanasia, or any kind of animal sacrifice is part of the study, include briefly which substances and/or methods were applied<br><br>**Field Research**<br><br>Include the following details if this study involves the collection of plant, animal, or other materials from a natural setting:<br>• Field permit number<br>• Name of the institution or relevant body that granted permission	
**Data Availability**<br><br>Authors are required to make all data underlying the findings described fully available, without restriction, and from the time of publication. PLOS allows rare exceptions to address legal and ethical concerns. See the PLOS Data Policy and FAQ for detailed information.	Yes - all data are fully available without restriction

<table>
<tr><td>A Data Availability Statement describing where the data can be found is required at submission. Your answers to this question constitute the Data Availability Statement and will be published in the article, if accepted.

Important: Stating ‘data available on request from the author’ is not sufficient. If your data are only available upon request, select ‘No’ for the first question and explain your exceptional situation in the text box.

Do the authors confirm that all data underlying the findings described in their manuscript are fully available without restriction?</td><td></td></tr>
<tr><td>Describe where the data may be found in full sentences. If you are copying our sample text, replace any instances of XXX with the appropriate details.

• If the data are held or will be held in a public repository, include URLs, accession numbers or DOIs. If this information will only be available after acceptance, indicate this by ticking the box below. For example: All XXX files are available from the XXX database (accession number(s) XXX, XXX.).
• If the data are all contained within the manuscript and/or Supporting Information files, enter the following: All relevant data are within the manuscript and its Supporting Information files.
• If neither of these applies but you are able to provide details of access elsewhere, with or without limitations, please do so. For example:

Data cannot be shared publicly because of [XXX]. Data are available from the XXX Institutional Data Access / Ethics Committee (contact via XXX) for researchers who meet the criteria for access to confidential data.

The data underlying the results presented in the study are available from (include the name of the third party</td><td>All relevant data are within the manuscript and its Supporting Information files.</td></tr>
</table>

Powered by Editorial Manager® and ProduXion Manager® from Aries Systems Corporation

and contact information or URL).<br>• This text is appropriate if the data are owned by a third party and authors do not have permission to share the data.<br><br>* typeset	
Additional data availability information:	

Powered by Editorial Manager® and ProduXion Manager® from Aries Systems Corporation

Manuscript

Click here to access/download;Manuscript;Paper (PLoS ONE)-r2.docx

PLoS ONE

A push-pull strategy to control the western flower thrips, *Frankliniella occidentalis*, using alarm and aggregation pheromones

Chul-Young Kim, Falguni Khan, and Yonggyun Kim

Department of Plant Medicals, College of Life Sciences, Andong National University, Andong 36729, Republic of Korea

Corresponding author: hosanna@anu.ac.kr

Abstract

Since the first report in 1993 in Korea, the western flower thrips, *Frankliniella occidentalis*, has been found in various crops throughout the country. Although more than 20 different chemical insecticides are registered to control this insect pest, its outbreaks seriously damage crop yields, especially in greenhouses. This study developed a non-chemical technique to control *F. occidentalis* infesting hot peppers cultivated in greenhouses. The method was based on behavioral control using an alarm pheromone ("Push") to prevent the entry of the thrips into greenhouses and an aggregation pheromone ("Pull") for mass trapping inside the greenhouses. The greenhouse fences were treated with a wax formulation of the alarm pheromone and a yellow CAN trap covered with sticky material containing the aggregation pheromone was constructed and deployed inside the greenhouses. Field assay demonstrated the efficacy of the push-pull tactics by reducing thrips density in flowers of the hot peppers as well as in the monitoring traps. Especially, the enhanced mass trapping to the CAN trap compared to the conventional yellow sticky trap led to significant reduction in the thrips population. This novel push-pull technique would be applicable to effectively control *F. occidentalis* in field conditions.

Keywords: *Frankliniella occidentalis*, aggregation pheromone, alarm pheromone, push-pull strategy, hot pepper

1. Introduction

The western flower thrips, *Frankliniella occidentalis* (Pergande) (Thysanoptera: Thripidae), is one of the most devastating insect pests to many horticultural crops including more than 500 species spanning 50 plant families, especially those cultured in greenhouses [1-3]. Both the larval and adult stages cause damage to plants by directly feeding on leaves or flowers [4,5]. Especially, adults transmit plant viruses including tomato spotted wilt virus (TSWV) [6,7]. TSWV infections have become serious in several horticultural products in Korea, including the high-value hot pepper crop [8]. This pest, originally native to North America, has spread to more than 60 countries since the late 1970s, including Europe, Asia, and Australia [9]. It was first observed on Jeju Island in 1993 and now has been spread to most regions in Korea [10].

Different reproductive modes along with a short lifecycle allow the thrips population to rapidly increase during early and late seasons, in which female progeny develop from fertilized eggs while males develop from unfertilized eggs [11,12]. The nutrient-based oogenesis of *F. occidentalis* is well-programmed in females by gonadotropic hormones including juvenile hormone, ecdysone, and prostaglandin [13,14]. To suppress thrips outbreaks, more than 20 kinds of chemical insecticides including organophosphate, carbamate, pyrethroids, neonicotinoids, avermectin, and spinosad are registered and applied to different agricultural crops [15]. However, the control efficacy against *F. occidentalis* is often unsatisfactory due to its cryptic ability to avoid exposure to the chemical spray, and the development of insecticide resistance [16]. A resistant population to emamectin benzoate showed a 356-fold increased resistance by a specific mutation in the target site and an over-expression of a detoxifying enzyme compared to a susceptible strain in Korea [17]. As alternative control tactics, the use of natural enemies, microbial pathogens, and other biological control agents has been proposed

under an integrated insect pest management (IPM) program against *F. occidentalis* [18]. In this IPM program, behavioral control using pheromones has been considered in the monitoring and mass trapping of thrips [19].

Chemical communication using pheromones plays a crucial role in mediating *F. occidentalis* populations [9]. A contact pheromone, 7-methyltricosane produced by male adults, acts as a territorial signal by eliciting a fight signal to other males or gives the location of a male to females [20]. Kirk and Hamilton [21] suggested the presence of male-producing sex pheromones attracting only females. The aggregation pheromone of *F. occidentalis* was identified as containing two components, neryl methylbutanoate (NMB) and lavandulyl acetate (LA) [22]. Kirk et al. [23] suggested another type of chemical communication in mating, an anti-aphrodisiac pheromone produced by males during mating to prevent the multiple mating of females [24]. *F. occidentalis* larvae release an alarm pheromone in an anal droplet containing decyl acetate (10:Ac) and dodecyl acetate (12:Ac), in which the two components are present in a relative mass ratio of 0.4:1 to 1.1:1, respectively [25]. This alarm signal promotes the vigilance of thrips as well as takeoff or refuge-seeking behaviors to survive predator attacks [26]. Moreover, the relative ratio of the two components and their total amounts vary with the hazard intensity by increasing the relative amount of 12:Ac or the total alarm pheromone concentration in the droplet, suggesting that the alarm signal is context-dependent [27].

Using aggregation and alarm pheromones, this study devised a push-pull control strategy against *F. occidentalis* in greenhouse conditions. Fence treatment with alarm pheromone was used to prevent the entry of thrips into the greenhouse while an aggregation pheromone was used for mass trapping inside the greenhouse. To investigate this control strategy, we tested the active pheromone compounds and determined their blend ratio in the laboratory. For field assays, both pheromones were formulated for sustainability in field conditions. Finally, the

push-pull tactic was applied to a specific farm without chemical sprays and the control efficacy was compared to that of farms that followed a conventional insecticide spraying program for thrips.

2. Materials and methods

2.1. Thrips rearing

A laboratory colony of *F. occidentalis* that originated from a local population in Jeonju in Korea was donated by the Rural Development Administration (Jeonju, Korea). It was maintained under 25 ± 1º C constant temperature and a 16:8 h (L:D) photoperiod with a relative humidity of 60 ± 5%. Another laboratory colony of *F. intonsa* was obtained from a field collection in a hot pepper field in Geosan (N36.81 E127.78, Korea) and reared in the laboratory conditions. All thrips were fed a kidney bean (*Phaseolus coccineus* L.) diet according to the method described in our earlier study [12].

2.2. Pheromones

Lavandulyl acetate (LA), lavandulyl methylbutanoate (LMB), and neryl methylbutanoate (NMB) were synthesized (AD, Inc., Andong, Korea) (S1 Fig). Briefly, LA and LMB were synthesized from the precursor, lavandulol (≥ 90%, Sigma-Aldrich Korea, Seoul, Korea) in dichloromethane, which was reacted with acetyl chloride to yield LA, and reacted with isovaleryl chloride (≥ 98%, Sigma-Aldrich Korea, Seoul, Korea) to yield LMB, respectively, under the catalytic activity of trimethylamine. NMB was used in a reaction with geraniol (>

98%, Sigma-Aldrich Korea) and isovaleryl chloride under the catalytic activity of trimethylamine. All products were extracted with ethyl acetate and produced 70 ~ 75% yields. The purity of the compounds was analyzed by gas chromatography (8860 GC, Agilent, Santa Clara, CA, USA) with a DB-1 column (15 m × 0.350 mm, Agilent), an oven temperature of 280℃, and a flow rate of 1.0 mL/min, and was determined to be 98.1% for LA, 98.1% for LMB, and 99.3% for NMB. All synthesized products were in the form of a racemic mixture. Two alarm pheromone components (decyl acetate and dodecyl acetate, 98%) were purchased from Sigma-Aldrich Korea and dissolved in hexane to prepare different concentrations.

2.3. Construction of CAN trap

The CAN trap consisted of a short supporting bar (1 m), an empty can (diameter 17 cm and height 18.5 cm), and a sticky plastic bag (see Fig 2). The short supporting bar and the empty can were connected with nails and attached to another long supporting bar (1 m) at an appropriate position for the trapping height. To mix the pheromone with sticky material, 2 mg of pheromone components and 12 g of sticky material (Tanglefoot, Contech Electronics, Rochester, NY, USA) were dissolved in 6 mL of hexane. This mixture was then poured to a sticky plastic zipper bag (30 × 30 cm, HG Pack, Gunpo, Korea). After spreading the sticky mixture with a roller, the bag was used to cover the can by inside-out inversion.

2.4. Wax formulation of alarm pheromone

The alarm pheromone (a mixture of decyl acetate and dodecyl acetate in 1.5:1, g/g) was

formulated in wax by the method of Kim et al. [28]. Briefly, paraffin wax (Merck, Rahway, NJ, USA) was dissolved and mixed with an emulsifier (Almax 3600, Illshinwells, Seoul, Korea), α-tocopherol (96%), and jojoba oil (see Fig 5A). After the alarm pheromone was added, the slurry was cooled to solidify.

2.5. Aggregation behavior test using Y-tube olfactometer

A Y-type olfactometer (the main Y-tube length, 5 cm; two branches 2 cm long; a 45° angle between the branches; inner branch diameter, 5 cm) was placed in a dark room at 25 ± 1°C to avoid visual cue for the choice test. A flow of clean (charcoal-filtered) air at a rate of 0.6 L/min was split and passed through two glass vessels containing either an odor source or control (hexane) stimuli and entered each of the branches of the Y-tube. An air supply system (Power Air Pump, Seoul, Korea) was used for air filtration and flow rate control. Before and after the bioassay, the Y-tube was cleaned with 100% methanol. Test thrips were used three days after adult emergence. Each test used 10 ~ 30 individuals of each sex and was replicated four times by changing the source and control for replication. The response was positive when the thrips passed through the midpoint of the branches for 10 min. Otherwise, the thrips' response was recorded as "no response."

2.6. Alarm pheromone test using dispersal behavior assay

An arena was designed using a Petri dish (10 × 3 cm, SPL Life Science, Seoul, Korea) to test the dispersion behavior of the thrips. Test thrips (20 individuals per replication) on the bean diet were put into the center of the dish. A disc (3-mm diameter filter paper) was put near the

diet and 10 μL of test solution in hexane was added. After 1 min, the thrips dispersed away from the bean were counted. Hexane was used as the control. Each treatment was replicated three times.

2.7. Field test for alarm pheromone

A greenhouse (220 m^2) cultivating hot peppers was used in this test. *F. occidentalis* was heavily infesting the peppers. In the middle of the greenhouse, test hot peppers in pots (one plant per pot) without any thrips were randomly assigned by a randomized block design with three replications. Each test plant was covered with a plastic cage (45 × 45 × 45 cm), which had large openings in five directions to allow the entry of nearby thrips. The wax-formulated pheromone (5 g) was loaded in a small pheromone cage (Green Agrotech, Kyungsan, Korea), which was hung at the opening gates of the cage. An empty pheromone cage was used as the control.

The duration of alarm pheromone efficacy was tested by harvesting the lure in the field conditions once a week. The efficacy test was conducted using the dispersion behavior assay described above.

2.8. Field test for push-pull tactics

A test greenhouse (660 m^2) cultivating about 200 hot peppers was located in Gudam (GD, N36.54 E128.46), Andong, Korea. Alarm pheromones in the wax formation were applied by pasting each (5 g) to the fence around the greenhouse at 2-m intervals for pushing the thrips immigrating from outside. The alarm pheromones were re-applied every four weeks. Twenty

CAN traps containing aggregation pheromone were installed inside the greenhouse for pulling the thrips. The sticky bags were replaced every week. During the assay, no chemical insecticides were applied. For reference, thrips presence was compared in two other fields cultivating hot peppers. One was a greenhouse in Hahoe (HH, about 4 km from GD) and an open field in Songchun (SC, 16 km from GD) (Fig. 6A). Both fields were frequently treated with chemical insecticides including spinosad during the assay.

2.9. Thrips monitoring test in the field

Three yellow sticky traps (Green Agrotech) were installed inside the greenhouse for monitoring changes in the thrips population from March to July. Thrips were identified based on the morphological characters described in an earlier study [12]. Number of *F. occidentalis* in flowers was counted from 10 randomly selected flowers with three replications in each field. The monitoring was performed once a week for one month (June 13 ~ July 13). TSWV infection rates in the hot peppers were measured by ring-spot disease symptoms on leaves and fruits. Each experimental unit was 100 randomly chosen hosts and was replicated three times. To determine TSWV-infected thrips, multiplex polymerase chain reaction (PCR) analysis (see below) was used to discriminate viruliferous thrips. In each site/collection, 30 thrips were used to analyze the viruliferous thrips for the period.

2.10. Molecular diagnosis of TSWV using multiplex PCR

A single insect was homogenized in 35 μL of an RNA extraction solution (LGC Bioresearch Technologies, Hoddesdon, UK) in a 1.5-ml sample tube. The homogenate was incubated at 95 ℃

for 5 min and centrifuged at 13,500 × *g* for 5 min to obtain supernatant. To extract RNA from hot peppers, a piece of a leaf (1 g) was homogenized in 250 μL of a lysis buffer in the Viral DNA/RNA Extraction Kit (Intron Biotechnology, Sungnam, Korea) and RNA was extracted according to the manufacturer's instruction. PCR amplification was performed in a total volume of 20 μL containing 4 μL of template RNA, 8 μL of primers, and 8 μL of SuPrime Script RT-PCR Premix (2X) (Genetbio, Daejeon, Korea). Seven primers [12] were designed for the TSWV (5'-TGTCTAAGGTTAAGCTCACTAAGGAA-3' and 5'-TTAAGCAAGTTCTGCAAGTATTGCCTG-3'), *F. occidentalis* (forward primer, 5'-GGT CGC TTC ACC GCT TCC CG-3'), *F. intonsa* (forward primer, 5'-GAC CAG ACT GTT CCG AGA-3'), a common reverse primer (5'-CTC TCC TGA ACW GAG GCT G-3'), and *T. tabaci* (5'-TCT AAA CAG AGG GAA AGG TG-3' and 5'-AGT GTG CCA ACA AGG CAA TG-3'). Reverse transcription was performed at 50°C for 30 min. After a denaturation step at 95 °C for 5 min, the subsequent PCR was proceeded according to the following temperature cycle program: 35 cycles of 95°C for 30 s, 55°C for 30 s, and 72°C for 1 min. The PCR products were electrophoresed on 2% (w/v) agarose gel containing 1 × TAE buffer and were visualized by UV light on a Gel Doc imaging system (Bio-Rad, Hercules, CA, USA). The resulting PCR product sizes were 777 bp for TSWV, 287 bp for *F. occidentalis*, 367 bp for *F. intonsa*, and 417 bp for *T. tabaci* [12].

2.11. Statistical analysis

Percentage data were arcsine-transformed and confirmed in normality using PROC UNIVARIATE in the SAS program [29]. Data obtained from the Y-tube or choice test were subjected to a one-way analysis of variance (ANOVA) using PROC GLM. Field assay data

were assessed by two-way ANOVA. The means were compared using the least significant difference (LSD) test at a Type I error of 0.05. Frequency data were analyzed by the mean difference test using PROC FREQ.

3. Results

3.1. Optimal pheromone composition to attract thrips

The ability of two components (LA and NMB) of the aggregation pheromone to attract thrips was tested using two thrips, *F. occidentalis* and *F. intonsa* (Fig 1). In the behavioral assays, methylbutanoate lavandulol (LMB) was used as the control (Fig 1A). LA and NMB were significantly (F = 73.50; df = 1, 6; P = 0.0001 for LA; F = 19.11; df = 1, 6; P = 0.0047 for NMB) effective in attracting adult female *F. occidentalis* and male *F. intonsa*. However, LMB did not significantly attract thrips. Then, we prepared blends containing different ratios (1:2, 1:4, and 1:7, g/g) of LA and NMB and compared their attractiveness (Fig 1B). Except for the 1:2 ratio blend, the other two blends effectively attracted both thrips species. Even though males did not show significant preferences, *F. occidentalis* females were significantly (F = 96.43; df = 1, 6; P < 0.0001) attracted to the 1:7 blend whereas *F. intonsa* females were significantly (F = 27.77; df = 1, 6; P = 0.0019) attracted to the 1:4 blend (Fig 1C).

3.2. Development of the yellow CAN trap containing aggregation pheromone

A yellow sticky trap (PLATE-type) has been used to monitor thrips. To enhance the trapping efficiency, a CAN-type trap covering an empty can with a sticky zipper bag (CAN-type) was

designed to attract thrips from all directions because the PLATE-type attracted only to the front and back sides. When the attractiveness of the PLATE-type trap was compared to that of the CAN-type trap without any aggregation pheromone treatment, they were not significantly different based on the number of thrips per sticky unit area (Fig 2A). However, the trap color significantly (F = 5.63; df = 4, 10; P = 0.0123) influenced thrips trapping, in which attractiveness was nearly lost in black traps (Fig 2B). The addition of the aggregation pheromone to the CAN trap significantly (F = 11.34; df = 1, 4; P = 0.0028) enhanced attractiveness even in a black background (Fig 2C). With a yellow background, the addition of the aggregation pheromone increased the attractiveness of the CAN trap by almost five-fold compared to the trap without the pheromone. Based on the findings, a yellow CAN trap was constructed (Fig 2D). For the CAN trap, LA (0.25 mg) and NMB (1.75 mg) were mixed with a sticky material (12 g of sticky material in 6 mL of hexane). The mixture was then poured into a zipper bag and spread using a roller. When the sticky bag was applied to the CAN trap, the sticky inside was inverted to the outside. The height of the CAN trap was controlled by the length of the supporting bar (Fig 2E).

3.3. Trapping efficiency of CAN trap in field conditions

The trapping efficiency of the CAN trap for *F. occidentalis* in field conditions was compared to that of a yellow sticky plate (PLATE), which is currently used to monitor thrips including *F. occidentalis* (Fig 3). When we compared the trapping efficiency of these two traps for a week (June 8 ~ June 15), the CAN trap showed > 4-times higher efficiency than the PLATE-type (Fig 3A). Then, we analyzed the number of *F. occidentalis* collected by the two traps over five successive weeks (Fig 3B). The collection numbers were normalized by sticky

unit area because of their different sizes. Except for one week in which thrips occurred at a low density, the CAN trap was much more attractive than the PLATE trap (t = 5.29; df = 24; P < 0.0001).

Unexpectedly, the CAN trap was also highly efficient (t = 9.99; df = 24; P < 0.0001) in attracting the whiteflies of *B. tabaci* compared to the PLATE-type. Thrips collected by the CAN trap were classified into different species and the composition was compared to that of the thrips collected by the PLATE trap (Fig 3C). The CAN trap containing the *F. occidentalis* aggregation pheromone attracted all three thrips species of *F. occidentalis*, *F. intonsa*, and *T. tabaci* but it attracted more *F. occidentalis* than the PLATE trap without pheromone (X^2 = 14.03; df = 2; P = 0.0009).

3.4. Development of thrips repellent using alarm pheromone

In response to the alarm pheromone (10:Ac and 12:Ac = 1: 1.5, g/g), larvae and adults exhibited dispersal behavior within a few seconds (Fig 4A). This dispersal behavior was also observed in response to individual components, in which 12:Ac induced the behavior in both stages, whereas 10:Ac was effective only in adults (Fig 4B). Dispersal behavior to the alarm pheromone components was highly induced in adults compared to larvae. The two-component blend was more effective in inducing the dispersal behavior than single component treatments except for the comparison of 12:Ac and the blend in the adult stage. To clarify the effectiveness between the single-component (12:Ac) and the two-component blend, different doses were applied and the efficacy of inducing dispersal behavior was assessed (Fig 4C). The two-component blend was much more effective at low doses compared to the single component in both larvae and adults. In addition, the two-component blend was effective in inducing the

dispersal behavior of *F. intonsa* (Fig 4D). However, the dispersal behavior was highly induced in *F. occidentalis* compared to *F. intonsa* at low doses. These results indicate that the alarm pheromone induced the dispersal behavior of thrips in both larvae and adults and acted as a repellent because the thrips fled the pheromone source.

The alarm pheromone-based repellent was tested in field conditions. The repellent was formulated with wax for applying by pasting (Fig 5A). To test the efficacy of the formulation, the repellents were applied to hot peppers infested with *F. occidentalis* in a greenhouse (Fig 5B). Test plants without any thrips were covered with a plastic cage, which had large openings in four directions to allow the entry of nearby thrips. The repellents were then applied to the openings. In the control without the repellent, thrips arrived within a day and maintained an immigrating population. However, hot peppers treated with the repellents maintained significantly (F = 192.79; df = 1, 34; P < 0.0001) low levels of thrips for at least two weeks (Fig 5C). The repellent efficacy of the wax formulation was maintained for four weeks without significant (F = 1600.00, df = 4, 10; P = 0.0001) differences in the greenhouse conditions.

3.4. Application of the push-pull strategy to an agricultural farm cultivating hot peppers

The occurrence of *F. occidentalis* in three different greenhouses cultivating hot peppers in Andong was compared (Fig 6). Push-pull control was applied to the GD greenhouse (Fig 6A), while HH and SC were negative controls and heavily treated with conventional insecticides, in which HH hot peppers were in a greenhouse, but SC peppers were in an open field. *F. occidentalis* adults usually occur in greenhouses in late March in Andong [12]. Our current study also found that the first adults were caught in greenhouses on March 23 in HH and April 27 in GD. In contrast, the occurrence of adults was delayed and first detected in early May in

an SC open field. After small peaks in April-May, the main adult peak was observed in June-July in all three field conditions. In the main peak, HH had many more adults than GD by more than 1,000-fold. The occurrence of *F. occidentalis* adults in GD was lower than that in the SC open field. *F. occidentalis* usually occurs at low levels because the thrips prefer greenhouse conditions over open fields [30]. To further analyze the effect of the CAN trap, thrips populations in the flowers were compared (Fig 6B). The number of *F. occidentalis* thrips in the flowers was significantly lower in CAN trap-treated GD compared to the control farms (F = 5.89; df = 10, 30; P < 0.0001). *F. occidentalis* is a vector for TSWV. The TSWV infection rate was significantly different between farms (F = 8.55; df = 2, 22; P = 0.0049). Indeed, no hot peppers were infected with TSWV in CAN trap-treated GD (Fig 6C). However, the control farms had TSWV-infected plants, in which the SC site showed an infection rate of more than 30%. This was further supported by the multiplex PCR analysis. No *F. occidentalis* was infected with TSWV, whereas significant numbers of *F. occidentalis* adults were positive for TSWV in HH and SC.

4. Discussion

This study proposes a push-pull control strategy using alarm and aggregation pheromones against *F. occidentalis* in greenhouse conditions. To test these novel control tactics, the push-pull effects of the chemical compounds were re-evaluated. For field assays, both pheromones were formulated as wax types for the alarm pheromone and a CAN trap for the aggregation pheromone. These formulated pheromones were applied to a practical farm and the control efficacy was compared to those of other conventional farms treated with chemical insecticides. Our laboratory and field data supported the control efficacy of push-pull control tactics against

F. occidentalis.

To enhance the attractiveness for *F. occidentalis,* two aggregation pheromone components (LA and NMB) were re-evaluated with an analog, lavandulyl methylbutanoate (LMB). LMB was identified as an aggregation pheromone component of another flower thrips, *Megalurothrips sjostedti* [31], and onion thrips, *Thrips palmi* [32]. No significant attractiveness of LMB for either *F. occidentalis* or *F. intonsa* was seen by Y-tube olfactometry. However, they were attracted to LA and NMB. The aggregation pheromones LA and NMB are shared between *F. occidentalis* and *F. intonsa*, so species-specific blend mixtures were used to prevent cross-attraction [33]. In our assay, *F. occidentalis* preferred a 1:7 (LA:NMB) ratio to a 1:4 ratio, whereas *F. intonsa* exhibited an opposite preference. Interestingly, a significant preference was observed only in the females of both species in the Y-tube olfactometer analysis in the double-choice experiment between 1:4 and 1:7 ratios, although both sexes were attracted to both ratios in the single-choice experiments. Considering that the aggregation pheromone is basically attractive to both sexes, our assay might not detect a male preference. The lower sensitivity of males may be their preference for a specific stereoisomer because our synthetic pheromone components (S1 Fig) were mixtures of stereoisomers. The LA:NMB ratio in *F. occidentalis* was reported to be within a range of 1:0.8 ~ 1:5 measured by solid-phase microextraction (SPME) [22]. In that study, the addition of LA to NMB gave no increase relative to NMB alone, leading to the development of a single-component commercial product. In our current study, however, the inclusion of LA in the aggregation pheromone may have had some physiological significance because unlike the 1:4 and 1:7 ratios, the 1:2 mixture ratio did not significantly attract *F. occidentalis*. This suggests the role of LA in the aggregation signal of *F. occidentalis*. Two congener species of *F. occidentalis* and *F. intonsa* share flower habitats and aggregation pheromone components, in which *F. occidentalis* had 1:13 (LA:NMB) ratio while *F. intonsa*

had 1:2 ratio in a Chinese population measured by SPME [34]. The significance of these different effective mixture ratios was confirmed in field experiments where a 1:8 ratio of aggregation pheromone components caught more *F. occidentalis,* whereas a 1:4 ratio attracted more *F. intonsa* [33]. Thus, the role of LA may give species-specificity to the aggregation pheromone. Furthermore, in our current assay, LA alone significantly attracted *F. occidentalis*. Thus, we determined the optimal mass trapping lure to be a mixture of LA and NMB at a 1:7 mixture ratio.

An alarm pheromone consisting of 10:Ac and 12:Ac induced significant dispersion behavior in *F. occidentalis*. It was reported that in response to the alarm pheromone, larvae exhibited escape behavior from the source by a rapid drop from host plants, whereas exposed adults exhibited take-off behavior and avoided landing in the area [35]. Indeed, the deployment of the alarm pheromone significantly prevented the invasion of the thrips to hot peppers in the current study. These facts led us to apply the alarm pheromone to the push-pull strategy to control *F. occidentalis*. Other potent repellents may be developed from other sources. For example, plant odors emitted by non-preferred hosts, such as garlic (*Allium sativum*) and celery (*Apium graveolens*), showed repelling effects on *F. occidentalis* females [36].

To apply the pheromones to field conditions, they were formulated to meet the purpose of the push-pull strategy. Alarm pheromone components were formulated using wax for fence treatment by pasting. The wax formulation modified the methods used in other lure formulations developed using moth sex pheromone and fly lure, where their efficacy was confirmed in field conditions [28,37]. The bioactivity of the wax formulation of the alarm pheromone lasted for at least four weeks in greenhouse conditions. Aggregation pheromone was formulated by mixing with sticky material in an attract-to-kill strategy. For this purpose, a CAN trap was devised and effectively attracted thrips by its yellow color and aggregation

pheromone. The question has been raised of whether thrips from nearby plants or thrips flying over plants were collected by the CAN traps. The study findings present indirect evidence that CAN traps collected thrips flying over the plants because adult thrips in the hot pepper flowers were not evenly distributed, whereas the 20 installed CAN traps collected similar quantities of thrips. This suggests that our CAN traps attracted flying thrips. A synergistic effect of the CAN trap and another biological control method using a natural enemy was also suggested because it is known that the predator *Orius laevigatus* stimulates the release of an alarm pheromone [27], which induces take-off flight in flying thrips in host plants.

Interestingly, the trapping efficiency of the yellow CAN trap for the whitefly *B. tabaci* was enhanced without an aggregation pheromone. This suggests that the aggregation pheromone of *F. occidentalis* was effective in attracting *B. tabaci*. The two aggregation pheromone components are terpenoids. Whiteflies respond to various terpenoids including sesquiterpenes (zingiberene and curcumene) and monoterperpenes (*p*-cymene, *α*-terpinene, and *α*-phellandrene), although terpenoids act as repellents [38]. It is noteworthy that LMB, one of the aggregation pheromone components in thrips, is used by a sex pheromone of the mealybug, *Phenacoccus madeirensis* [39]. Further studies assessing the sensitivity and response of *B. tabaci* to the aggregation pheromone of *F. occidentalis* need to be conducted.

The push-pull strategy was effective in suppressing the frequency of TSWV-infected hot peppers. Compared to other fields such as those in HH and SC, the thrips density was low in flowers in GD treated with the push-pull strategy, as was the number in monitoring traps. TSWV-infected hot peppers exhibit characteristic disease symptoms of ring-spots on leaves and fruit [40]. Based on this disease symptom, the survey indicated no TSWV infections in hot peppers in GD in contrast to the significant numbers of disease symptoms in the other two fields. This was further supported by the multiplex PCR results, which indicated no TSWV-

infected *F. occidentalis* in GD compared to large numbers of TSWV-positive thrips in other fields. This might be explained by a local variation in viruliferous thrips as similar variations in *Thrips tabaci* carrying TSWV were seen [41]. Alternatively, the suppression of thrips occurrence by the push-pull treatment might have caused the decrease in the TSWV infection rate.

The push-pull strategy has been used for effective insect pest control [42]. A push-pull control technique against *F. occidentalis* was proposed using UV-reflective mulch as a pushing factor to disrupt thrips colonization on host plants along with a companion plant as a pulling factor to attract the predators and trap the thrips [43]. Alternatively, the use of semiochemicals such as attractants and repellents has been considered for developing a push-pull strategy [44]. Kirk et al. [23] proposed *F. occidentalis* control using pheromones and other semiochemicals. This study demonstrated novel push-pull tactics using aggregation/alarm pheromones (Fig 7) to control *F. occidentalis* in greenhouse conditions. This control technique effectively suppressed the outbreak of *F. occidentalis* in a field assay and also prevented the occurrence of plant disease caused by TSWV in hot peppers. Although our current study directly evaluated the pulling efficacy of the attracting CAN trap, the pushing efficacy of the repellent fence treatment was indirectly predicted from a small-scale greenhouse experiment. Subsequent studies are needed to assess thrips occurrence outside of a test greenhouse treated with the alarm pheromone to improve the estimation of the pushing efficacy in field conditions.

Supplementary information

S1 Fig. Chemical synthesis of the aggregation pheromone components of thrips: lavandulyl

acetate (LA, 2), lavandulyl methylbutanoate (LMB, 3), and neryl methylbutanoate (NMB, 5). LA and LMB were synthesized from the precursor, lavandulol (1) in dichloromethane, which was reacted with acetyl chloride to yield LA, and was reacted with isovaleryl chloride to yield LMB, respectively, under the catalytic activity of trimethylamine. NMB was reacted with geraniol (‘4’) and isovaleryl chloride under a catalytic activity of trimethylamine. The purity of the compounds was analyzed by gas chromatography (8860 GC, Agilent, Santa Clara, CA, USA) with a DB-1 column (15 m × 0.350 mm, Agilent) at an oven temperature of 280 ℃ and a flow rate of 1.0 mL/min.

S2 Fig. Tests of two flower thrips (*F. occidentalis* (‘Fo’) and *F. intonsa* (‘Fi’)) to aggregation pheromone components using a Y-tube olfactometer. The test pheromone components included LA (lavandulyl acetate), LMB (lavandulyl methylbutanoate), and NMB (neryl methylbutanoate). Each response (experimental unit) used 10 adults (< 3-days-old after emergence). Each treatment was replicated four times.

Acknowledgements

We thank Jean Lee and Juan Hong in our laboratory to provide thrips for our analysis.

Author Contributions

Conceptualization: Yonggyun Kim.

Formal analysis: Chul-Young Kim, Falguni Khan, Yonggyun Kim.

Funding acquisition: Yonggyun Kim.

Investigation: Chul-Young Kim, Falguni Khan, Yonggyun Kim.

Methodology: Chul-Young Kim, Falguni Khan, Yonggyun Kim.

Project administration: Yonggyun Kim.

Validation: Chul-Young Kim.

Writing – original draft: Chul-Young Kim, Yonggyun Kim.

Writing – review & editing: Yonggyun Kim.

References

1. Yudin LS, Cho JJ, Mitchell WC. Host range of western flower thrips, *Frankliniella occidentalis* (Thysanoptera: Thripidae), with special reference to *Leucaena glauca*. Environ Entomol. 1986; 15:1292-1295.
2. Schneweis DJ, Whitfield AE, Rotenberg D. Thrips developmental stage-specific transcriptome response to tomato spotted wilt virus during the virus infection cycle in *Frankliniella occidentalis*, the primary vector. Virology. 2017; 500:226–237.
3. He Z, Guo JF, Reitz SR, Lei ZR, Wu SY. A global invasion by the thrips, *Frankliniella occidentalis*: current virus vector status and its management. Insect Sci. 2020; 27:626-645.
4. Reitz SR. Biology and ecology of the western flower thrips (Thysanoptera: Thripidae): The making of a pest. Fla Entomol. 2009; 92:7-13.
5. Shrestha A, Srinivasan R, Riley DG, Culbreath AK. Direct and indirect effects of a thrips-transmitted Tospovirus on the preference and fitness of its vector, *Frankliniella fusca*. Entomol Exp Appl. 2012; 145:260-271.

6. Belliure B, Janssen A, Maris PC, Peters D, Sabelis MW. Herbivore arthropods benefit from vectoring plant viruses. Ecol Lett. 2005; 8:70-79.

7. Rotenberg D, Jacobson AL, Schneweis DJ, Whitfield AE. Thrips transmission of tospoviruses. Curr Opin Virol. 2015; 15:80-89.

8. Lee S, Lee J, Kim S, Choi H, Park J, Lee J, et al. The incidence and distribution of viral diseases in pepper by cultivation types. Res Plant Dis. 2004; 10:231-240.

9. Reitz SR, Gao Y, Kirk WDJ, Hoddle MS, Leiss KA, Funderburk JE. Invasion biology, ecology, and management of western flower thrips. Annu Rev Entomol. 2020; 65:17-37.

10. Woo KS, Ahn SB, Lee SH, Kwon HM. First record of *Frankliniella occidentalis* and its distribution and host plants in Korea. Korean J Appl Entomol. 1994; 33:127.

11. Ding T, Chi H, Gökçe A, Gao Y, Zhang B. Demographic analysis of arrhenotokous parthenogenesis and bisexual reproduction of *Frankliniella occidentalis* (Pergande) (Thysanoptera: Thripidae). Sci Rep. 2018; 8:3346.

12. Kim C, Choi D, Kang J, Ahmed SA, Kil E, Kwon G, et al. Thrips infesting hot pepper cultured in greenhouses and variation in gene sequences encoded in TSWV. Korean J Appl Entomol. 2021; 60:387-401.

13. Choi DY, Kim Y. PGE_2 mediation of egg development in western flower thrips, *Frankliniella occidentalis*. Arch Insect Biochem Physiol. 2022; e21949.

14. Choi DY, Kim Y. Transcriptome analysis of female western flower thrips, *Frankliniella occidentalis*, exhibiting neo-panoistic ovarian development. PLoS ONE 2022; 17:e0272399.

15. Cho S, Kyung Y, Cho S, Shin S, Jeong D, Kim S, et al. Evaluation of susceptibility of western flower thrips (*Frankliniella occidentalis*) and garden thrips (*F. intonsa*) to 51 insecticides. Korean J Appl Entomol. 2018; 57:221-231.

16. Bhuyain MMH, Lim UT. Relative susceptibility to pesticides and environmental conditions of *Frankliniella intonsa* and *F. occidentalis* (Thysanoptera: Thripidae), an underlying reason for their asymmetrical occurrence. PLoS ONE 2020; 15:e0237876.

17. Gao Y, Yoon KA, Lee JH, Kim JH, Lee SH. Overexpression of glutamate-gated chloride channel in the integument is mainly responsible for emamectin benzoate resistance in the western flower thrips *Frankliniella occidentalis*. Pest Manag. Sci. 2022; doi: 10.1002/ps.7032.

18. Mouden S, Sarmiento KF, Klinkhamer PG, Leiss KA. Integrated pest management in western flower thrips: past, present and future. Pest Manag Sci. 2017; 73:813-822.

19. Sampson C, Kirk WD. Can mass trapping reduce thrips damage and is it economically viable? Management of the Western flower thrips in strawberry. PLoS ONE 2013; 8:e80787.

20. Olaniran OA, Sudhakar AV, Drijfhout FP, Dublon IA, Hall DR, Hamilton JG, et al. A male-predominant cuticular hydrocarbon, 7-methyltricosane, is used as a contact pheromone in the western flower thrips *Frankliniella occidentalis*. J Chem Ecol. 2013; 39:559-568.

21. Kirk WD, Hamilton JG. Evidence for a male-produced sex pheromone in the western flower thrips *Frankliniella occidentalis*. J Chem Ecol. 2004; 30:167-174.

22. Hamilton JG, Hall DR, Kirk WD. Identification of a male-produced aggregation pheromone in the western flower thrips *Frankliniella occidentalis*. J Chem Ecol. 2005; 31:1369-1379.

23. Kirk WDJ, de Kogel WJ, Koschier EH, Teulon DAJ. Semiochemicals for thrips and their use in pest management. Annu Rev Entomol. 2021; 66:101-119.

24. Akinyemi AO, Kirk WDJ. Experienced males recognise and avoid mating with non-virgin females in the western flower thrips. PLoS ONE 2019; 14:e0224115.

25. MacDonald KM, Hamilton JG, Jacobson R, Kirk WD. Analysis of anal droplets of the western flower thrips *Frankliniella occidentalis*. J Chem Ecol. 2003; 29:2385-2389.

26. de Bruijn PJ, Egas M, Janssen A, Sabelis MW. Pheromone-induced priming of a defensive response in Western flower thrips. J Chem Ecol. 2006; 32:1599-1603.

27. de Bruijn PJ, Egas M, Sabelis MW, Groot AT. Context-dependent alarm signalling in an insect. J Evol Biol. 2016; 29:665-671.

28. Kim K, Kim M, Kwon G, Kim Y. Technologies required for development of trap-based MAT control against the striped fruit fly, *Bactrocera scutellata*. Korean J. Appl. Entomol. 2017; 56:51-60.

29. SAS Institute Inc. SAS/STAT user's guide, Release 6.03, Ed. Cary, NC. 1989.

30. Lee GS, Lee JH, Kang SH, Woo KS. Thrips species (Thysanoptera: Thripidae) in winter season and their vernal activities on Jeju island, Korea. J Asia Pac Entomol. 2001; 4:115–122.

31. Niassy S, Tamiru A, Hamilton JGC, Kirk WDJ, Mumm R, Sims C, et al. Characterization of male-produced aggregation pheromone of the bean flower thrips *Megalurothrips sjostedti* (Thysanoptera: Thripidae). J Chem Ecol. 2019; 45:348-355.

32. Akella SVS, Kirk WDJ, Lu YB, Murai T, Walters KFA, Hamilton JG. Identification of the aggregation pheromone of the melon thrips, *Thrips palmi*. PLoS ONE 2014; 9:e103315.

33. Li X, Geng S, Zhang Z, Zhang J, Li W, Huang J, et al. Species-specific aggregation pheromones contribute to coexistence in two closely related thrips species. Bull Entomol Res. 2019; 109:119-126.

34. Zhu XY, Zhang PJ, Lu YB. Isolation and identification of the aggregation pheromone released by male adults of *Frankliniella intonsa* (Thysanoptera: Thripidae). Acta Entomol Sin. 2012; 55:376-385.

35. Teerling CR, Pierce HD, Borden JH, Gillespie DR. Identification and bioactivity of alarm pheromone in the western flower thrips, *Frankliniella occidentalis*. 1993; 19:681-697.

36. Cao Y, Zhi J, Cong C, Margolies DC. Olfactory cues used in host selection by *Frankliniella occidentalis* (Thysanoptera: Thripidae) in relation to host suitability. J Insect Behav. 2014; 27:41-56.

37. Jung S, Park M, Lee S, Choi K, Hong Y, Bae S. et al. Development of wax-typed pheromone dispenser for mating disruption of the oriental fruit moth, *Grapholita molesta*, and its application technique. Korean J Appl Entomol. 2008; 47:255-263.

38. Bleeker PM, Diergaarde PJ, Ament K, Guerra J, Weidner M, Schütz S, et al. The role of specific tomato volatiles in tomato-whitefly interaction. Plant Physiol. 2009; 151:925-935.

39. Ho HY, Su YT, Ko CH, Tsai MY. Identification and synthesis of the sex pheromone of the Madeira mealybug, *Phenacoccus madeirensis* Green. J Chem Ecol. 2009; 35:724-732.

40. Lee JS, Cho WK, Kim MK, Kwak HR, Choi HS, Kim KH. Complete genome sequences of three tomato spotted wilt virus isolates from tomato and pepper plants in Korea and their phylogenetic relationship to other TSWV isolates. Arch Virol. 2011; 156:725-728.

41. Jacobson AL, Kennedy GG. Specific insect-virus interactions are responsible for variation in competency of different *Thrips tabaci* isolines to transmit different Tomato Spotted Wilt Virus isolates. PLoS ONE 2013; 8:e54567.

42. Cook SM, Khan ZR, Pickett JA. The use of push-pull strategies in integrated pest management. Annu Rev Entomol. 2007; 52:375-400.

43. Tyler-Julian K, Funderburk J, Srivastava M, Olson S, Adkins S. Evaluation of a push-pull system for the management of *Frankliniella* species (Thysanoptera: Thripidae) in tomato. Insects 2018; 9:187.

44. Niu Y, Han S, Wu Z, Pan C, Wang M, Tang Y. et al. A push-pull strategy for controlling the tea green leafhopper (*Empoasca flavescens* F.) using semiochemicals from *Tagetes erecta* and *Flemingia macrophylla*. Pest Manag Sci. 2022; 78:2161-2172.

Figure captions

Fig 1. **Differential preference analysis of two flower thrips (*F. occidentalis* and *F. intonsa*) for aggregation pheromone using a Y-tube olfactometer.** (A) Screening of active components and mixture ratios in the aggregation pheromone. The pheromone components were identified as LA (lavandulyl acetate), LMB (lavandulyl methylbutanoate), and NMB (neryl methylbutanoate). (B) Choice tests of the thrips against two pheromone blends. Each response (experimental unit) used 20 adults (< 3-days-old after emergence). Each treatment was replicated four times. The detailed data are presented in Figure S1.

Fig 2. **Development of a yellow CAN trap (CAN) to attract *F. occidentalis* using the aggregation pheromone.** (A) Comparison of the attractiveness of the CAN trap without aggregation pheromone and a yellow sticky trap (PLATE). (B) Effect of CAN trap color on attracting thrips. (C) Effect of aggregation pheromone on attracting thrips using the CAN trap, in which the black CAN trap demonstrated a pheromone effect while the yellow CAN trap demonstrated both pheromone and color effects. Pheromone components were prepared by mixing lavandulyl acetate (LA) and neryl methylbutanoate (NMB) at a 1:7 mass ratio. Each experimental unit represented a trap and was replicated three times. (D) Preparation of a sticky bag for the CAN trap. (E) Dimensions (mm) of the CAN trap parts and adjustment of the CAN trap height.

Fig 3. **Field efficacy of a yellow CAN trap containing aggregation pheromone.** (A) Enhanced trapping efficiency of the CAN trap containing aggregation pheromone compared to the yellow sticky plate (PLATE). CAN and PLATE traps used 20 and three replications,

respectively. An asterisk above the standard deviation bars indicates a significant difference between two treatments at type I error = 0.05 (LSD test). (B) Enhanced pulling effect of the CAN trap on trapping *F. occidentalis* and *B. tabaci* in different seasons. Two and three asterisks above the standard deviation bars indicate significant differences between two treatments at Type I error = 0.01 and type I error = 0.0001 (LSD test), respectively. NS indicates no significant difference. (C) Composition of thrips trapped by the PLATE and CAN traps. The average species frequencies in CAN and PLATE traps were obtained from 20 and three replications, respectively.

Fig 4. **Development of a thrips repellent using an alarm pheromone of *F. occidentalis*.** The alarm pheromone components were decyl acetate (10:Ac) and dodecyl acetate (12:Ac) at a 1:1.5 mass ratio. (A) Bioassay assessing repellent efficacy measured by the dispersal behavior of adults and larvae from bean diet after the test component (10 μL) was dispensed onto a filter paper disc. Arrows indicate the dispersal behavior of larvae. Different letters above the standard deviation bars indicate a significant difference between means at type I error = 0.05 (LSD test). (B) Repellent efficacy of single and dual components. (C) Dose-response of the pheromone components. (D) Comparison of dual component pheromone (alarm pheromone) efficacy in *F. occidentalis* (Fo) and *F. intonsa* (Fi). Each treatment was replicated three times. Asterisks above the standard deviation bars indicate significant difference between two treatments (LSD test): * at Type I error = 0.05, ** at Type I error = 0.01, and *** at Type I error = 0.0001. NS indicates no significant difference.

Fig 5. **Field assay of alarm pheromone-based repellent efficacy against *F. occidentalis* in a greenhouse cultivating hot peppers.** (A) Composition of the wax formulation of the

repellent and installation of the wax formulations (in a small cage) in five directions around a test plant. (B) Effect of the repellent on preventing immigrating thrips for two weeks. Three asterisks above the standard deviation bars indicate a significant difference between two treatments at Type I error = 0.0001 (LSD test). (C) Determination of the effective period of the wax formulation in field conditions. Different letters above the standard deviation bars indicate a significant difference among means at Type I error = 0.05 (LSD test).

Fig 6. **Application of push-pull strategy to *F. occidentalis* infesting hot peppers (200 plants) in a greenhouse (660 m^2).** (A) Deployment of repellents and CAN traps in a GD greenhouse. Adult occurrence in three sites. (B) Number of *F. occidentalis* in flowers. Each replication represents the total number of thrips from 10 randomly selected flowers. Each measurement was replicated three times. (C) TSWV infection rates in hot peppers and *F. occidentalis* over one month (June 13 to July 13). Each measurement represents the percentage of diseased plants out of 100 randomly chosen hosts. Each measurement was replicated three times. To determine TSWV-infected thrips, multiplex PCR analysis was performed to discriminate viruliferous thrips. In each site/collection, 30 thrips were used to analyze the viruliferous thrips for the period. An asterisk above the standard deviation bars indicates a significant difference among means at Type I error = 0.05 (LSD test). NS indicates no significant difference.

Fig 7. **A working model of the push-pull strategy against *F. occidentalis* using repellent (sound signal logo) and CAN trap (yellow can) in a greenhouse cultivating hot peppers.** Arrows indicate the push and pull moving directions.

Figure 1

Click here to access/download;Figure;Figure1.jpg

(A)

Components	Response[1] of *F. occidentalis*		Response[1] of *F. intonsa*	
	Females	Males	Females	Males
NMB	+	NS	NS	+
LA	+	NS	NS	NS
LMB	NS	NS	NS	NS

LA:NMB (g/g)	Response[1] of *F. occidentalis*		Response[1] of *F. intonsa*	
	Females	Males	Females	Males
1:2	NS	NS	NS	NS
1:4	+	+	+	+
1:7	+	+	+	+

[1]+represents significant ($P < 0.05$) preference for the test compound and NS indicates no significant difference.

(B)

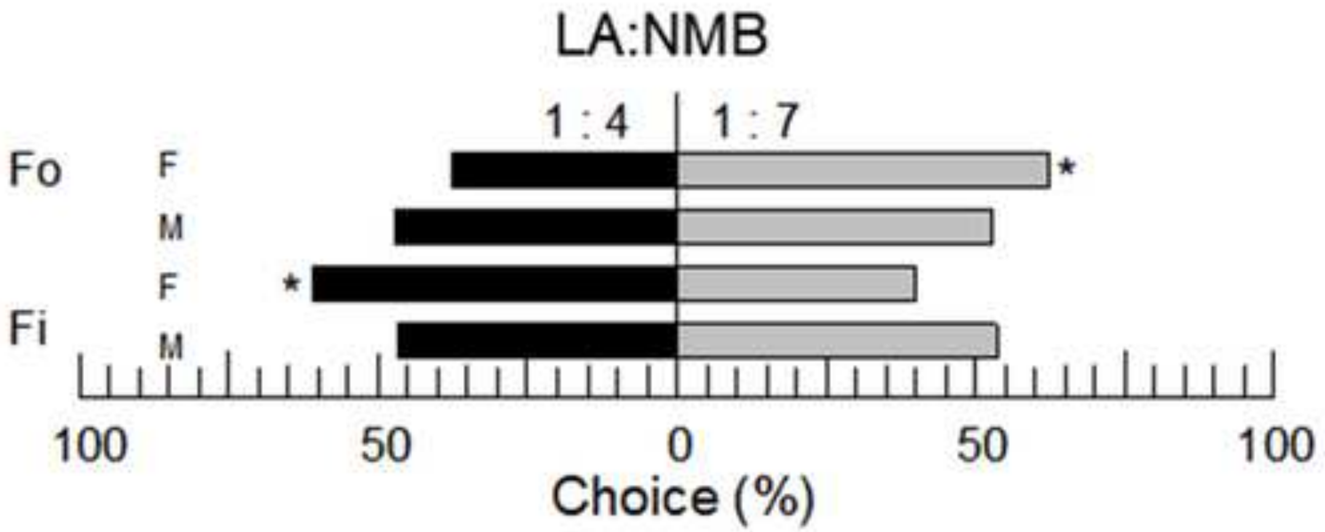

Figure 2

Click here to access/download;Figure;Figure2.jpg

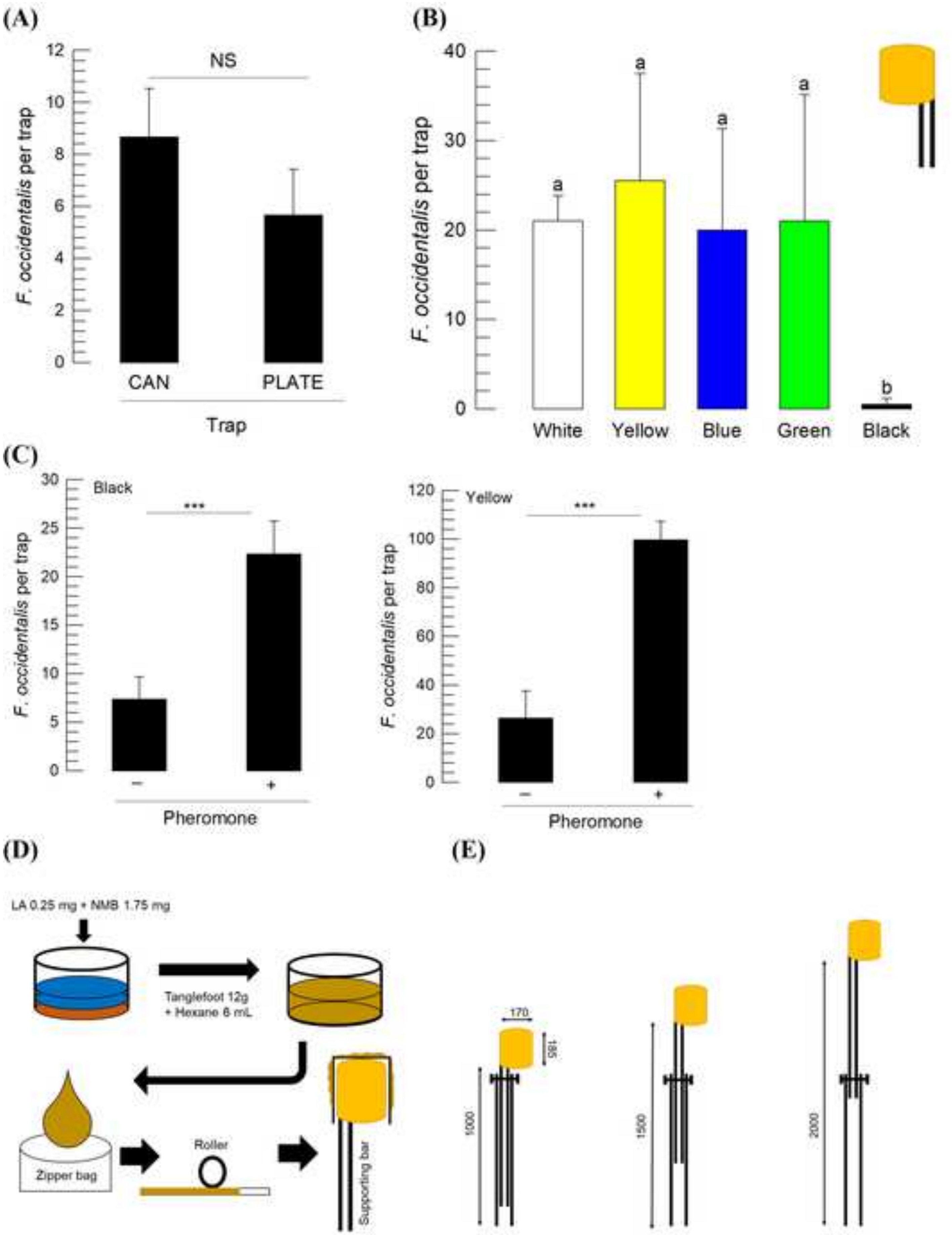

Figure 3

Click here to access/download;Figure;Figure3.jpg

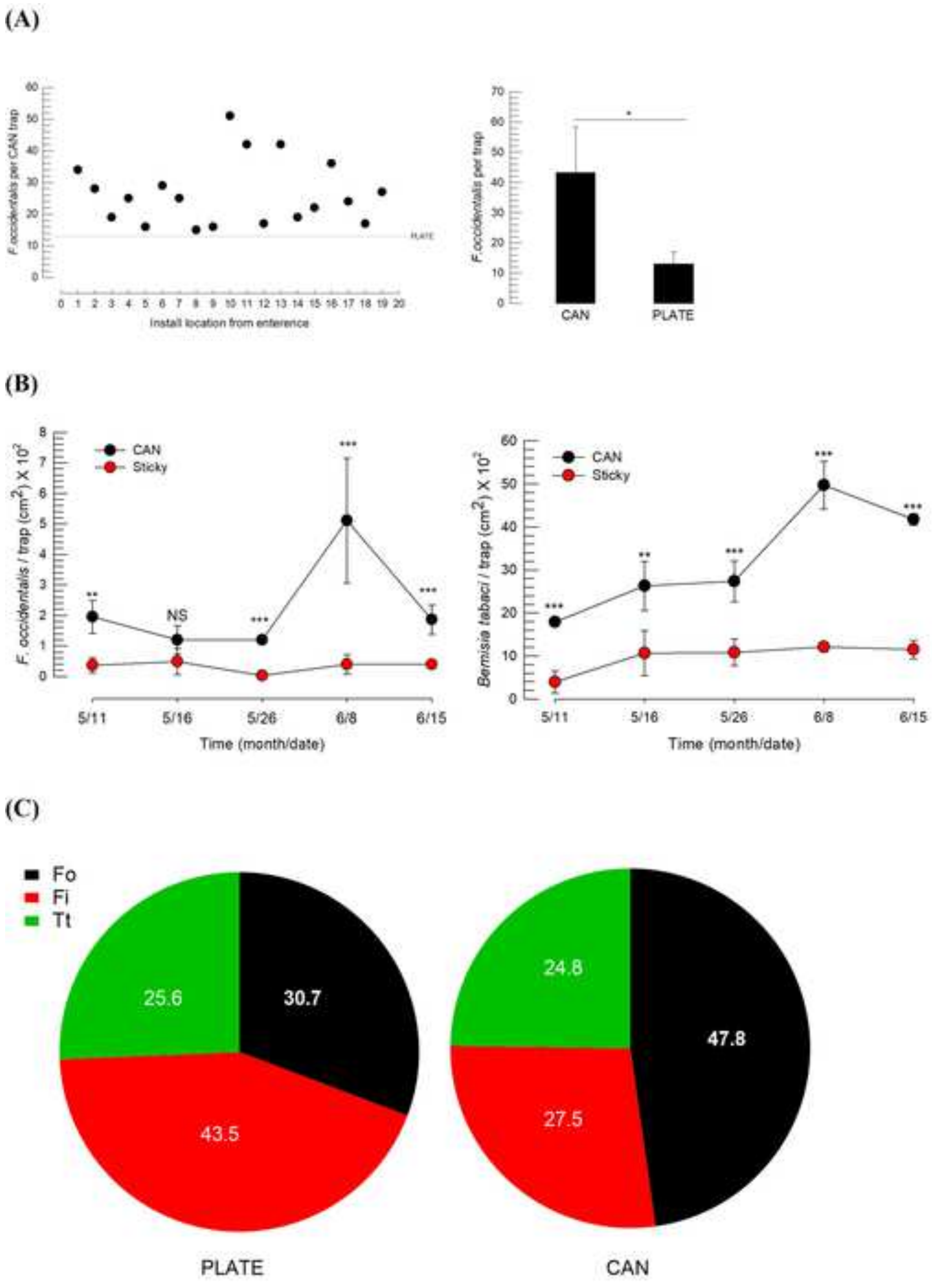

Figure 4 Click here to access/download;Figure;Figure4.jpg

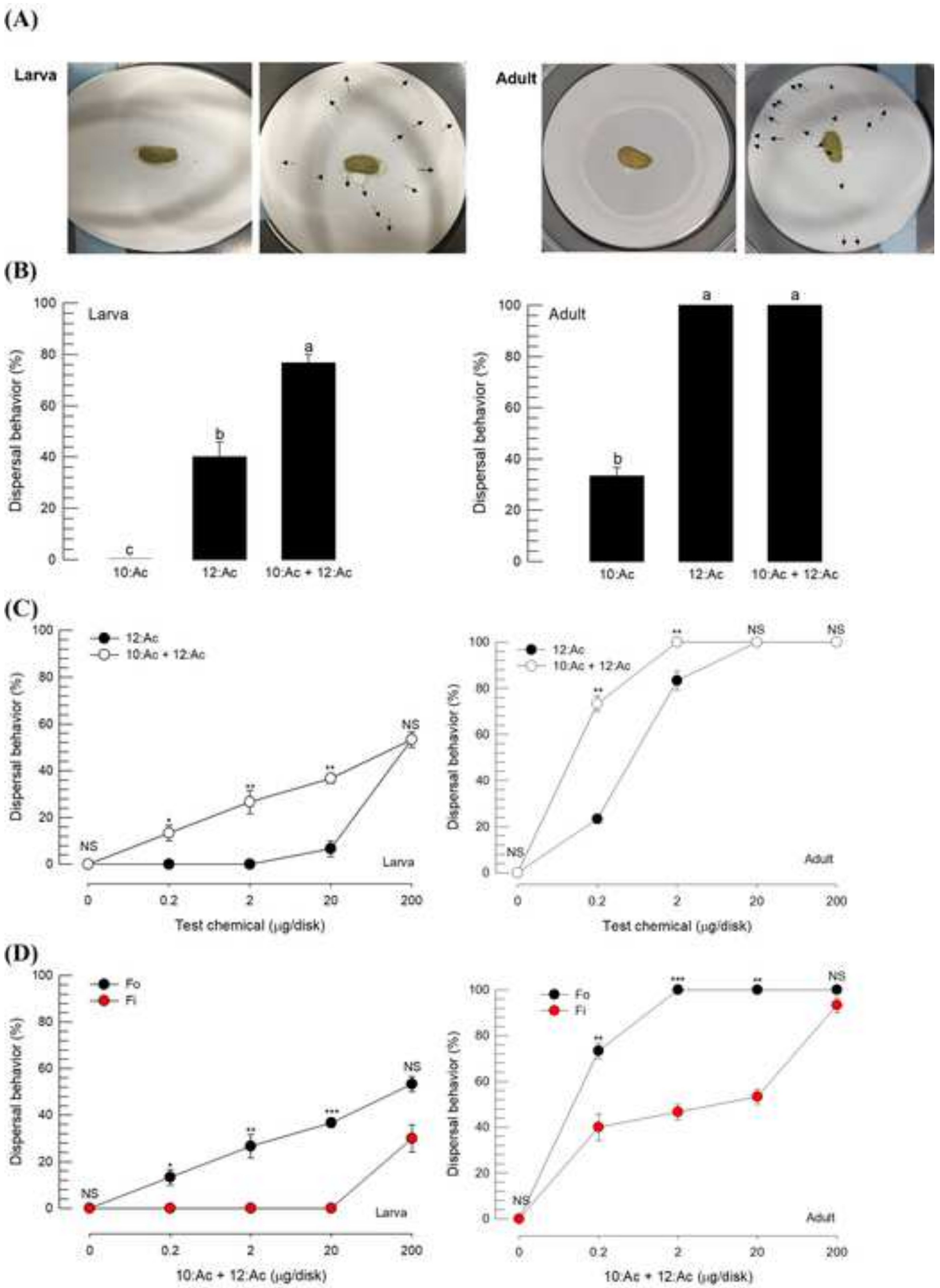

Click here to access/download;Figure;Figure5.jpg

(A)

Components	Weight or volume
Paraffin wax	30 g
Emulsifier (Almax 3600)	6 g
α-Tocopherol	2 g
Jojoba oil	2 g
Dodecyl acetate	1.5 mL
Decyl acetate	1 mL
Distilled water	48 mL

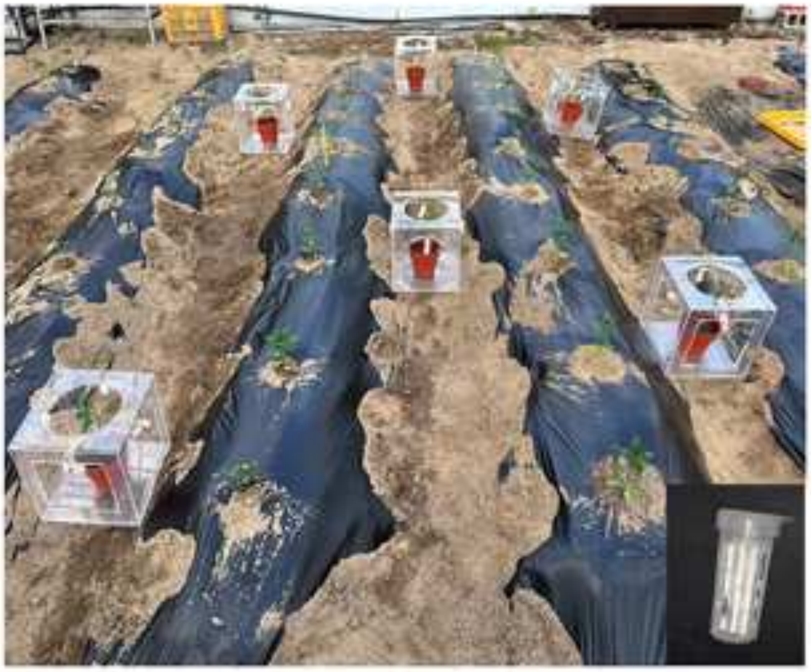

(B)

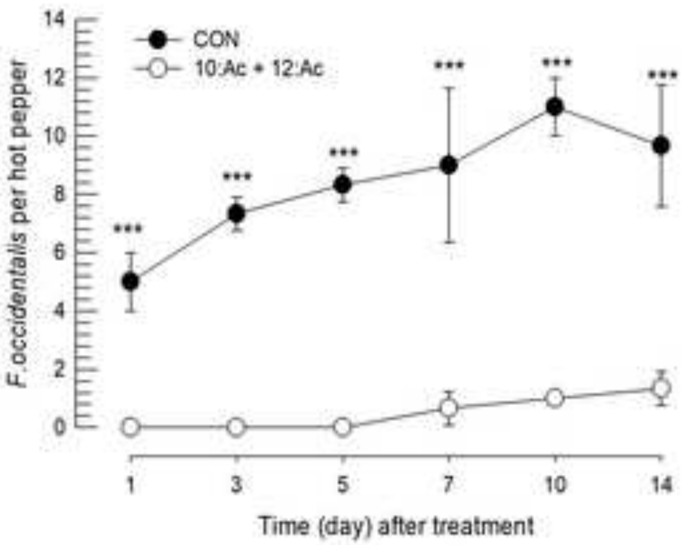

(C)

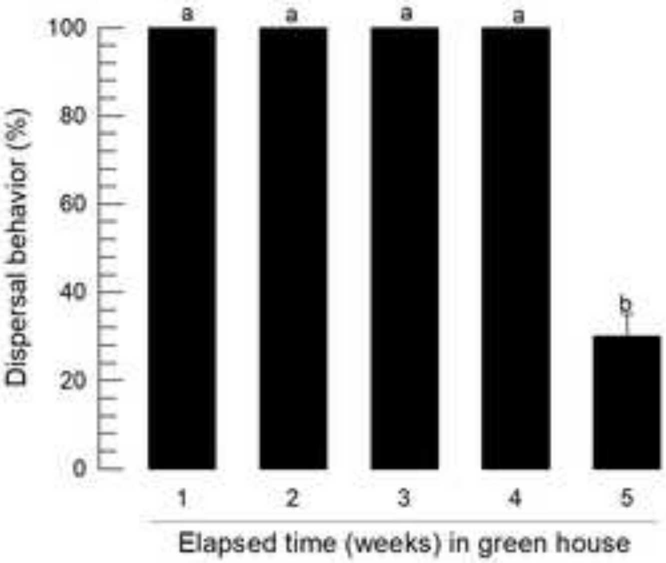

Figure 6A

Click here to access/download;Figure;Figure6A.jpg

(A)

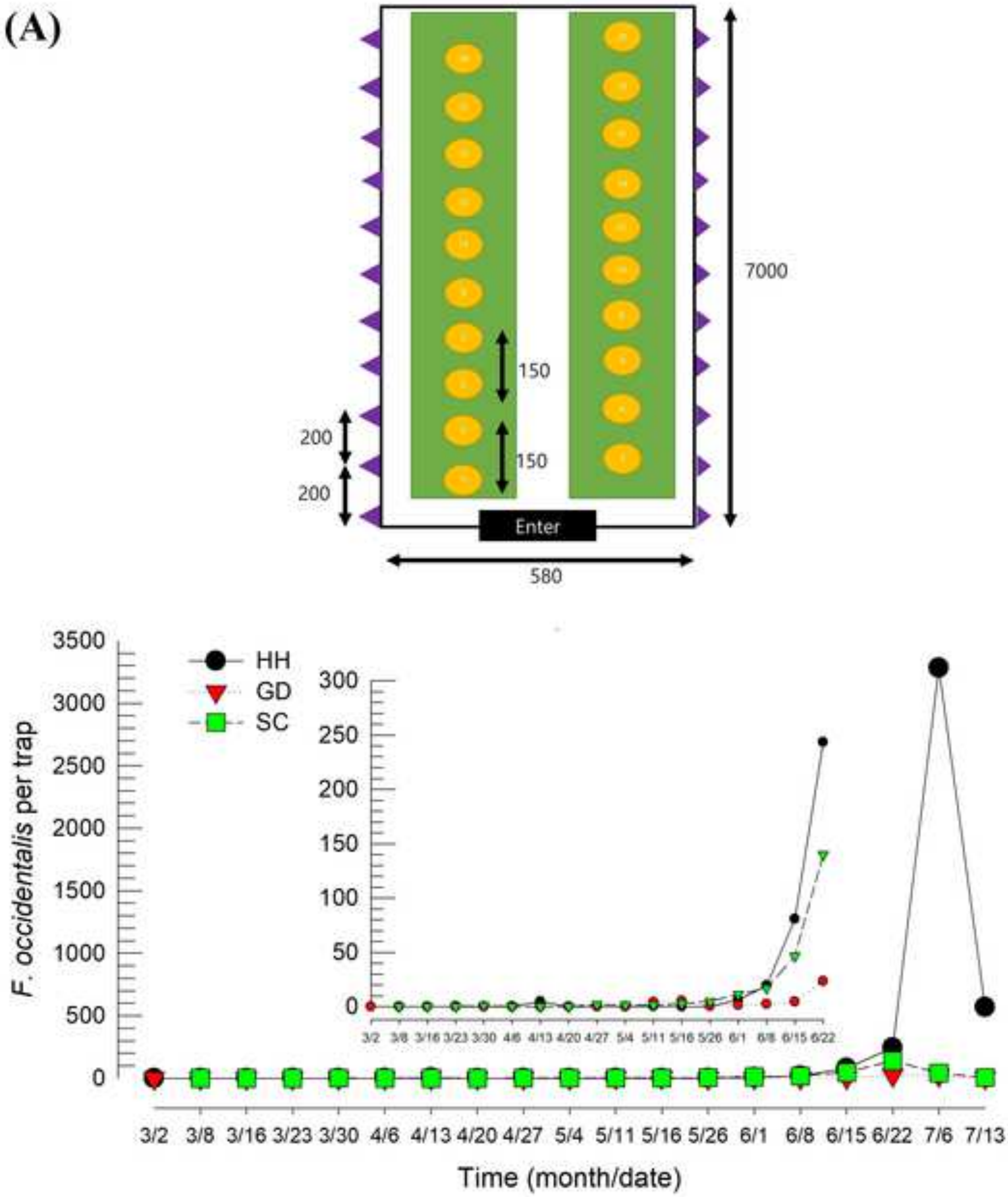

(B)

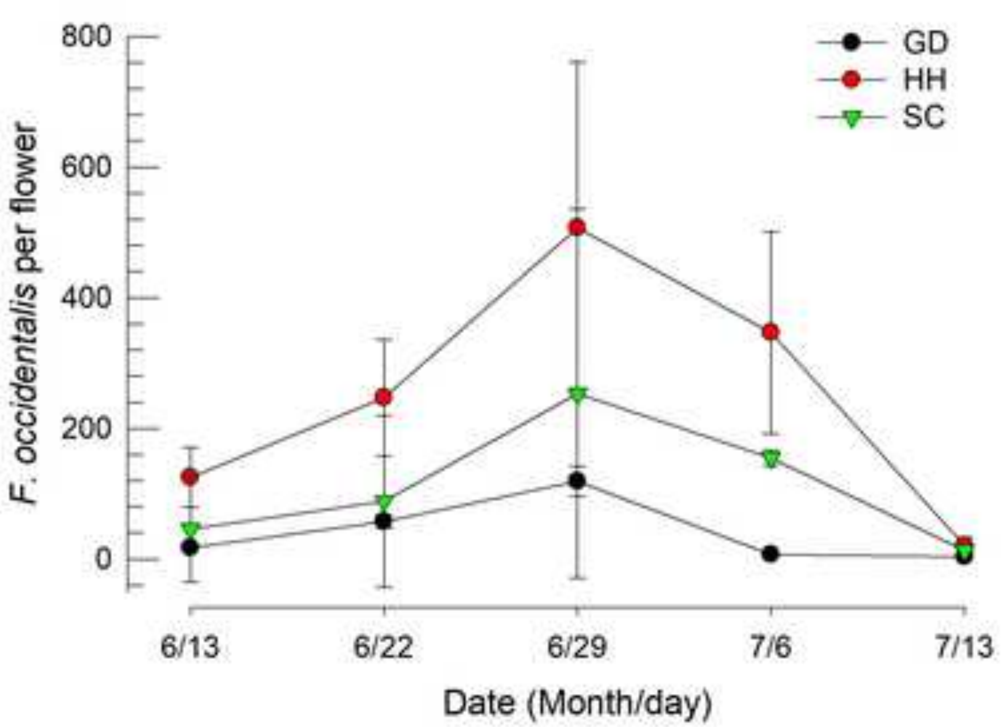

800
600
400
200
0
F. occidentalis per flower
GD
HH
SC
6/13
6/22
6/29
7/6
7/13
Date (Month/day)

(C)

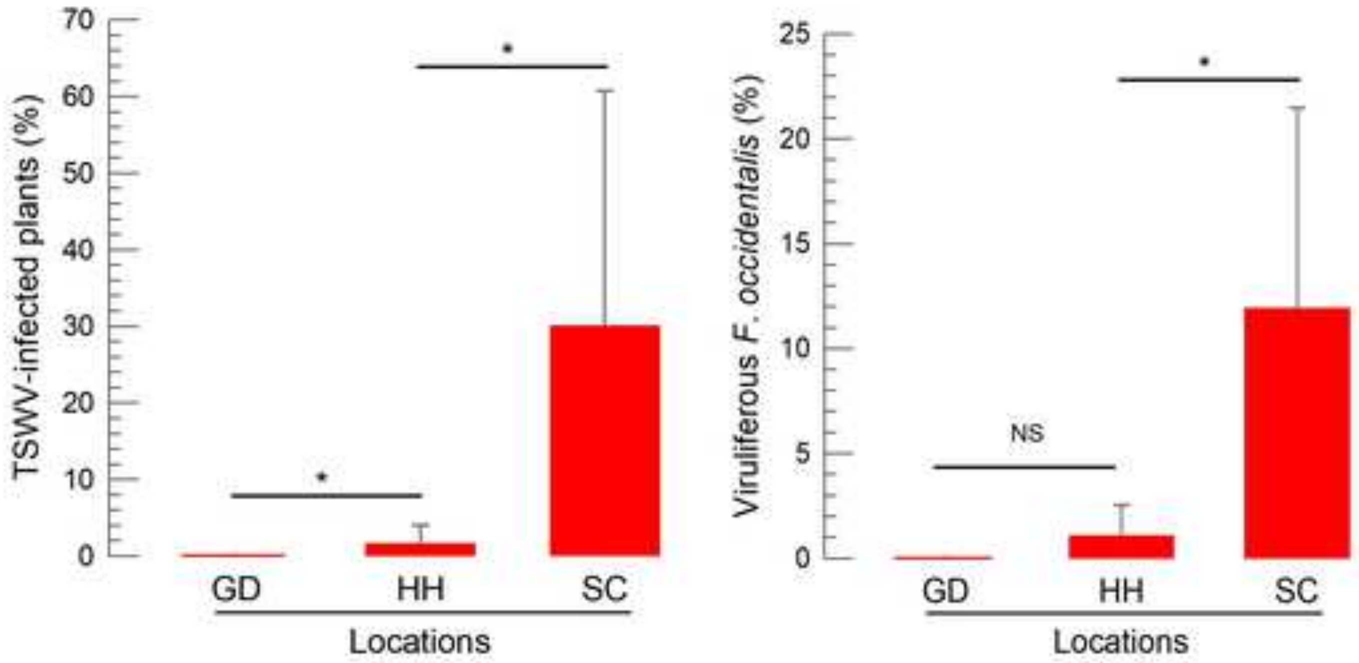

70
60
50
40
30
20
10
0
TSWV-infected plants (%)
*
*
GD
HH
SC
Locations
25
20
15
10
5
0
Viruliferous F. occidentalis (%)
NS
*
GD
HH
SC
Locations

Figure 7

Click here to access/download;Figure;Figure7.jpg

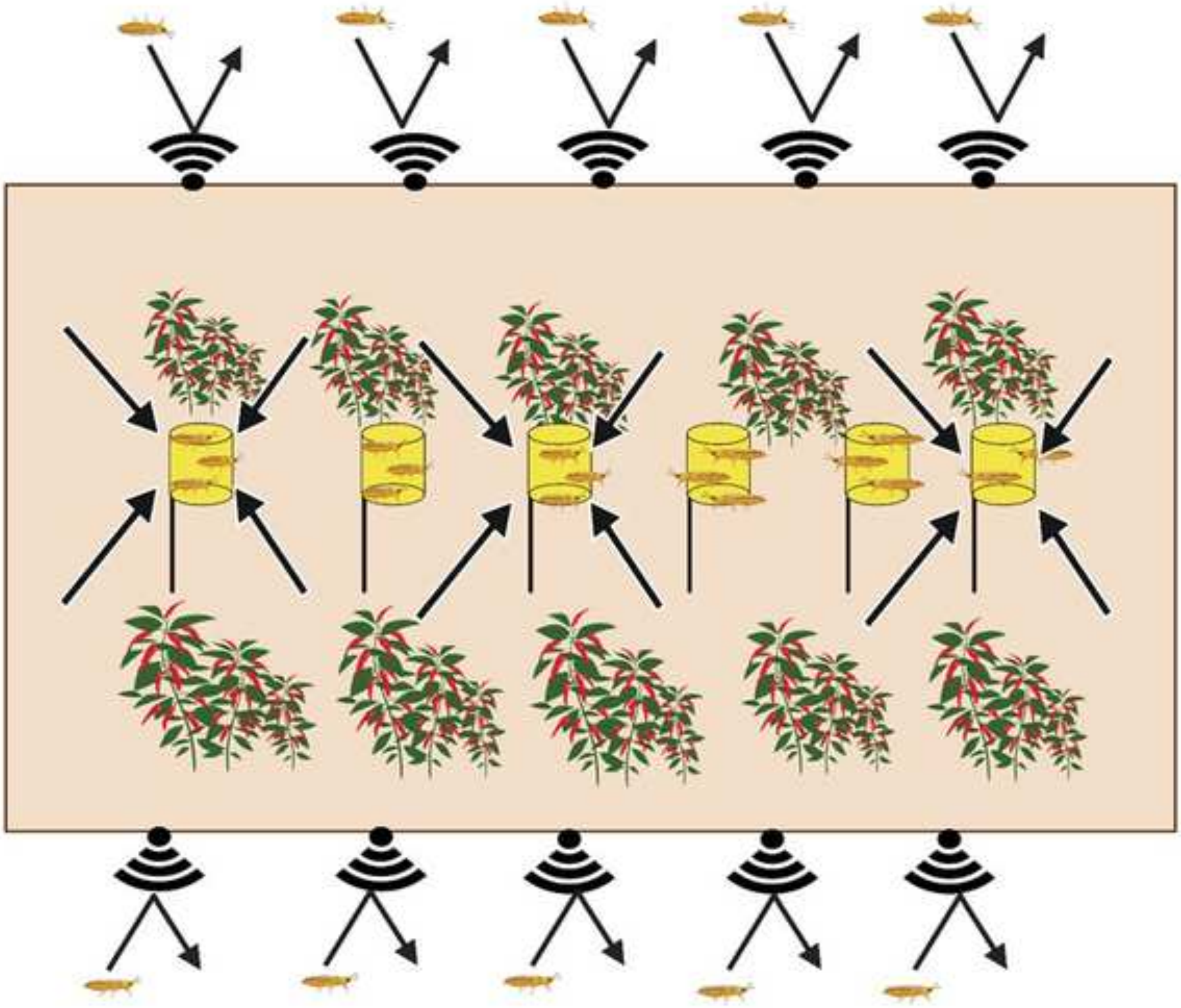

Click here to access/download
Supporting Information
Paper (PLoS ONE)-SI.docx

PLoS ONE

A push-pull strategy to control the western flower thrips, *Frankliniella occidentalis*, using alarm and aggregation pheromones

Chul-Young Kim, Falguni Khan, and Yonggyun Kim

Department of Plant Medicals, College of Life Sciences, Andong National University, Andong 36729, Republic of Korea

Corresponding author: hosanna@anu.ac.kr

~~**Funding:** This work was conducted with the support of the Cooperative Research Program for Agriculture Science & Technology Development (Project No. PJ01578901) funded by the Rural Development Administration, Republic of Korea.~~

Abstract

Since the first report in 1993 in Korea, the western flower thrips, *Frankliniella occidentalis*, has been found in various crops throughout the country. Although more than 20 different chemical insecticides are registered to control this insect pest, its outbreaks seriously damage crop yields, especially in greenhouses. This study developed a non-chemical technique to control *F. occidentalis* infesting hot peppers cultivated in greenhouses. The method was based on behavioral control using an alarm pheromone ("Push") to prevent the entry of the thrips into greenhouses and an aggregation pheromone ("Pull") for mass trapping inside the greenhouses. The greenhouse fences were treated with a wax formulation of the alarm pheromone and a yellow CAN trap covered with sticky material containing the aggregation pheromone was constructed and deployed inside the greenhouses. Field assay demonstrated the efficacy of the push-pull tactics by reducing thrips density in flowers of the hot peppers as well as in the monitoring traps. Especially, the enhanced mass trapping to the CAN trap compared to the conventional yellow sticky trap led to significant reduction in the thrips population. This novel push-pull technique would be applicable to effectively control *F. occidentalis* in field conditions.

Keywords: *Frankliniella occidentalis*, aggregation pheromone, alarm pheromone, push-pull strategy, hot pepper

1. Introduction

The western flower thrips, *Frankliniella occidentalis* (Pergande) (Thysanoptera: Thripidae), is one of the most devastating insect pests to many horticultural crops including more than 500 species spanning 50 plant families, especially those cultured in greenhouses [1-3]. Both the larval and adult stages cause damage to plants by directly feeding on leaves or flowers [4,5]. Especially, adults transmit plant viruses including tomato spotted wilt virus (TSWV) [6,7]. TSWV infections have become serious in several horticultural products in Korea, including the high-value hot pepper crop [8]. This pest, originally native to North America, has spread to more than 60 countries since the late 1970s, including Europe, Asia, and Australia [9]. It was first observed on Jeju Island in 1993 and now has been spread to most regions in Korea [10].

Different reproductive modes along with a short lifecycle allow the thrips population to rapidly increase during early and late seasons, in which female progeny develop from fertilized eggs while males develop from unfertilized eggs [11,12]. The nutrient-based oogenesis of *F. occidentalis* is well-programmed in females by gonadotropic hormones including juvenile hormone, ecdysone, and prostaglandin [13,14]. To suppress thrips outbreaks, more than 20 kinds of chemical insecticides including organophosphate, carbamate, pyrethroids, neonicotinoids, avermectin, and spinosad are registered and applied to different agricultural crops [15]. However, the control efficacy against *F. occidentalis* is often unsatisfactory due to its cryptic ability to avoid exposure to the chemical spray, and the development of insecticide resistance [16]. A resistant population to emamectin benzoate showed a 356-fold increased resistance by a specific mutation in the target site and an over-expression of a detoxifying enzyme compared to a susceptible strain in Korea [17]. As alternative control tactics, the use of natural enemies, microbial pathogens, and other biological control agents has been proposed

under an integrated insect pest management (IPM) program against *F. occidentalis* [18]. In this IPM program, behavioral control using pheromones has been considered in the monitoring and mass trapping of thrips [19].

Chemical communication using pheromones plays a crucial role in mediating *F. occidentalis* populations [9]. A contact pheromone, 7-methyltricosane produced by male adults, acts as a territorial signal by eliciting a fight signal to other males or gives the location of a male to females [20]. Kirk and Hamilton [21] suggested the presence of male-producing sex pheromones attracting only females. The aggregation pheromone of *F. occidentalis* was identified as containing two components, neryl methylbutanoate (NMB) and lavandulyl acetate (LA) [22]. Kirk et al. [23] suggested another type of chemical communication in mating, an anti-aphrodisiac pheromone produced by males during mating to prevent the multiple mating of females [24]. *F. occidentalis* larvae release an alarm pheromone in an anal droplet containing decyl acetate (10:Ac) and dodecyl acetate (12:Ac), in which the two components are present in a relative mass ratio of 0.4:1 to 1.1:1, respectively [25]. This alarm signal promotes the vigilance of thrips as well as takeoff or refuge-seeking behaviors to survive predator attacks [26]. Moreover, the relative ratio of the two components and their total amounts vary with the hazard intensity by increasing the relative amount of 12:Ac or the total alarm pheromone concentration in the droplet, suggesting that the alarm signal is context-dependent [27].

Using aggregation and alarm pheromones, this study devised a push-pull control strategy against *F. occidentalis* in greenhouse conditions. Fence treatment with alarm pheromone was used to prevent the entry of thrips into the greenhouse while an aggregation pheromone was used for mass trapping inside the greenhouse. To investigate this control strategy, we tested the active pheromone compounds and determined their blend ratio in the laboratory. For field assays, both pheromones were formulated for sustainability in field conditions. Finally, the

push-pull tactic was applied to a specific farm without chemical sprays and the control efficacy was compared to that of farms that followed a conventional insecticide spraying program for thrips.

2. Materials and methods

2.1. Thrips rearing

A laboratory colony of *F. occidentalis* that originated from a local population in Jeonju in Korea was donated by the Rural Development Administration (Jeonju, Korea). It was maintained under 25 ± 1° C constant temperature and a 16:8 h (L:D) photoperiod with a relative humidity of 60 ± 5%. Another laboratory colony of *F. intonsa* was obtained from a field collection in a hot pepper field in Geosan (N36.81 E127.78, Korea) and reared in the laboratory conditions. All thrips were fed a kidney bean (*Phaseolus coccineus* L.) diet according to the method described in our earlier study [12].

2.2. Pheromones

Lavandulyl acetate (LA), lavandulyl methylbutanoate (LMB), and neryl methylbutanoate (NMB) were synthesized (AD, Inc., Andong, Korea) (S1 Fig). Briefly, LA and LMB were synthesized from the precursor, lavandulol (≥ 90%, Sigma-Aldrich Korea, Seoul, Korea) in dichloromethane, which was reacted with acetyl chloride to yield LA, and reacted with isovaleryl chloride (≥ 98%, Sigma-Aldrich Korea, Seoul, Korea) to yield LMB, respectively, under the catalytic activity of trimethylamine. NMB was used in a reaction with geraniol (>

98%, Sigma-Aldrich Korea) and isovaleryl chloride under the catalytic activity of trimethylamine. All products were extracted with ethyl acetate and produced 70 ~ 75% yields. The purity of the compounds was analyzed by gas chromatography (8860 GC, Agilent, Santa Clara, CA, USA) with a DB-1 column (15 m × 0.350 mm, Agilent), an oven temperature of 280℃, and a flow rate of 1.0 mL/min, and was determined to be 98.1% for LA, 98.1% for LMB, and 99.3% for NMB. All synthesized products were in the form of a racemic mixture. Two alarm pheromone components (decyl acetate and dodecyl acetate, 98%) were purchased from Sigma-Aldrich Korea and dissolved in hexane to prepare different concentrations.

2.3. Construction of CAN trap

The CAN trap consisted of a short supporting bar (1 m), an empty can (diameter 17 cm and height 18.5 cm), and a sticky plastic bag (see Fig 2). The short supporting bar and the empty can were connected with nails and attached to another long supporting bar (1 m) at an appropriate position for the trapping height. To mix the pheromone with sticky material, 2 mg of pheromone components and 12 g of sticky material (Tanglefoot, Contech Electronics, Rochester, NY, USA) were dissolved in 6 mL of hexane. This mixture was then poured to a sticky plastic zipper bag (30 × 30 cm, HG Pack, Gunpo, Korea). After spreading the sticky mixture with a roller, the bag was used to cover the can by inside-out inversion.

2.4. Wax formulation of alarm pheromone

The alarm pheromone (a mixture of decyl acetate and dodecyl acetate in 1.5:1, g/g) was

formulated in wax by the method of Kim et al. [28]. Briefly, paraffin wax (Merck, Rahway, NJ, USA) was dissolved and mixed with an emulsifier (Almax 3600, Illshinwells, Seoul, Korea), α-tocopherol (96%), and jojoba oil (see Fig 5A). After the alarm pheromone was added, the slurry was cooled to solidify.

2.5. Aggregation behavior test using Y-tube olfactometer

A Y-type olfactometer (the main Y-tube length, 5 cm; two branches 2 cm long; a 45° angle between the branches; inner branch diameter, 5 cm) was placed in a dark room at 25 ± 1°C to avoid visual cue for the choice test. A flow of clean (charcoal-filtered) air at a rate of 0.6 L/min was split and passed through two glass vessels containing either an odor source or control (hexane) stimuli and entered each of the branches of the Y-tube. An air supply system (Power Air Pump, Seoul, Korea) was used for air filtration and flow rate control. Before and after the bioassay, the Y-tube was cleaned with 100% methanol. Test thrips were used three days after adult emergence. Each test used 10 ~ 30 individuals of each sex and was replicated four times by changing the source and control for replication. The response was positive when the thrips passed through the midpoint of the branches for 10 min. Otherwise, the thrips' response was recorded as "no response."

2.6. Alarm pheromone test using dispersal behavior assay

An arena was designed using a Petri dish (10 × 3 cm, SPL Life Science, Seoul, Korea) to test the dispersion behavior of the thrips. Test thrips (20 individuals per replication) on the bean diet were put into the center of the dish. A disc (3-mm diameter filter paper) was put near the

diet and 10 μL of test solution in hexane was added. After 1 min, the thrips dispersed away from the bean were counted. Hexane was used as the control. Each treatment was replicated three times.

2.7. Field test for alarm pheromone

A greenhouse (220 m^2) cultivating hot peppers was used in this test. *F. occidentalis* was heavily infesting the peppers. In the middle of the greenhouse, test hot peppers in pots (one plant per pot) without any thrips were randomly assigned by a randomized block design with three replications. Each test plant was covered with a plastic cage (45 × 45 × 45 cm), which had large openings in five directions to allow the entry of nearby thrips. The wax-formulated pheromone (5 g) was loaded in a small pheromone cage (Green Agrotech, Kyungsan, Korea), which was hung at the opening gates of the cage. An empty pheromone cage was used as the control.

The duration of alarm pheromone efficacy was tested by harvesting the lure in the field conditions once a week. The efficacy test was conducted using the dispersion behavior assay described above.

2.8. Field test for push-pull tactics

A test greenhouse (660 m^2) cultivating about 200 hot peppers was located in Gudam (GD, N36.54 E128.46), Andong, Korea. Alarm pheromones in the wax formation were applied by pasting each (5 g) to the fence around the greenhouse at 2-m intervals for pushing the thrips immigrating from outside. The alarm pheromones were re-applied every four weeks. Twenty

CAN traps containing aggregation pheromone were installed inside the greenhouse for pulling the thrips. The sticky bags were replaced every week. During the assay, no chemical insecticides were applied. For reference, thrips presence was compared in two other fields cultivating hot peppers. One was a greenhouse in Hahoe (HH, about 4 km from GD) and an open field in Songchun (SC, 16 km from GD) (Fig. 6A). Both fields were frequently treated with chemical insecticides including spinosad during the assay.

2.9. Thrips monitoring test in the field

Three yellow sticky traps (Green Agrotech) were installed inside the greenhouse for monitoring changes in the thrips population from March to July. Thrips were identified based on the morphological characters described in an earlier study [12]. Number of *F. occidentalis* in flowers was counted from 10 randomly selected flowers with three replications in each field. The monitoring was performed once a week for one month (June 13 ~ July 13). TSWV infection rates in the hot peppers were measured by ring-spot disease symptoms on leaves and fruits. Each experimental unit was 100 randomly chosen hosts and was replicated three times. To determine TSWV-infected thrips, multiplex polymerase chain reaction (PCR) analysis (see below) was used to discriminate viruliferous thrips. In each site/collection, 30 thrips were used to analyze the viruliferous thrips for the period.

2.10. Molecular diagnosis of TSWV using multiplex PCR

A single insect was homogenized in 35 μL of an RNA extraction solution (LGC Bioresearch Technologies, Hoddesdon, UK) in a 1.5-ml sample tube. The homogenate was incubated at 95 ℃

for 5 min and centrifuged at 13,500 × *g* for 5 min to obtain supernatant. To extract RNA from hot peppers, a piece of a leaf (1 g) was homogenized in 250 μL of a lysis buffer in the Viral DNA/RNA Extraction Kit (Intron Biotechnology, Sungnam, Korea) and RNA was extracted according to the manufacturer's instruction. PCR amplification was performed in a total volume of 20 μL containing 4 μL of template RNA, 8 μL of primers, and 8 μL of SuPrime Script RT-PCR Premix (2X) (Genetbio, Daejeon, Korea). Seven primers [12] were designed for the TSWV (5'-TGTCTAAGGTTAAGCTCACTAAGGAA-3' and 5'-TTAAGCAAGTTCTGCAAGTATTGCCTG-3'), *F. occidentalis* (forward primer, 5'-GGT CGC TTC ACC GCT TCC CG-3'), *F. intonsa* (forward primer, 5'-GAC CAG ACT GTT CCG AGA-3'), a common reverse primer (5'-CTC TCC TGA ACW GAG GCT G-3'), and *T. tabaci* (5'-TCT AAA CAG AGG GAA AGG TG-3' and 5'-AGT GTG CCA ACA AGG CAA TG-3'). Reverse transcription was performed at 50°C for 30 min. After a denaturation step at 95 °C for 5 min, the subsequent PCR was proceeded according to the following temperature cycle program: 35 cycles of 95°C for 30 s, 55°C for 30 s, and 72°C for 1 min. The PCR products were electrophoresed on 2% (w/v) agarose gel containing 1 × TAE buffer and were visualized by UV light on a Gel Doc imaging system (Bio-Rad, Hercules, CA, USA). The resulting PCR product sizes were 777 bp for TSWV, 287 bp for *F. occidentalis*, 367 bp for *F. intonsa*, and 417 bp for *T. tabaci* [12].

2.11. Statistical analysis

Percentage data were arcsine-transformed and confirmed in normality using PROC UNIVARIATE in the SAS program [29]. Data obtained from the Y-tube or choice test were subjected to a one-way analysis of variance (ANOVA) using PROC GLM. Field assay data

were assessed by two-way ANOVA. The means were compared using the least significant difference (LSD) test at a Type I error of 0.05. Frequency data were analyzed by the mean difference test using PROC FREQ.

3. Results

3.1. Optimal pheromone composition to attract thrips

The ability of two components (LA and NMB) of the aggregation pheromone to attract thrips was tested using two thrips, *F. occidentalis* and *F. intonsa* (Fig 1). In the behavioral assays, methylbutanoate lavandulol (LMB) was used as the control (Fig 1A). LA and NMB were significantly (F = 73.50; df = 1, 6; P = 0.0001 for LA; F = 19.11; df = 1, 6; P = 0.0047 for NMB) effective in attracting adult female *F. occidentalis* and male *F. intonsa*. However, LMB did not significantly attract thrips. Then, we prepared blends containing different ratios (1:2, 1:4, and 1:7, g/g) of LA and NMB and compared their attractiveness (Fig 1B). Except for the 1:2 ratio blend, the other two blends effectively attracted both thrips species. Even though males did not show significant preferences, *F. occidentalis* females were significantly (F = 96.43; df = 1, 6; P < 0.0001) attracted to the 1:7 blend whereas *F. intonsa* females were significantly (F = 27.77; df = 1, 6; P = 0.0019) attracted to the 1:4 blend (Fig 1C).

3.2. Development of the yellow CAN trap containing aggregation pheromone

A yellow sticky trap (PLATE-type) has been used to monitor thrips. To enhance the trapping efficiency, a CAN-type trap covering an empty can with a sticky zipper bag (CAN-type) was

designed to attract thrips from all directions because the PLATE-type attracted only to the front and back sides. When the attractiveness of the PLATE-type trap was compared to that of the CAN-type trap without any aggregation pheromone treatment, they were not significantly different based on the number of thrips per sticky unit area (Fig 2A). However, the trap color significantly (F = 5.63; df = 4, 10; P = 0.0123) influenced thrips trapping, in which attractiveness was nearly lost in black traps (Fig 2B). The addition of the aggregation pheromone to the CAN trap significantly (F = 11.34; df = 1, 4; P = 0.0028) enhanced attractiveness even in a black background (Fig 2C). With a yellow background, the addition of the aggregation pheromone increased the attractiveness of the CAN trap by almost five-fold compared to the trap without the pheromone. Based on the findings, a yellow CAN trap was constructed (Fig 2D). For the CAN trap, LA (0.25 mg) and NMB (1.75 mg) were mixed with a sticky material (12 g of sticky material in 6 mL of hexane). The mixture was then poured into a zipper bag and spread using a roller. When the sticky bag was applied to the CAN trap, the sticky inside was inverted to the outside. The height of the CAN trap was controlled by the length of the supporting bar (Fig 2E).

3.3. Trapping efficiency of CAN trap in field conditions

The trapping efficiency of the CAN trap for *F. occidentalis* in field conditions was compared to that of a yellow sticky plate (PLATE), which is currently used to monitor thrips including *F. occidentalis* (Fig 3). When we compared the trapping efficiency of these two traps for a week (June 8 ~ June 15), the CAN trap showed > 4-times higher efficiency than the PLATE-type (Fig 3A). Then, we analyzed the number of *F. occidentalis* collected by the two traps over five successive weeks (Fig 3B). The collection numbers were normalized by sticky

unit area because of their different sizes. Except for one week in which thrips occurred at a low density, the CAN trap was much more attractive than the PLATE trap (t = 5.29; df = 24; P < 0.0001).

Unexpectedly, the CAN trap was also highly efficient (t = 9.99; df = 24; P < 0.0001) in attracting the whiteflies of *B. tabaci* compared to the PLATE-type. Thrips collected by the CAN trap were classified into different species and the composition was compared to that of the thrips collected by the PLATE trap (Fig 3C). The CAN trap containing the *F. occidentalis* aggregation pheromone attracted all three thrips species of *F. occidentalis*, *F. intonsa*, and *T. tabaci* but it attracted more *F. occidentalis* than the PLATE trap without pheromone (X^2 = 14.03; df = 2; P = 0.0009).

3.4. Development of thrips repellent using alarm pheromone

In response to the alarm pheromone (10:Ac and 12:Ac = 1: 1.5, g/g), larvae and adults exhibited dispersal behavior within a few seconds (Fig 4A). This dispersal behavior was also observed in response to individual components, in which 12:Ac induced the behavior in both stages, whereas 10:Ac was effective only in adults (Fig 4B). Dispersal behavior to the alarm pheromone components was highly induced in adults compared to larvae. The two-component blend was more effective in inducing the dispersal behavior than single component treatments except for the comparison of 12:Ac and the blend in the adult stage. To clarify the effectiveness between the single-component (12:Ac) and the two-component blend, different doses were applied and the efficacy of inducing dispersal behavior was assessed (Fig 4C). The two-component blend was much more effective at low doses compared to the single component in both larvae and adults. In addition, the two-component blend was effective in inducing the

dispersal behavior of *F. intonsa* (Fig 4D). However, the dispersal behavior was highly induced in *F. occidentalis* compared to *F. intonsa* at low doses. These results indicate that the alarm pheromone induced the dispersal behavior of thrips in both larvae and adults and acted as a repellent because the thrips fled the pheromone source.

The alarm pheromone-based repellent was tested in field conditions. The repellent was formulated with wax for applying by pasting (Fig 5A). To test the efficacy of the formulation, the repellents were applied to hot peppers infested with *F. occidentalis* in a greenhouse (Fig 5B). Test plants without any thrips were covered with a plastic cage, which had large openings in four directions to allow the entry of nearby thrips. The repellents were then applied to the openings. In the control without the repellent, thrips arrived within a day and maintained an immigrating population. However, hot peppers treated with the repellents maintained significantly (F = 192.79; df = 1, 34; $P < 0.0001$) low levels of thrips for at least two weeks (Fig 5C). The repellent efficacy of the wax formulation was maintained for four weeks without significant (F = 1600.00, df = 4, 10; P = 0.0001) differences in the greenhouse conditions.

3.4. Application of the push-pull strategy to an agricultural farm cultivating hot peppers

The occurrence of *F. occidentalis* in three different greenhouses cultivating hot peppers in Andong was compared (Fig 6A). Push-pull control was applied to the GD greenhouse (Fig 6B), while HH and SC were negative controls and heavily treated with conventional insecticides, in which HH hot peppers were in a greenhouse, but SC peppers were in an open field. *F. occidentalis* adults usually occur in greenhouses in late March in Andong [12]. Our current study also found that the first adults were caught in greenhouses on March 23 in HH and April 27 in GD. In contrast, the occurrence of adults was delayed and first detected in early May in

an SC open field. After small peaks in April-May, the main adult peak was observed in June-July in all three field conditions. In the main peak, HH had many more adults than GD by more than 1,000-fold. The occurrence of *F. occidentalis* adults in GD was lower than that in the SC open field. *F. occidentalis* usually occurs at low levels because the thrips prefer greenhouse conditions over open fields [30]. To further analyze the effect of the CAN trap, thrips populations in the flowers were compared (Fig 6C). The number of *F. occidentalis* thrips in the flowers was significantly lower in CAN trap-treated GD compared to the control farms (F = 5.89; df = 10, 30; $P < 0.0001$). *F. occidentalis* is a vector for TSWV. The TSWV infection rate was significantly different between farms (F = 8.55; df = 2, 22; P = 0.0049). Indeed, no hot peppers were infected with TSWV in CAN trap-treated GD (Fig 6D). However, the control farms had TSWV-infected plants, in which the SC site showed an infection rate of more than 30%. This was further supported by the multiplex PCR analysis. No *F. occidentalis* was infected with TSWV, whereas significant numbers of *F. occidentalis* adults were positive for TSWV in HH and SC.

4. Discussion

This study proposes a push-pull control strategy using alarm and aggregation pheromones against *F. occidentalis* in greenhouse conditions. To test these novel control tactics, the push-pull effects of the chemical compounds were re-evaluated. For field assays, both pheromones were formulated as wax types for the alarm pheromone and a CAN trap for the aggregation pheromone. These formulated pheromones were applied to a practical farm and the control efficacy was compared to those of other conventional farms treated with chemical insecticides. Our laboratory and field data supported the control efficacy of push-pull control tactics against

F. occidentalis.

To enhance the attractiveness for *F. occidentalis*, two aggregation pheromone components (LA and NMB) were re-evaluated with an analog, lavandulyl methylbutanoate (LMB). LMB was identified as an aggregation pheromone component of another flower thrips, *Megalurothrips sjostedti* [31], and onion thrips, *Thrips palmi* [32]. No significant attractiveness of LMB for either *F. occidentalis* or *F. intonsa* was seen by Y-tube olfactometry. However, they were attracted to LA and NMB. The aggregation pheromones LA and NMB are shared between *F. occidentalis* and *F. intonsa*, so species-specific blend mixtures were used to prevent cross-attraction [33]. In our assay, *F. occidentalis* preferred a 1:7 (LA:NMB) ratio to a 1:4 ratio, whereas *F. intonsa* exhibited an opposite preference. Interestingly, a significant preference was observed only in the females of both species in the Y-tube olfactometer analysis in the double-choice experiment between 1:4 and 1:7 ratios, although both sexes were attracted to both ratios in the single-choice experiments. Considering that the aggregation pheromone is basically attractive to both sexes, our assay might not detect a male preference. The lower sensitivity of males may be their preference for a specific stereoisomer because our synthetic pheromone components (S1 Fig) were mixtures of stereoisomers. The LA:NMB ratio in *F. occidentalis* was reported to be within a range of 1:0.8 ~ 1:5 measured by solid-phase microextraction (SPME) [22]. In that study, the addition of LA to NMB gave no increase relative to NMB alone, leading to the development of a single-component commercial product. In our current study, however, the inclusion of LA in the aggregation pheromone may have had some physiological significance because unlike the 1:4 and 1:7 ratios, the 1:2 mixture ratio did not significantly attract *F. occidentalis*. This suggests the role of LA in the aggregation signal of *F. occidentalis*. Two congener species of *F. occidentalis* and *F. intonsa* share flower habitats and aggregation pheromone components, in which *F. occidentalis* had 1:13 (LA:NMB) ratio while *F. intonsa*

had 1:2 ratio in a Chinese population measured by SPME [34]. The significance of these different effective mixture ratios was confirmed in field experiments where a 1:8 ratio of aggregation pheromone components caught more *F. occidentalis,* whereas a 1:4 ratio attracted more *F. intonsa* [33]. Thus, the role of LA may give species-specificity to the aggregation pheromone. Furthermore, in our current assay, LA alone significantly attracted *F. occidentalis*. Thus, we determined the optimal mass trapping lure to be a mixture of LA and NMB at a 1:7 mixture ratio.

An alarm pheromone consisting of 10:Ac and 12:Ac induced significant dispersion behavior in *F. occidentalis*. It was reported that in response to the alarm pheromone, larvae exhibited escape behavior from the source by a rapid drop from host plants, whereas exposed adults exhibited take-off behavior and avoided landing in the area [35]. Indeed, the deployment of the alarm pheromone significantly prevented the invasion of the thrips to hot peppers in the current study. These facts led us to apply the alarm pheromone to the push-pull strategy to control *F. occidentalis*. Other potent repellents may be developed from other sources. For example, plant odors emitted by non-preferred hosts, such as garlic (*Allium sativum*) and celery (*Apium graveolens*), showed repelling effects on *F. occidentalis* females [36].

To apply the pheromones to field conditions, they were formulated to meet the purpose of the push-pull strategy. Alarm pheromone components were formulated using wax for fence treatment by pasting. The wax formulation modified the methods used in other lure formulations developed using moth sex pheromone and fly lure, where their efficacy was confirmed in field conditions [28,37]. The bioactivity of the wax formulation of the alarm pheromone lasted for at least four weeks in greenhouse conditions. Aggregation pheromone was formulated by mixing with sticky material in an attract-to-kill strategy. For this purpose, a CAN trap was devised and effectively attracted thrips by its yellow color and aggregation

pheromone. The question has been raised of whether thrips from nearby plants or thrips flying over plants were collected by the CAN traps. The study findings present indirect evidence that CAN traps collected thrips flying over the plants because adult thrips in the hot pepper flowers were not evenly distributed, whereas the 20 installed CAN traps collected similar quantities of thrips. This suggests that our CAN traps attracted flying thrips. A synergistic effect of the CAN trap and another biological control method using a natural enemy was also suggested because it is known that the predator *Orius laevigatus* stimulates the release of an alarm pheromone [27], which induces take-off flight in flying thrips in host plants.

Interestingly, the trapping efficiency of the yellow CAN trap for the whitefly *B. tabaci* was enhanced without an aggregation pheromone. This suggests that the aggregation pheromone of *F. occidentalis* was effective in attracting *B. tabaci*. The two aggregation pheromone components are terpenoids. Whiteflies respond to various terpenoids including sesquiterpenes (zingiberene and curcumene) and monoterperpenes (*p*-cymene, *α*-terpinene, and *α*-phellandrene), although terpenoids act as repellents [38]. It is noteworthy that LMB, one of the aggregation pheromone components in thrips, is used by a sex pheromone of the mealybug, *Phenacoccus madeirensis* [39]. Further studies assessing the sensitivity and response of *B. tabaci* to the aggregation pheromone of *F. occidentalis* need to be conducted.

The push-pull strategy was effective in suppressing the frequency of TSWV-infected hot peppers. Compared to other fields such as those in HH and SC, the thrips density was low in flowers in GD treated with the push-pull strategy, as was the number in monitoring traps. TSWV-infected hot peppers exhibit characteristic disease symptoms of ring-spots on leaves and fruit [40]. Based on this disease symptom, the survey indicated no TSWV infections in hot peppers in GD in contrast to the significant numbers of disease symptoms in the other two fields. This was further supported by the multiplex PCR results, which indicated no TSWV-

infected *F. occidentalis* in GD compared to large numbers of TSWV-positive thrips in other fields. This might be explained by a local variation in viruliferous thrips as similar variations in *Thrips tabaci* carrying TSWV were seen [41]. Alternatively, the suppression of thrips occurrence by the push-pull treatment might have caused the decrease in the TSWV infection rate.

The push-pull strategy has been used for effective insect pest control [42]. A push-pull control technique against *F. occidentalis* was proposed using UV-reflective mulch as a pushing factor to disrupt thrips colonization on host plants along with a companion plant as a pulling factor to attract the predators and trap the thrips [43]. Alternatively, the use of semiochemicals such as attractants and repellents has been considered for developing a push-pull strategy [44]. Kirk et al. [23] proposed *F. occidentalis* control using pheromones and other semiochemicals. This study demonstrated novel push-pull tactics using aggregation/alarm pheromones (Fig 7) to control *F. occidentalis* in greenhouse conditions. This control technique effectively suppressed the outbreak of *F. occidentalis* in a field assay and also prevented the occurrence of plant disease caused by TSWV in hot peppers. Although our current study directly evaluated the pulling efficacy of the attracting CAN trap, the pushing efficacy of the repellent fence treatment was indirectly predicted from a small-scale greenhouse experiment. Subsequent studies are needed to assess thrips occurrence outside of a test greenhouse treated with the alarm pheromone to improve the estimation of the pushing efficacy in field conditions.

Supplementary information

S1 Fig. Chemical synthesis of the aggregation pheromone components of thrips: lavandulyl

acetate (LA, 2), lavandulyl methylbutanoate (LMB, 3), and neryl methylbutanoate (NMB, 5). LA and LMB were synthesized from the precursor, lavandulol (1) in dichloromethane, which was reacted with acetyl chloride to yield LA, and was reacted with isovaleryl chloride to yield LMB, respectively, under the catalytic activity of trimethylamine. NMB was reacted with geraniol (‘4’) and isovaleryl chloride under a catalytic activity of trimethylamine. The purity of the compounds was analyzed by gas chromatography (8860 GC, Agilent, Santa Clara, CA, USA) with a DB-1 column (15 m × 0.350 mm, Agilent) at an oven temperature of 280 ℃ and a flow rate of 1.0 mL/min.

S2 Fig. Tests of two flower thrips (*F. occidentalis* (‘Fo’) and *F. intonsa* (‘Fi’)) to aggregation pheromone components using a Y-tube olfactometer. The test pheromone components included LA (lavandulyl acetate), LMB (lavandulyl methylbutanoate), and NMB (neryl methylbutanoate). Each response (experimental unit) used 10 adults (< 3-days-old after emergence). Each treatment was replicated four times.

Acknowledgements

We thank Jean Lee and Juan Hong in our laboratory to provide thrips for our analysis.

~~Funding Statement~~

~~The funders had no role in study design, data collection and analysis, decision to publish, or preparation of the manuscript.~~

Author Contributions

Conceptualization: Yonggyun Kim.

Formal analysis: Chul-Young Kim, Falguni Khan, Yonggyun Kim.

Funding acquisition: Yonggyun Kim.

Investigation: Chul-Young Kim, Falguni Khan, Yonggyun Kim.

Methodology: Chul-Young Kim, Falguni Khan, Yonggyun Kim.

Project administration: Yonggyun Kim.

Validation: Chul-Young Kim.

Writing – original draft: Chul-Young Kim, Yonggyun Kim.

Writing – review & editing: Yonggyun Kim.

References

1. Yudin LS, Cho JJ, Mitchell WC. Host range of western flower thrips, *Frankliniella occidentalis* (Thysanoptera: Thripidae), with special reference to *Leucaena glauca*. Environ Entomol. 1986; 15:1292-1295.
2. Schneweis DJ, Whitfield AE, Rotenberg D. Thrips developmental stage-specific transcriptome response to tomato spotted wilt virus during the virus infection cycle in *Frankliniella occidentalis*, the primary vector. Virology. 2017; 500:226–237.
3. He Z, Guo JF, Reitz SR, Lei ZR, Wu SY. A global invasion by the thrips, *Frankliniella occidentalis*: current virus vector status and its management. Insect Sci. 2020; 27:626-645.
4. Reitz SR. Biology and ecology of the western flower thrips (Thysanoptera: Thripidae): The making of a pest. Fla Entomol. 2009; 92:7-13.

5. Shrestha A, Srinivasan R, Riley DG, Culbreath AK. Direct and indirect effects of a thrips-transmitted Tospovirus on the preference and fitness of its vector, *Frankliniella fusca*. Entomol Exp Appl. 2012; 145:260-271.
6. Belliure B, Janssen A, Maris PC, Peters D, Sabelis MW. Herbivore arthropods benefit from vectoring plant viruses. Ecol Lett. 2005; 8:70-79.
7. Rotenberg D, Jacobson AL, Schneweis DJ, Whitfield AE. Thrips transmission of tospoviruses. Curr Opin Virol. 2015; 15:80-89.
8. Lee S, Lee J, Kim S, Choi H, Park J, Lee J, et al. The incidence and distribution of viral diseases in pepper by cultivation types. Res Plant Dis. 2004; 10:231-240.
9. Reitz SR, Gao Y, Kirk WDJ, Hoddle MS, Leiss KA, Funderburk JE. Invasion biology, ecology, and management of western flower thrips. Annu Rev Entomol. 2020; 65:17-37.
10. Woo KS, Ahn SB, Lee SH, Kwon HM. First record of *Frankliniella occidentalis* and its distribution and host plants in Korea. Korean J Appl Entomol. 1994; 33:127.
11. Ding T, Chi H, Gökçe A, Gao Y, Zhang B. Demographic analysis of arrhenotokous parthenogenesis and bisexual reproduction of *Frankliniella occidentalis* (Pergande) (Thysanoptera: Thripidae). Sci Rep. 2018; 8:3346.
12. Kim C, Choi D, Kang J, Ahmed SA, Kil E, Kwon G, et al. Thrips infesting hot pepper cultured in greenhouses and variation in gene sequences encoded in TSWV. Korean J Appl Entomol. 2021; 60:387-401.
13. Choi DY, Kim Y. PGE_2 mediation of egg development in western flower thrips, *Frankliniella occidentalis*. Arch Insect Biochem Physiol. 2022; e21949.
14. Choi DY, Kim Y. Transcriptome analysis of female western flower thrips, *Frankliniella occidentalis*, exhibiting neo-panoistic ovarian development. PLoS ONE 2022; 17:e0272399.

15. Cho S, Kyung Y, Cho S, Shin S, Jeong D, Kim S, et al. Evaluation of susceptibility of western flower thrips (*Frankliniella occidentalis*) and garden thrips (*F. intonsa*) to 51 insecticides. Korean J Appl Entomol. 2018; 57:221-231.

16. Bhuyain MMH, Lim UT. Relative susceptibility to pesticides and environmental conditions of *Frankliniella intonsa* and *F. occidentalis* (Thysanoptera: Thripidae), an underlying reason for their asymmetrical occurrence. PLoS ONE 2020; 15:e0237876.

17. Gao Y, Yoon KA, Lee JH, Kim JH, Lee SH. Overexpression of glutamate-gated chloride channel in the integument is mainly responsible for emamectin benzoate resistance in the western flower thrips *Frankliniella occidentalis*. Pest Manag. Sci. 2022; doi: 10.1002/ps.7032.

18. Mouden S, Sarmiento KF, Klinkhamer PG, Leiss KA. Integrated pest management in western flower thrips: past, present and future. Pest Manag Sci. 2017; 73:813-822.

19. Sampson C, Kirk WD. Can mass trapping reduce thrips damage and is it economically viable? Management of the Western flower thrips in strawberry. PLoS ONE 2013; 8:e80787.

20. Olaniran OA, Sudhakar AV, Drijfhout FP, Dublon IA, Hall DR, Hamilton JG, et al. A male-predominant cuticular hydrocarbon, 7-methyltricosane, is used as a contact pheromone in the western flower thrips *Frankliniella occidentalis*. J Chem Ecol. 2013; 39:559-568.

21. Kirk WD, Hamilton JG. Evidence for a male-produced sex pheromone in the western flower thrips *Frankliniella occidentalis*. J Chem Ecol. 2004; 30:167-174.

22. Hamilton JG, Hall DR, Kirk WD. Identification of a male-produced aggregation pheromone in the western flower thrips *Frankliniella occidentalis*. J Chem Ecol. 2005; 31:1369-1379.

23. Kirk WDJ, de Kogel WJ, Koschier EH, Teulon DAJ. Semiochemicals for thrips and their use in pest management. Annu Rev Entomol. 2021; 66:101-119.

24. Akinyemi AO, Kirk WDJ. Experienced males recognise and avoid mating with non-virgin females in the western flower thrips. PLoS ONE 2019; 14:e0224115.

25. MacDonald KM, Hamilton JG, Jacobson R, Kirk WD. Analysis of anal droplets of the western flower thrips *Frankliniella occidentalis*. J Chem Ecol. 2003; 29:2385-2389.

26. de Bruijn PJ, Egas M, Janssen A, Sabelis MW. Pheromone-induced priming of a defensive response in Western flower thrips. J Chem Ecol. 2006; 32:1599-1603.

27. de Bruijn PJ, Egas M, Sabelis MW, Groot AT. Context-dependent alarm signalling in an insect. J Evol Biol. 2016; 29:665-671.

28. Kim K, Kim M, Kwon G, Kim Y. Technologies required for development of trap-based MAT control against the striped fruit fly, *Bactrocera scutellata*. Korean J. Appl. Entomol. 2017; 56:51-60.

29. SAS Institute Inc. SAS/STAT user’s guide, Release 6.03, Ed. Cary, NC. 1989.

30. Lee GS, Lee JH, Kang SH, Woo KS. Thrips species (Thysanoptera: Thripidae) in winter season and their vernal activities on Jeju island, Korea. J Asia Pac Entomol. 2001; 4:115–122.

31. Niassy S, Tamiru A, Hamilton JGC, Kirk WDJ, Mumm R, Sims C, et al. Characterization of male-produced aggregation pheromone of the bean flower thrips *Megalurothrips sjostedti* (Thysanoptera: Thripidae). J Chem Ecol. 2019; 45:348-355.

32. Akella SVS, Kirk WDJ, Lu YB, Murai T, Walters KFA, Hamilton JG. Identification of the aggregation pheromone of the melon thrips, *Thrips palmi*. PLoS ONE 2014; 9:e103315.

33. Li X, Geng S, Zhang Z, Zhang J, Li W, Huang J, et al. Species-specific aggregation pheromones contribute to coexistence in two closely related thrips species. Bull Entomol Res. 2019; 109:119-126.

34. Zhu XY, Zhang PJ, Lu YB. Isolation and identification of the aggregation pheromone released by male adults of *Frankliniella intonsa* (Thysanoptera: Thripidae). Acta Entomol Sin. 2012; 55:376-385.

35. Teerling CR, Pierce HD, Borden JH, Gillespie DR. Identification and bioactivity of alarm pheromone in the western flower thrips, *Frankliniella occidentalis*. 1993; 19:681-697.

36. Cao Y, Zhi J, Cong C, Margolies DC. Olfactory cues used in host selection by *Frankliniella occidentalis* (Thysanoptera: Thripidae) in relation to host suitability. J Insect Behav. 2014; 27:41-56.

37. Jung S, Park M, Lee S, Choi K, Hong Y, Bae S. et al. Development of wax-typed pheromone dispenser for mating disruption of the oriental fruit moth, *Grapholita molesta*, and its application technique. Korean J Appl Entomol. 2008; 47:255-263.

38. Bleeker PM, Diergaarde PJ, Ament K, Guerra J, Weidner M, Schütz S, et al. The role of specific tomato volatiles in tomato-whitefly interaction. Plant Physiol. 2009; 151:925-935.

39. Ho HY, Su YT, Ko CH, Tsai MY. Identification and synthesis of the sex pheromone of the Madeira mealybug, *Phenacoccus madeirensis* Green. J Chem Ecol. 2009; 35:724-732.

40. Lee JS, Cho WK, Kim MK, Kwak HR, Choi HS, Kim KH. Complete genome sequences of three tomato spotted wilt virus isolates from tomato and pepper plants in Korea and their phylogenetic relationship to other TSWV isolates. Arch Virol. 2011; 156:725-728.

41. Jacobson AL, Kennedy GG. Specific insect-virus interactions are responsible for variation in competency of different *Thrips tabaci* isolines to transmit different Tomato Spotted Wilt Virus isolates. PLoS ONE 2013; 8:e54567.

42. Cook SM, Khan ZR, Pickett JA. The use of push-pull strategies in integrated pest management. Annu Rev Entomol. 2007; 52:375-400.

43. Tyler-Julian K, Funderburk J, Srivastava M, Olson S, Adkins S. Evaluation of a push-pull system for the management of *Frankliniella* species (Thysanoptera: Thripidae) in tomato. Insects 2018; 9:187.

44. Niu Y, Han S, Wu Z, Pan C, Wang M, Tang Y. et al. A push-pull strategy for controlling the tea green leafhopper (*Empoasca flavescens* F.) using semiochemicals from *Tagetes erecta* and *Flemingia macrophylla*. Pest Manag Sci. 2022; 78:2161-2172.

Figure captions

Fig 1. **Differential preference analysis of two flower thrips (*F. occidentalis* and *F. intonsa*) for aggregation pheromone using a Y-tube olfactometer.** (A) Screening of active components and mixture ratios in the aggregation pheromone. The pheromone components were identified as LA (lavandulyl acetate), LMB (lavandulyl methylbutanoate), and NMB (neryl methylbutanoate). (B) Choice tests of the thrips against two pheromone blends. Each response (experimental unit) used 20 adults (< 3-days-old after emergence). Each treatment was replicated four times. The detailed data are presented in Figure S1.

Fig 2. **Development of a yellow CAN trap (CAN) to attract *F. occidentalis* using the aggregation pheromone.** (A) Comparison of the attractiveness of the CAN trap without aggregation pheromone and a yellow sticky trap (PLATE). (B) Effect of CAN trap color on attracting thrips. (C) Effect of aggregation pheromone on attracting thrips using the CAN trap, in which the black CAN trap demonstrated a pheromone effect while the yellow CAN trap demonstrated both pheromone and color effects. Pheromone components were prepared by mixing lavandulyl acetate (LA) and neryl methylbutanoate (NMB) at a 1:7 mass ratio. Each experimental unit represented a trap and was replicated three times. (D) Preparation of a sticky bag for the CAN trap. (E) Dimensions (mm) of the CAN trap parts and adjustment of the CAN trap height.

Fig 3. **Field efficacy of a yellow CAN trap containing aggregation pheromone.** (A) Enhanced trapping efficiency of the CAN trap containing aggregation pheromone compared to the yellow sticky plate (PLATE). CAN and PLATE traps used 20 and three replications,

respectively. An asterisk above the standard deviation bars indicates a significant difference between two treatments at type I error = 0.05 (LSD test). (B) Enhanced pulling effect of the CAN trap on trapping *F. occidentalis* and *B. tabaci* in different seasons. Two and three asterisks above the standard deviation bars indicate significant differences between two treatments at Type I error = 0.01 and type I error = 0.0001 (LSD test), respectively. NS indicates no significant difference. (C) Composition of thrips trapped by the PLATE and CAN traps. The average species frequencies in CAN and PLATE traps were obtained from 20 and three replications, respectively.

Fig 4. **Development of a thrips repellent using an alarm pheromone of *F. occidentalis*.** The alarm pheromone components were decyl acetate (10:Ac) and dodecyl acetate (12:Ac) at a 1:1.5 mass ratio. (A) Bioassay assessing repellent efficacy measured by the dispersal behavior of adults and larvae from bean diet after the test component (10 μL) was dispensed onto a filter paper disc. Arrows indicate the dispersal behavior of larvae. Different letters above the standard deviation bars indicate a significant difference between means at type I error = 0.05 (LSD test). (B) Repellent efficacy of single and dual components. (C) Dose-response of the pheromone components. (D) Comparison of dual component pheromone (alarm pheromone) efficacy in *F. occidentalis* (Fo) and *F. intonsa* (Fi). Each treatment was replicated three times. Asterisks above the standard deviation bars indicate significant difference between two treatments (LSD test): * at Type I error = 0.05, ** at Type I error = 0.01, and *** at Type I error = 0.0001. NS indicates no significant difference.

Fig 5. **Field assay of alarm pheromone-based repellent efficacy against *F. occidentalis* in a greenhouse cultivating hot peppers.** (A) Composition of the wax formulation of the

repellent and installation of the wax formulations (in a small cage) in five directions around a test plant. (B) Effect of the repellent on preventing immigrating thrips for two weeks. Three asterisks above the standard deviation bars indicate a significant difference between two treatments at Type I error = 0.0001 (LSD test). (C) Determination of the effective period of the wax formulation in field conditions. Different letters above the standard deviation bars indicate a significant difference among means at Type I error = 0.05 (LSD test).

Fig 6. **Application of push-pull strategy to *F. occidentalis* infesting hot peppers (200 plants) in a greenhouse (660 m^2).** (A) Deployment of repellents and CAN traps in a GD greenhouse. Adult occurrence in three sites. (B) Number of *F. occidentalis* in flowers. Each replication represents the total number of thrips from 10 randomly selected flowers. Each measurement was replicated three times. (C) TSWV infection rates in hot peppers and *F. occidentalis* over one month (June 13 to July 13). Each measurement represents the percentage of diseased plants out of 100 randomly chosen hosts. Each measurement was replicated three times. To determine TSWV-infected thrips, multiplex PCR analysis was performed to discriminate viruliferous thrips. In each site/collection, 30 thrips were used to analyze the viruliferous thrips for the period. An asterisk above the standard deviation bars indicates a significant difference among means at Type I error = 0.05 (LSD test). NS indicates no significant difference.

Fig 7. **A working model of the push-pull strategy against *F. occidentalis* using repellent (sound signal logo) and CAN trap (yellow can) in a greenhouse cultivating hot peppers.** Arrows indicate the push and pull moving directions.

Response to reviewers' comments

[Reviewer #1]

Comment #1-1: Introduction. I believe that you need a much strong justification for studying the western flower thrips, Frankliniella occidentalis as pest I did not find any strong justification for using F. occidentalis compared to other insects such as aphids for example!

Response: The thrips as a serious pest is explained by direct feeding damage and indirect TSWV transmission. We did not find the direct analysis on the statistical data of the relative damage compared to aphids. Thus we did not add the further information.

Comment #1-2: Materials and methods. The overall level of details for the Materials and Methods is appropriate, however there are some informations should be add in the text:

Line 98 page 5: Please precise if it was the same laboratory conditions described below.

Response: It is clarified as follows: "…. reared in the laboratory conditions."

Comment #1-3: Line 103-105 page 5: please give the purity of the molecules: Lavandulyl acetate (LA), lavandulyl methylbutanoate (LMB), and neryl methylbutanoate (NMB).

Response: Added

Comment #1-4: Line 108-110 page 6: Purity of molecules are missing here.

Response: Added

Comment #1-5: Line 114 page 6: add the purity of the alarm pheromone components

Response: added

Comment #1-6: Line 115: please correct Signal-Aldrich >> Sigma-Aldrich

Response: Corrected as suggested

Comment #1-7: Line 125: I did not find any justification for using hexane as solvent please explain.

Response: It is rephrased as follows: "To mix the pheromone with sticky material, 2 mg of pheromone components and 12 g of sticky material (Tanglefoot, Contech Electronics,

Rochester, NY, USA) were dissolved in 6 mL of hexane. This mixture was then poured to a sticky plastic zipper bag (30 □ 30 cm, HG Pack, Gunpo, Korea)."

Comment #1-8: Line 132: Give the purity of α-tocopherol.

Response: Added

Comment #1-9: Line 138: explain the use of a dark room

Response: Added as follows: "to avoid visual cue for the choice test"

Comment #1-10: Line 143: here, normally the Y-tube should be cleaned with methanol after each replication not only before and after the bio assay.

Response: As you see, we wrote the cleaning steps.

Comment #1-11: Line 143: give the sex of individuals: only females? Please justify

Response: Sex was discriminated. This information is added as follows: "Each test used 10 □ 30 individuals of each sex and was replicated four times by changing the source and control for replication."

Comment #1-12: Line 182: information about pesticides used in fields are missing here. Please add the active molecules of these insecticides.

Response: Added as follows: "Both fields were frequently treated with chemical insecticides including spinosad during the assay."

Comment #1-13: Line 206-211: add references about used primers.

Response: Added

Comment #1-14: Line 218: reference is missing.

Response: Added

Comment #1-15: Line 220: Do you check the normality of values before using the one-way analysis of variance (ANOVA) ? please add the information.

Response: Added as follows "Percentage data were arsine-transformed and confirmed in normality using PROC UNIVARIATE in the SAS program [29]."

[Reviewer #2]

Comment #2-1: The manuscript does not provide a proper paragraph of Conclusions which must be drawn appropriately based on the data presented in the text. The data obtained in the study support the conclusions. All the experiments, trials have been conducted rigorously , with appropriate control and replication. So that also the descriptions of Material and Methods must be precse and complete. Although the manuscript has been presented in a correct and standard English, it might be useful to ask for a revision of the text by a mother language lecturer.

Response:

(1) At the end of Discussion, the conclusion is described as follows: “This control technique effectively suppressed the outbreak of F. occidentalis in a field assay and also prevented the occurrence of plant disease caused by TSWV in hot peppers. Although our current study directly evaluated the pulling efficacy of the attracting CAN trap, the pushing efficacy of the repellent fence treatment was indirectly predicted from a small-scale greenhouse experiment. Subsequent studies are needed to assess thrips occurrence outside of a test greenhouse treated with the alarm pheromone to improve the estimation of the pushing efficacy in field conditions.” This also explains a limitation of this technology in field conditions.

(2) Some details are added in M&M. These are marked in red color.

(3) Text is cleaned by a English-editing company, Harrisco, co.

[Reviewer #3]

Comment #3-1: Results section appears to be missing the F-values, and degrees of freedom in the reporting of the results of the ANOVA. There appear to be sections of the results section that might actually belong in the methods section, for example the details about the trap types and configuration.

Response: All F values are added. The trap was newly designed. Thus in M&M, the structure and detailed configuration were explained. In Results, the background rationale to construct the trap was explained.

Comment #3-2: The results reported for the trap comparison test use a Chi-squared value rather than an F-value which would be expected from the ANOVA that was purported to be used to analyze the results. The statistics reported in the results section do not match the statistical tests that were delineated in the methods section. Lines 304-306 only a P-value is reported, what statistical test was used here and what was the associated value? Additionally, were the data tested to confirm that they meet the assumptions of the statistical tests chosen for analysis? Did the data transformation correct for normality?

Response: All the percent data were transformed by arcsine and tested by PROC UNIVARIATE for normality. This information is added. All the F test values are added.

Comment #3-3: In the discussion section there is a lack of discussion about any potential shortcomings/weaknesses of the study and the discussion section should have more time spent discussing future directions.

Response: At the end of Discussion, the information is described as follows: “This control technique effectively suppressed the outbreak of F. occidentalis in a field assay and also prevented the occurrence of plant disease caused by TSWV in hot peppers. Although our current study directly evaluated the pulling efficacy of the attracting CAN trap, the pushing efficacy of the repellent fence treatment was indirectly predicted from a small-scale greenhouse experiment. Subsequent studies are needed to assess thrips occurrence outside of a test greenhouse treated with the alarm pheromone to improve the estimation of the pushing efficacy in field conditions.” This also explains a limitation of this technology in field conditions.

Comment #3-4: The abstract could use more information regarding the results of the study.

Response: The additional information is added as follows: “Field assay demonstrated the efficacy of the push-pull tactics by reducing thrips density in flowers of the hot peppers as well as in the monitoring traps. Especially, the enhanced mass trapping to the CAN trap compared to the conventional yellow sticky trap led to significant reduction in the thrips population. This novel push-pull technique would be applicable to effectively control F. occidentalis in field conditions.”

Comment #3-5: In the methods section under thrips rearing, it is stated that the thrips were fed a kidney bean diet for five days after germination. This could use some clarification as I believe the authors meant after hatching.

Response: Corrected as follows: "All thrips were fed a kidney bean (Phaseolus coccineus L.) diet according to the method described in our earlier study [12]."

Comment #3-6: Y-tube methods (line 137-146) could use more explanation. It says that thrips were used three days after emergence, is that referring to emergence from the egg (hatching), or emergence as adults from the pupal stage? Additionally, the methods state that each test used 10-30 individuals, were these individuals tested separately (one at a time in the olfactometer) or were they all places into the olfactometer at the same time, please clarify.

Response: Some uncertainty is rephrased as follows: "A Y-type olfactometer (the main Y-tube length, 5 cm; two branches 2 cm long; a 45° angle between the branches; inner branch diameter, 5 cm) was placed in a dark room at 25 ± 1°C to avoid visual cue for the choice test. A flow of clean (charcoal-filtered) air at a rate of 0.6 L/min was split and passed through two glass vessels containing either an odor source or control (hexane) stimuli and entered each of the branches of the Y-tube. An air supply system (Power Air Pump, Seoul, Korea) was used for air filtration and flow rate control. Before and after the bioassay, the Y-tube was cleaned with 100% methanol. Test thrips were used three days after adult emergence. Each test used 10 ~ 30 individuals of each sex and was replicated four times by changing the source and control for replication."

Comment #3-7: Line 189-190 discussing the sampling of flowers states: "in flowers in 10 randomly selected flowers was counted in three fields three times a week for 190 one month (June 13 □ July 13) once in a week". This statement is confusing, was the sampling conducted three times a week or one time per week?

Response: This is rephrased as follows: "Number of F. occidentalis in flowers was counted from 10 randomly selected flowers with three replications in each field. The monitoring was performed once a week for one month (June 13 □ July 13)."

Comment #3-8: Line 222- should be arcsine perhaps?

Response: Corrected as suggested

Comment #3-9: In discussing the results of the field study of the push and pull components, it is stated that the Frankliniella occidentalis numbers are lower in the experimental greenhouse, but what were the results for each life stage and sex? Was it only the total number of all life stages and males and females that were reduced, or was there a difference in the response of the different life stages (larvae versus adults) or sexes?

Response: We did not examine larval density because we assessed the efficacy mostly based on the trapping adults on the yellow sticky trap. However, the figure 6C suggests the total number of larvae and adults. Thus the reduction by the push-pull can be explained by the total reduction not only by adults.
