# Supplementary material for: A push-pull strategy to control the western flower thrips, Frankliniella occidentalis, using alarm and aggregation pheromones
Source: PLoS One. 2023 Feb 24;18(2):e0279646. doi: 10.1371/journal.pone.0279646 (PMC9956899; doi:10.1371/journal.pone.0279646)
Supplement: Attachment — Submitted filename: PONE-D-22-23616.docx [file pone.0279646.s003.docx]

PLOS ONE

# A push-pull strategy to control the western flower thrips, *Frankliniella occidentalis*, using alarm and aggregation pheromones

--Manuscript Draft--

| **Manuscript Number:** | PONE-D-22-23616 |
| --- | --- |
| **Article Type:** | Research Article |
| **Full Title:** | A push-pull strategy to control the western flower thrips, Frankliniella occidentalis, using alarm and aggregation pheromones |
| **Short Title:** | Push-pull strategy |
| **Corresponding Author:** | Yonggyun Kim  Andong National University Andong, KOREA, REPUBLIC OF |
| **Keywords:** | *Frankliniella occidentalis*; aggregation pheromone; alarm pheromone; push-pull strategy; hot pepper |
| **Abstract:** | Since the first report in 1993 in Korea, the western flower thrips*, Frankliniella* *occidentalis*, has been found in various crops throughout the country. Although more than 20 different chemical insecticides are registered to control this insect pest, its outbreaks seriously damage crop yields, especially in greenhouses. This study developed a non-chemical technique to control *F. occidentalis* infesting hot peppers cultivated in greenhouses. The method was based on behavioral control using an alarm pheromone (“Push”) to prevent the entry of the thrips into greenhouses and an aggregation pheromone (“Pull”) for mass trapping inside the greenhouses. The greenhouse fences were treated with a wax formulation of the alarm pheromone and a yellow CAN trap covered with sticky material containing the aggregation pheromone was constructed and deployed inside the greenhouses. This push-pull technique effectively suppressed thrips populations in field conditions. |
|  |  |
| **Order of Authors:** | Chul-Young Kim |
|  | Falguni Khan |
|  | Yonggyun Kim |
| **Additional Information:** |  |
| **Question** | **Response** |
| **Financial Disclosure**  Enter a financial disclosure statement that describes the sources of funding for the work included in this submission. Review the [submission guidelines](http://journals.plos.org/plosone/s/submission-guidelines#loc-financial-disclosure-statement) for detailed requirements. View published research articles from [*PLOS ONE*](http://journals.plos.org/plosone/) for specific examples.  This statement is required for submission and **will appear in the published article** if the submission is accepted. Please make sure it is accurate. | The funders had no role in study design, data collection and analysis, decision to publish, or preparation of the manuscript. |

| **Unfunded studies**  Enter: *The author(s) received no specific funding for this work.*  **Funded studies**  Enter a statement with the following details:   - Initials of the authors who received each award - Grant numbers awarded to each author - The full name of each funder - URL of each funder website - Did the sponsors or funders play any role in the study design, data collection and analysis, decision to publish, or preparation of the manuscript? - **NO** - Include this sentence at the end of your statement: *The funders had no role in study design, data collection and analysis, decision to publish, or preparation of the manuscript.* - **YES** - Specify the role(s) played. |  |
| --- | --- |
| * typeset |  |
| **Competing Interests**  Use the instructions below to enter a competing interest statement for this submission. On behalf of all authors, disclose any [competing interests](http://journals.plos.org/plosone/s/competing-interests) that could be perceived to bias this work—acknowledging all financial support and any other relevant financial or non- financial competing interests.  This statement is required for submission and **will appear in the published article** if the submission is accepted. Please make sure it is accurate and that any funding sources listed in your Funding Information later in the submission form are also declared in your Financial Disclosure statement.  View published research articles from  [*PLOS ONE*](http://journals.plos.org/plosone/) for specific examples. | The authors have declared that no competing interests exist. |

| **NO authors have competing interests**  Enter: *The authors have declared that no competing interests exist.*  **Authors with competing interests**  Enter competing interest details beginning with this statement:  *I have read the journal's policy and the authors of this manuscript have the following competing interests: [insert competing interests here]* |  |
| --- | --- |
| * typeset |  |
| **Ethics Statement**  Enter an ethics statement for this submission. This statement is required if the study involved:   - Human participants - Human specimens or tissue - Vertebrate animals or cephalopods - Vertebrate embryos or tissues - Field research   Write "N/A" if the submission does not require an ethics statement.  General guidance is provided below. Consult the [submission guidelines](http://journals.plos.org/plosone/s/submission-guidelines#loc-guidelines-for-specific-study-types) for detailed instructions. **Make sure that all information entered here is included in the Methods section of the manuscript.** | N/A |

| **Format for specific study types**  **Human Subject Research (involving human participants and/or tissue)**   - Give the name of the institutional review board or ethics committee that approved the study - Include the approval number and/or a statement indicating approval of this research - Indicate the form of consent obtained (written/oral) or the reason that consent was not obtained (e.g. the data were analyzed anonymously)   **Animal Research (involving vertebrate animals, embryos or tissues)**   - Provide the name of the Institutional Animal Care and Use Committee (IACUC) or other relevant ethics board that reviewed the study protocol, and indicate whether they approved this research or granted a formal waiver of ethical approval - Include an approval number if one was obtained - If the study involved *non-human primates*, add *additional details* about animal welfare and steps taken to ameliorate suffering - If anesthesia, euthanasia, or any kind of animal sacrifice is part of the study, include briefly which substances and/or methods were applied   **Field Research**  Include the following details if this study involves the collection of plant, animal, or other materials from a natural setting:   - Field permit number - Name of the institution or relevant body that granted permission |  |
| --- | --- |
|  |  |
| **Data Availability**  Authors are required to make all data underlying the findings described fully available, without restriction, and from the time of publication. PLOS allows rare exceptions to address legal and ethical concerns. See the [PLOS Data Policy](http://journals.plos.org/plosone/s/data-availability) and [FAQ](http://journals.plos.org/plosone/s/data-availability#loc-faqs-for-data-policy) for detailed information. | Yes - all data are fully available without restriction |

| A Data Availability Statement describing where the data can be found is required at submission. Your answers to this question constitute the Data Availability Statement and **will be published in the article**, if accepted. |  |
| --- | --- |
| **Important:** Stating ‘data available on request from the author’ is not sufficient. If your data are only available upon request, select ‘No’ for the first question and explain your exceptional situation in the text box. |  |
| Do the authors confirm that all data underlying the findings described in their manuscript are fully available without restriction? |  |
| **Describe where the data may be found in full sentences. If you are copying our sample text, replace any instances of XXX with the appropriate details.**   - If the data are **held or will be held in a public repository**, include URLs, accession numbers or DOIs. If this information will only be available after acceptance, indicate this by ticking the box below. For example: *All XXX files are available from the XXX database (accession number(s) XXX, XXX.*). - If the data are all contained **within the manuscript and/or Supporting Information files**, enter the following: *All relevant data are within the manuscript and its Supporting Information files.* - If neither of these applies but you are able to provide **details of access elsewhere**, with or without limitations, please do so. For example:   *Data cannot be shared publicly because of [XXX]. Data are available from the XXX Institutional Data Access / Ethics Committee (contact via XXX) for researchers who meet the criteria for access to confidential data.*  *The data underlying the results presented in the study are available from (include the name of the third party* | All relevant data are within the manuscript and its Supporting Information files. |

| *and contact information or URL).*   - This text is appropriate if the data are owned by a third party and authors do not have permission to share the data.   * typeset |  |
| --- | --- |
| Additional data availability information: |  |

Manuscript	[Click here to access/download;Manuscript;Paper (PLoS ONE)- Text.docx](https://www.editorialmanager.com/pone/download.aspx?id=31719242&guid=2cb2f0f9-6c07-4931-a443-62cf237652b0&scheme=1)

1	*PLoS ONE*

2

# 3 A push-pull strategy to control the western flower thrips, *Frankliniella*

4 ***occidentalis*, using alarm and aggregation pheromones**

5

6

## 7 Chul-Young Kim, Falguni Khan, and Yonggyun Kim

8

9 *Department of Plant Medicals, College of Life Sciences, Andong National University, Andong*
10 *36729, Republic of Korea*

11

12

13

14

15	Corresponding author: [hosanna@anu.ac.kr](mailto:hosanna@anu.ac.kr)

16

17

18

19	**Abstract**

20

21 Since the first report in 1993 in Korea, the western flower thrips, *Frankliniella occidentalis*,
22 has been found in various crops throughout the country. Although more than 20 different
23 chemical insecticides are registered to control this insect pest, its outbreaks seriously damage
24 crop yields, especially in greenhouses. This study developed a non-chemical technique to
25 control *F. occidentalis* infesting hot peppers cultivated in greenhouses. The method was based
26 on behavioral control using an alarm pheromone (“Push”) to prevent the entry of the thrips into
27 greenhouses and an aggregation pheromone (“Pull”) for mass trapping inside the greenhouses.
28 The greenhouse fences were treated with a wax formulation of the alarm pheromone and a
29 yellow CAN trap covered with sticky material containing the aggregation pheromone was
30 constructed and deployed inside the greenhouses. This push-pull technique effectively
31 suppressed thrips populations in field conditions.

32

33 **Keywords**: *Frankliniella occidentalis*, aggregation pheromone, alarm pheromone, push-pull
34 strategy, hot pepper

35

36

## 37 1. Introduction

38

39 The western flower thrips, *Frankliniella occidentalis* (Pergande) (Thysanoptera: Thripidae),
40 is one of the most devastating insect pests to many horticultural crops including more than 500
41 species spanning 50 plant families, especially those cultured in greenhouses [1-3]. Both the
42 larval and adult stages cause damage to plants by directly feeding on leaves or flowers [4,5].
43 Especially, adults transmit plant viruses including tomato spotted wilt virus (TSWV) [6,7].
44 TSWV infections have become serious in several horticultural products in Korea, including the
45 high-value hot pepper crop [8]. This pest, originally native to North America, has spread to
46 more than 60 countries since the late 1970s, including Europe, Asia, and Australia [9]. It was
47 first observed on Jeju Island in 1993 and now has been spread to most regions in Korea [10].
48 Different reproductive modes along with a short lifecycle allow the thrips population to
49 rapidly increase during early and late seasons, in which female progeny develop from fertilized
50 eggs while males develop from unfertilized eggs [11,12]. The nutrient-based oogenesis of *F.*
51 *occidentalis* is well-programmed in females by gonadotropic hormones including juvenile
52 hormone, ecdysone, and prostaglandin [13,14]. To suppress thrips outbreaks, more than 20
53 kinds	of	chemical	insecticides	including	organophosphate,	carbamate,	pyrethroids,
54 neonicotinoids, avermectin, and spinosad are registered and applied to different agricultural
55 crops [15]. However, the control efficacy against *F. occidentalis* is often unsatisfactory due to
56 its cryptic ability to avoid exposure to the chemical spray, and the development of insecticide
57 resistance [16]. A resistant population to emamectin benzoate showed a 356-fold increased
58 resistance by a specific mutation in the target site and an over-expression of a detoxifying
59 enzyme compared to a susceptible strain in Korea [17]. As alternative control tactics, the use
60 of natural enemies, microbial pathogens, and other biological control agents has been proposed
61 under an integrated insect pest management (IPM) program against *F. occidentalis* [18]. In this
62 IPM program, behavioral control using pheromones has been considered in the monitoring and
63 mass trapping of thrips [19].
64 Chemical communication using pheromones plays a crucial role in mediating *F.*
65 *occidentalis* populations [9]. A contact pheromone, 7-methyltricosane produced by male adults,
66 acts as a territorial signal by eliciting a fight signal to other males or gives the location of a
67 male to females [20]. Kirk and Hamilton [21] suggested the presence of male-producing sex
68 pheromones attracting only females. The aggregation pheromone of *F. occidentalis* was
69 identified as containing two components, neryl methylbutanoate (NMB) and lavandulyl acetate
70 (LA) [22]. Kirk et al. [23] suggested another type of chemical communication in mating, an
71 anti-aphrodisiac pheromone produced by males during mating to prevent the multiple mating
72 of females [24]. *F. occidentalis* larvae release an alarm pheromone in an anal droplet containing
73 decyl acetate (10:Ac) and dodecyl acetate (12:Ac), in which the two components are present in
74 a relative mass ratio of 0.4:1 to 1.1:1, respectively [25]. This alarm signal promotes the
75 vigilance of thrips as well as takeoff or refuge-seeking behaviors to survive predator attacks
76 [26]. Moreover, the relative ratio of the two components and their total amounts vary with the
77 hazard intensity by increasing the relative amount of 12:Ac or the total alarm pheromone
78 concentration in the droplet, suggesting that the alarm signal is context-dependent [27].
79 Using aggregation and alarm pheromones, this study devised a push-pull control strategy
80 against *F. occidentalis* in greenhouse conditions. Fence treatment with alarm pheromone was
81 used to prevent the entry of thrips into the greenhouse while an aggregation pheromone was
82 used for mass trapping inside the greenhouse. To investigate this control strategy, we tested the
83 active pheromone compounds and determined their blend ratio in the laboratory. For field
84 assays, both pheromones were formulated for sustainability in field conditions. Finally, the
85 push-pull tactic was applied to a specific farm without chemical sprays and the control efficacy
86 was compared to that of farms that followed a conventional insecticide spraying program for
87 thrips.

88

## 89 2. Materials and methods

90

91	*2.1. Thrips rearing*

92

93 A laboratory colony of *F. occidentalis* that originated from a local population in Jeonju in
94 Korea was donated by the Rural Development Administration (Jeonju, Korea). It was
95 maintained under 25 ± 1⁰ C constant temperature and a 16:8 h (L:D) photoperiod with a
96 relative humidity of 60 ± 5%. Another laboratory colony of *F. intonsa* was obtained from a
97 field collection in a hot pepper field in Geosan (N36.81 E127.78, Korea) and reared in
98 laboratory conditions. All thrips were fed a kidney bean (*Phaseolus coccineus* L.) diet for five

99

100

days after germination according to the method described in our earlier study [12].

101

102

2.2. *Pheromones*

103 Lavandulyl acetate (LA), lavandulyl methylbutanoate (LMB), and neryl methylbutanoate
104 (NMB) were synthesized (AD, Inc., Andong, Korea) (S1 Fig). Briefly, LA and LMB were
105 synthesized from the precursor, lavandulol (Sigma-Aldrich Korea, Seoul, Korea) in
106 dichloromethane, which was reacted with acetyl chloride to yield LA, and reacted with
107 isovaleryl chloride to yield LMB, respectively, under the catalytic activity of trimethylamine.
108 NMB was used in a reaction with geraniol (Sigma-Aldrich, Korea) and isovaleryl chloride
109 under the catalytic activity of trimethylamine. All products were extracted with ethyl acetate
110 and produced 70 ~ 75% yields. The purity of the compounds was analyzed by gas
111 chromatography (8860 GC, Agilent, Santa Clara, CA, USA) with a DB-1 column (15 m ×
112 0.350 mm, Agilent), an oven temperature of 280℃, and a flow rate of 1.0 mL/min, and was
113 determined to be 98.1% for LA, 98.1 for LMB, and 99.3% for NMB. All synthesized products
114 were in the form of a racemic mixture. Two alarm pheromone components (10:Ac and 12:Ac)
115 were purchased from Signal-Aldrich Korea and dissolved in hexane to prepare different

concentrations.

2.3. *Construction of CAN trap*

120 The CAN trap consisted of a short supporting bar (1 m), an empty can (diameter 17 cm and
121 height 18.5 cm), and a sticky plastic bag (see Fig 2). The short supporting bar and the empty
122 can were connected with nails and attached to another long supporting bar (1 m) at an
123 appropriate position for the trapping height. A sticky plastic zipper bag (30 × 30 cm, HG Pack,
124 Gunpo, Korea) contained 2 mg of pheromone components and 12 g of sticky material
125 (Tanglefoot, Contech Electronics, Rochester, NY, USA) in 6 mL of hexane. After spreading the

sticky mixture, the bag was used to cover the can by inside-out inversion.

2.4. *Wax formulation of alarm pheromone*

130 The alarm pheromone was formulated in wax by the method of Kim et al. [28]. Briefly,
131 paraffin wax (Merck, Rahway, NJ, USA) was dissolved and mixed with an emulsifier (Almax
132 3600, Illshinwells, Seoul, Korea), α-tocopherol, and jojoba oil (see Fig 5A). After the alarm

pheromone was added, the slurry was cooled to solidify.

135 *2.5. Aggregation behavior test using Y-tube olfactometer*
137 A Y-type olfactometer (the main Y-tube length, 5 cm; two branches 2 cm long; a 45^o^ angle
138 between the branches; inner branch diameter, 5 cm) was placed in a dark room at 25 ± 1^o^C. A
139 flow of clean (charcoal-filtered) air at a rate of 0.6 L/min was split and passed through two
140 glass vessels containing either an odor source or control (hexane) stimuli and entered each of
141 the branches of the Y-tube. An air supply system (Power Air Pump, Seoul, Korea) was used for
142 air filtration and flow rate control. Before and after the bioassay, the Y-tube was cleaned with
143 100% methanol. Test thrips were used three days after emergence. Each test used 10 ~ 30
144 individuals and was replicated four times by changing the source and control for replication.
145 The response was positive when the thrips passed through the midpoint of the branches for 10

min. Otherwise, the thrips’ response was recorded as “no response.”

148 *2.6. Alarm pheromone test using dispersal behavior assay*
150 An arena was designed using a Petri dish (10 × 3 cm, SPL Life Science, Seoul, Korea) to
151 test the dispersion behavior of the thrips. Test thrips (20 individuals per replication) on the bean
152 diet were put into the center of the dish. A disc (3-mm diameter filter paper) was put near the
153 diet and 10 μL of test solution in hexane was added. After 1 min, the thrips dispersed away
154 from the bean were counted. Hexane was used as the control. Each treatment was replicated

three times.

157 *2.7. Field test for alarm pheromone*
159 A greenhouse (220 m^2^) cultivating hot peppers was used in this test. *F. occidentalis* was
160 heavily infesting the peppers. In the middle of the greenhouse, test hot peppers in pots (one
161 plant per pot) without any thrips were randomly assigned by a randomized block design with
162 three replications. Each test plant was covered with a plastic cage (45 × 45 × 45 cm), which
163 had large openings in five directions to allow the entry of nearby thrips. The wax-formulated
164 pheromone (5 g) was loaded in a small pheromone cage (Green Agrotech, Kyungsan, Korea),
165 which was hung at the opening gates of the cage. An empty pheromone cage was used as the
166 control.
167 The duration of alarm pheromone efficacy was tested by harvesting the lure in the field
168 conditions once a week. The efficacy test was conducted using the dispersion behavior assay

described above.

171 *2.8. Field test for push-pull tactics*
173 A test greenhouse (660 m^2^) cultivating about 200 hot peppers was located in Gudam (GD,
174 N36.54 E128.46), Andong, Korea (see Fig 6A). Alarm pheromones in the wax formation were
175 applied by pasting each (5 g) to the fence around the greenhouse at 2-m intervals for pushing
176 the thrips immigrating from outside. The alarm pheromones were re-applied every four weeks.
177 Twenty CAN traps containing aggregation pheromone were installed inside the greenhouse for
178 pulling the thrips. The sticky bags were replaced every week. During the assay, no chemical
179 insecticides were applied. For reference, thrips presence was compared in two other fields
180 cultivating hot peppers. One was a greenhouse in Hahoe (HH, about 4 km from GD) and an
181 open field in Songchun (SC, 16 km from GD) (Fig. 6A). Both fields were frequently treated

with chemical insecticides during the assay.

*2.9. Thrips monitoring test in the field*

186 Three yellow sticky traps (Green Agrotech) were installed inside the greenhouse for
187 monitoring changes in the thrips population from March to July. Thrips were identified based
188 on the morphological characters described in an earlier study [12]. Number of *F. occidentalis*
189 in flowers in 10 randomly selected flowers was counted in three fields three times a week for
190 one month (June 13 ~ July 13) once in a week. TSWV infection rates in the hot peppers were
191 measured by ring-spot disease symptoms on leaves and fruits. Each experimental unit was 100
192 randomly chosen hosts and was replicated three times. To determine TSWV-infected thrips,
193 multiplex polymerase chain reaction (PCR) analysis (see below) was used to discriminate
194 viruliferous thrips. In each site/collection, 30 thrips were used to analyze the viruliferous thrips

for the period.

197 *2.10. Molecular diagnosis of TSWV using multiplex PCR*

| 198 |  |
| --- | --- |
| 199 | A single insect was homogenized in 35 μL of an RNA extraction solution (LGC Bioresearch |
| 200 | Technologies, Hoddesdon, UK) in a 1.5-ml sample tube. The homogenate was incubated at 95 ℃ |
| 201 | for 5 min and centrifuged at 13,500 × *g* for 5 min to obtain supernatant. To extract RNA from |
| 202 | hot peppers, a piece of a leaf (1 g) was homogenized in 250 μL of a lysis buffer in the Viral |

203 DNA/RNA Extraction Kit (Intron Biotechnology, Sungnam, Korea) and RNA was extracted
204 according to the manufacturer’s instruction. PCR amplification was performed in a total
205 volume of 20 μL containing 4 μL of template RNA, 8 μL of primers, and 8 μL of SuPrime
206 Script RT-PCR Premix (2X) (Genetbio, Daejeon, Korea). Seven primers were designed for the
207 TSWV	(5’-TGTCTAAGGTTAAGCTCACTAAGGAA-3’	and	5’-
208 TTAAGCAAGTTCTGCAAGTATTGCCTG-3’), *F. occidentalis* (forward primer, 5’-GGT
209 CGC TTC ACC GCT TCC CG-3’), *F. intonsa* (forward primer, 5’-GAC CAG ACT GTT CCG
210 AGA-3’), a common reverse primer (5’-CTC TCC TGA ACW GAG GCT G-3’), and *T. tabaci*
211 (5’-TCT AAA CAG AGG GAA AGG TG-3’ and 5’-AGT GTG CCA ACA AGG CAA TG-3’).
212 Reverse transcription was performed at 50^o^C for 30 min. After a denaturation step at 95 °C for
213 5 min, the subsequent PCR was proceeded according to the following temperature cycle
214 program: 35 cycles of 95°C for 30 s, 55°C for 30 s, and 72°C for 1 min. The PCR products
215 were electrophoresed on 2% (w/v) agarose gel containing 1 × TAE buffer and were visualized
216 by UV light on a Gel Doc imaging system (Bio-Rad, Hercules, CA, USA). The resulting PCR
217 product sizes were 777 bp for TSWV, 287 bp for *F. occidentalis*, 367 bp for *F. intonsa*, and 417

bp for *T. tabaci*.

*2.11. Statistical analysis*

222 Percentage data were arsine-transformed. Data obtained from the Y-tube or choice test were
223 subjected to a one-way analysis of variance (ANOVA) using PROC GLM in the SAS program
224 [29]. Field assay data were assessed by two-way ANOVA. The means were compared using
225 the least significant difference (LSD) test at a Type I error of 0.05. Frequency data were
226 analyzed by the mean difference test using PROC FREQ.

## 3. Results

3.1. *Optimal pheromone composition to attract thrips*

232 The ability of two components (LA and NMB) of the aggregation pheromone to attract
233 thrips was tested using two thrips, *F. occidentalis* and *F. intonsa* (Fig 1). In the behavioral
234 assays, methylbutanoate lavandulol (LMB) was used as the control (Fig 1A). LA and NMB
235 were significantly (*P* < 0.05) effective in attracting adult female *F. occidentalis* and male *F.*
236 *intonsa*. However, LMB did not significantly attract thrips. Then, we prepared blends
237 containing different ratios (1:2, 1:4, and 1:7, g/g) of LA and NMB and compared their
238 attractiveness (Fig 1B). Except for the 1:2 ratio blend, the other two blends effectively attracted
239 both thrips species. Even though males did not show significant preferences, *F. occidentalis*
240 females were significantly (*P* < 0.05) attracted to the 1:7 blend whereas *F. intonsa* females were

significantly (*P* < 0.05) attracted to the 1:4 blend (Fig 1C).

3.2. *Development of the yellow CAN trap containing aggregation pheromone*

245 A yellow sticky trap (PLATE-type) has been used to monitor thrips. To enhance the trapping
246 efficiency, a CAN-type trap covering an empty can with a sticky zipper bag (CAN-type) was
247 designed to attract thrips from all directions because the PLATE-type attracted only to the front
248 and back sides. When the attractiveness of the PLATE-type trap was compared to that of the
249 CAN-type trap without any aggregation pheromone treatment, they were not significantly
250 different based on the number of thrips per sticky unit area (Fig 2A). However, the trap color
251 significantly (*P* < 0.05) influenced thrips trapping, in which attractiveness was nearly lost in
252 black traps (Fig 2B). The addition of the aggregation pheromone to the CAN trap significantly
253 (*P* < 0.05) enhanced attractiveness even in a black background (Fig 2C). With a yellow
254 background, the addition of the aggregation pheromone increased the attractiveness of the CAN
255 trap by almost five-fold compared to the trap without the pheromone. Based on the findings, a
256 yellow CAN trap was constructed (Fig 2D). For the CAN trap, LA (0.25 mg) and NMB (1.75
257 mg) were mixed with a sticky material (12 g of sticky material in 6 mL of hexane). The mixture
258 was then poured into a zipper bag and spread using a roller. When the sticky bag was applied
259 to the CAN trap, the sticky inside was inverted to the outside. The height of the CAN trap was

controlled by the length of the supporting bar (Fig 2E).

3.3. *Trapping efficiency of CAN trap in field conditions*

264 The trapping efficiency of the CAN trap for *F. occidentalis* in field conditions was
265 compared to that of a yellow sticky plate (PLATE), which is currently used to monitor thrips
266 including *F. occidentalis* (Fig 3). When we compared the trapping efficiency of these two traps
267 for a week (June 8 ~ June 15), the CAN trap showed > 4-times higher efficiency than the
268 PLATE-type (Fig 3A). Then, we analyzed the number of *F. occidentalis* collected by the two
269 traps over five successive weeks (Fig 3B). The collection numbers were normalized by sticky
270 unit area because of their different sizes. Except for one week in which thrips occurred at a low
271 density, the CAN trap was much more attractive than the PLATE trap (*t* = 5.29; df = 24; *P* <

272	0.0001).

273 Unexpectedly, the CAN trap was also highly efficient (*t* = 9.99; df = 24; *P* < 0.0001) in
274 attracting the whiteflies of *B. tabaci* compared to the PLATE-type. Thrips collected by the CAN
275 trap were classified into different species and the composition was compared to that of the
276 thrips collected by the PLATE trap (Fig 3C). The CAN trap containing the *F. occidentalis*
277 aggregation pheromone attracted all three thrips species of *F. occidentalis*, *F. intonsa*, and *T.*
278 *tabaci* but it attracted more *F. occidentalis* than the PLATE trap without pheromone (*X^2^* = 14.03;

df = 2; *P* = 0.0009).

3.4. *Development of thrips repellent using alarm pheromone*

283 In response to the alarm pheromone (10:Ac and 12:Ac = 1: 1.5, g/g), larvae and adults
284 exhibited dispersal behavior within a few seconds (Fig 4A). This dispersal behavior was also
285 observed in response to individual components, in which 12:Ac induced the behavior in both
286 stages, whereas 10:Ac was effective only in adults (Fig 4B). Dispersal behavior to the alarm
287 pheromone components was highly induced in adults compared to larvae. The two-component
288 blend was more effective in inducing the dispersal behavior than single component treatments
289 except for the comparison of 12:Ac and the blend in the adult stage. To clarify the effectiveness
290 between the single-component (12:Ac) and the two-component blend, different doses were
291 applied and the efficacy of inducing dispersal behavior was assessed (Fig 4C). The two-
292 component blend was much more effective at low doses compared to the single component in
293 both larvae and adults. In addition, the two-component blend was effective in inducing the
294 dispersal behavior of *F. intonsa* (Fig 4D). However, the dispersal behavior was highly induced
295 in *F. occidentalis* compared to *F. intonsa* at low doses. These results indicate that the alarm
296 pheromone induced the dispersal behavior of thrips in both larvae and adults and acted as a
297 repellent because the thrips fled the pheromone source.
298 The alarm pheromone-based repellent was tested in field conditions. The repellent was
299 formulated with wax for applying by pasting (Fig 5A). To test the efficacy of the formulation,
300 the repellents were applied to hot peppers infested with *F. occidentalis* in a greenhouse (Fig
301 5B). Test plants without any thrips were covered with a plastic cage, which had large openings
302 in four directions to allow the entry of nearby thrips. The repellents were then applied to the
303 openings. In the control without the repellent, thrips arrived within a day and maintained an
304 immigrating population. However, hot peppers treated with the repellents maintained
305 significantly (*P* < 0.0001) low levels of thrips for at least two weeks (Fig 5C). The repellent
306 efficacy of the wax formulation was maintained for four weeks without significant (*P* < 0.05)

differences in the greenhouse conditions.

*3.4. Application of the push-pull strategy to an agricultural farm cultivating hot peppers*

311 The occurrence of *F. occidentalis* in three different greenhouses cultivating hot peppers in
312 Andong was compared (Fig 6A). Push-pull control was applied to the GD greenhouse (Fig 6B),
313 while HH and SC were negative controls and heavily treated with conventional insecticides, in
314 which HH hot peppers were in a greenhouse, but SC peppers were in an open field. *F.*
315 *occidentalis* adults usually occur in greenhouses in late March in Andong [12]. Our current
316 study also found that the first adults were caught in greenhouses on March 23 in HH and April
317 27 in GD. In contrast, the occurrence of adults was delayed and first detected in early May in
318 an SC open field. After small peaks in April-May, the main adult peak was observed in June-
319 July in all three field conditions. In the main peak, HH had many more adults than GD by more
320 than 1,000-fold. The occurrence of *F. occidentalis* adults in GD was lower than that in the SC
321 open field. *F. occidentalis* usually occurs at low levels because the thrips prefer greenhouse
322 conditions over open fields [30]. To further analyze the effect of the CAN trap, thrips
323 populations in the flowers were compared (Fig 6C). The number of *F. occidentalis* thrips in the
324 flowers was significantly lower in CAN trap-treated GD compared to the control farms (*F* =
325 5.89; df = 10, 30; *P* < 0.0001). *F. occidentalis* is a vector for TSWV. The TSWV infection rate
326 was significantly different between farms (*F* = 8.55; df = 2, 22; *P* = 0.0049). Indeed, no hot
327 peppers were infected with TSWV in CAN trap-treated GD (Fig 6D). However, the control
328 farms had TSWV-infected plants, in which the SC site showed an infection rate of more than
329 30%. This was further supported by the multiplex PCR analysis. No *F. occidentalis* was
330 infected with TSWV, whereas significant numbers of *F. occidentalis* adults were positive for

TSWV in HH and SC.

## 4. Discussion

335 This study proposes a push-pull control strategy using alarm and aggregation pheromones
336 against *F. occidentalis* in greenhouse conditions. To test these novel control tactics, the push-
337 pull effects of the chemical compounds were re-evaluated. For field assays, both pheromones
338 were formulated as wax types for the alarm pheromone and a CAN trap for the aggregation
339 pheromone. These formulated pheromones were applied to a practical farm and the control
340 efficacy was compared to those of other conventional farms treated with chemical insecticides.
341 Our laboratory and field data supported the control efficacy of push-pull control tactics against
342 *F. occidentalis*.
343 To enhance the attractiveness for *F. occidentalis*, two aggregation pheromone components
344 (LA and NMB) were re-evaluated with an analog, lavandulyl methylbutanoate (LMB). LMB
345 was identified	as	an	aggregation	pheromone	component	of	another	flower	thrips,
346 *Megalurothrips sjostedti* [31], and onion thrips, *Thrips palmi* [32]. No significant attractiveness
347 of LMB for either *F. occidentalis* or *F. intonsa* was seen by Y-tube olfactometry. However, they
348 were attracted to LA and NMB. The aggregation pheromones LA and NMB are shared between
349 *F. occidentalis* and *F. intonsa*, so species-specific blend mixtures were used to prevent cross-
350 attraction [33]. In our assay, *F. occidentalis* preferred a 1:7 (LA:NMB) ratio to a 1:4 ratio,
351 whereas *F. intonsa* exhibited an opposite preference. Interestingly, a significant preference was
352 observed only in the females of both species in the Y-tube olfactometer analysis in the double-
353 choice experiment between 1:4 and 1:7 ratios, although both sexes were attracted to both ratios
354 in the single-choice experiments. Considering that the aggregation pheromone is basically
355 attractive to both sexes, our assay might not detect a male preference. The lower sensitivity of
356 males may be their preference for a specific stereoisomer because our synthetic pheromone
357 components (S1 Fig) were mixtures of stereoisomers. The LA:NMB ratio in *F. occidentalis*
358 was reported to be within a range of 1:0.8 ~ 1:5 measured by solid-phase microextraction
359 (SPME) [22]. In that study, the addition of LA to NMB gave no increase relative to NMB alone,
360 leading to the development of a single-component commercial product. In our current study,
361 however, the inclusion of LA in the aggregation pheromone may have had some physiological
362 significance because unlike the 1:4 and 1:7 ratios, the 1:2 mixture ratio did not significantly
363 attract *F. occidentalis*. This suggests the role of LA in the aggregation signal of *F. occidentalis*.
364 Two congener species of *F. occidentalis* and *F. intonsa* share flower habitats and aggregation
365 pheromone components, in which *F. occidentalis* had 1:13 (LA:NMB) ratio while *F. intonsa*
366 had 1:2 ratio in a Chinese population measured by SPME [34]. The significance of these
367 different effective mixture ratios was confirmed in field experiments where a 1:8 ratio of
368 aggregation pheromone components caught more *F. occidentalis,* whereas a 1:4 ratio attracted
369 more *F. intonsa* [33]. Thus, the role of LA may give species-specificity to the aggregation
370 pheromone. Furthermore, in our current assay, LA alone significantly attracted *F. occidentalis*.
371 Thus, we determined the optimal mass trapping lure to be a mixture of LA and NMB at a 1:7
372 mixture ratio.
373 An alarm pheromone consisting of 10:Ac and 12:Ac induced significant dispersion
374 behavior in *F. occidentalis*. It was reported that in response to the alarm pheromone, larvae
375 exhibited escape behavior from the source by a rapid drop from host plants, whereas exposed
376 adults exhibited take-off behavior and avoided landing in the area [35]. Indeed, the deployment
377 of the alarm pheromone significantly prevented the invasion of the thrips to hot peppers in the
378 current study. These facts led us to apply the alarm pheromone to the push-pull strategy to
379 control *F. occidentalis*. Other potent repellents may be developed from other sources. For
380 example, plant odors emitted by non-preferred hosts, such as garlic (*Allium sativum*) and celery
381 (*Apium graveolens*), showed repelling effects on *F. occidentalis* females [36].
382 To apply the pheromones to field conditions, they were formulated to meet the purpose of
383 the push-pull strategy. Alarm pheromone components were formulated using wax for fence
384 treatment by pasting. The wax formulation modified the methods used in other lure
385 formulations developed using moth sex pheromone and fly lure, where their efficacy was
386 confirmed in field conditions [28,37]. The bioactivity of the wax formulation of the alarm
387 pheromone lasted for at least four weeks in greenhouse conditions. Aggregation pheromone
388 was formulated by mixing with sticky material in an attract-to-kill strategy. For this purpose, a
389 CAN trap was devised and effectively attracted thrips by its yellow color and aggregation
390 pheromone. The question has been raised of whether thrips from nearby plants or thrips flying
391 over plants were collected by the CAN traps. The study findings present indirect evidence that
392 CAN traps collected thrips flying over the plants because adult thrips in the hot pepper flowers
393 were not evenly distributed, whereas the 20 installed CAN traps collected similar quantities of
394 thrips. This suggests that our CAN traps attracted flying thrips. A synergistic effect of the CAN
395 trap and another biological control method using a natural enemy was also suggested because
396 it is known that the predator *Orius laevigatus* stimulates the release of an alarm pheromone
397 [27], which induces take-off flight in flying thrips in host plants.
398 Interestingly, the trapping efficiency of the yellow CAN trap for the whitefly *B. tabaci* was
399 enhanced without an aggregation pheromone. This suggests that the aggregation pheromone of
400 *F. occidentalis* was effective in attracting *B. tabaci*. The two aggregation pheromone
401 components are terpenoids. Whiteflies respond to various terpenoids including sesquiterpenes
402 (zingiberene and	curcumene)	and	monoterperpenes	(*p*-cymene, *α*-terpinene, and	*α*-
403 phellandrene), although terpenoids act as repellents [38]. It is noteworthy that LMB, one of the
404 aggregation pheromone components in thrips, is used by a sex pheromone of the mealybug,
405 *Phenacoccus madeirensis* [39]. Further studies assessing the sensitivity and response of *B.*
406 *tabaci* to the aggregation pheromone of *F. occidentalis* need to be conducted.
407 The push-pull strategy was effective in suppressing the frequency of TSWV-infected hot
408 peppers. Compared to other fields such as those in HH and SC, the thrips density was low in
409 flowers in GD treated with the push-pull strategy, as was the number in monitoring traps.
410 TSWV-infected hot peppers exhibit characteristic disease symptoms of ring-spots on leaves
411 and fruit [40]. Based on this disease symptom, the survey indicated no TSWV infections in hot
412 peppers in GD in contrast to the significant numbers of disease symptoms in the other two
413 fields. This was further supported by the multiplex PCR results, which indicated no TSWV-
414 infected *F. occidentalis* in GD compared to large numbers of TSWV-positive thrips in other
415 fields. This might be explained by a local variation in viruliferous thrips as similar variations
416 in *Thrips tabaci* carrying TSWV were seen [41]. Alternatively, the suppression of thrips
417 occurrence by the push-pull treatment might have caused the decrease in the TSWV infection
418 rate.
419 The push-pull strategy has been used for effective insect pest control [42]. A push-pull
420 control technique against *F. occidentalis* was proposed using UV-reflective mulch as a pushing
421 factor to disrupt thrips colonization on host plants along with a companion plant as a pulling
422 factor to attract the predators and trap the thrips [43]. Alternatively, the use of semiochemicals
423 such as attractants and repellents has been considered for developing a push-pull strategy [44].
424 Kirk et al. [23] proposed *F. occidentalis* control using pheromones and other semiochemicals.
425 This study demonstrated novel push-pull tactics using aggregation/alarm pheromones (Fig 7)
426 to control *F. occidentalis* in greenhouse conditions. This control technique effectively
427 suppressed the outbreak of *F. occidentalis* in a field assay and also prevented the occurrence of
428 plant disease caused by TSWV in hot peppers. Although our current study directly evaluated
429 the pulling efficacy of the attracting CAN trap, the pushing efficacy of the repellent fence
430 treatment was indirectly predicted from a small-scale greenhouse experiment. Subsequent
431 studies are needed to assess thrips occurrence outside of a test greenhouse treated with the

alarm pheromone to improve the estimation of the pushing efficacy in field conditions.

## Acknowledgments

436 This work was conducted with the support of the Cooperative Research Program for
437 Agriculture Science & Technology Development (Project No. PJ01578901) funded by the

Rural Development Administration, Republic of Korea.

## References

442 1. Yudin LS, Cho JJ, Mitchell WC. Host range of western flower thrips, *Frankliniella*
443 *occidentalis* (Thysanoptera: Thripidae), with special reference to *Leucaena glauca*. Environ

444	Entomol. 1986; 15:1292-1295.

445 2. Schneweis DJ, Whitfield AE, Rotenberg D. Thrips developmental stage-specific
446 transcriptome response to tomato spotted wilt virus during the virus infection cycle in
447 *Frankliniella occidentalis*, the primary vector. Virology. 2017; 500:226–237.
448 3. He Z, Guo JF, Reitz SR, Lei ZR, Wu SY. A global invasion by the thrips, *Frankliniella*
449 *occidentalis*: current virus vector status and its management. Insect Sci. 2020; 27:626-645.
450 4. Reitz SR. Biology and ecology of the western flower thrips (Thysanoptera: Thripidae): The
451 making of a pest. Fla Entomol. 2009; 92:7-13.
452 5. Shrestha A, Srinivasan R, Riley DG, Culbreath AK. Direct and indirect effects of a thrips-
453 transmitted Tospovirus on the preference and fitness of its vector, *Frankliniella fusca*.

454	Entomol Exp Appl. 2012; 145:260-271.

455 6. Belliure B, Janssen A, Maris PC, Peters D, Sabelis MW. Herbivore arthropods benefit from
456 vectoring plant viruses. Ecol Lett. 2005; 8:70-79.
457 7. Rotenberg D, Jacobson AL, Schneweis DJ, Whitfield AE. Thrips transmission of
458 tospoviruses. Curr Opin Virol. 2015; 15:80-89.
459 8. Lee S, Lee J, Kim S, Choi H, Park J, Lee J, et al. The incidence and distribution of viral
460 diseases in pepper by cultivation types. Res Plant Dis. 2004; 10:231-240.
461 9. Reitz SR, Gao Y, Kirk WDJ, Hoddle MS, Leiss KA, Funderburk JE. Invasion biology,
462 ecology, and management of western flower thrips. Annu Rev Entomol. 2020; 65:17-37.
463 10. Woo KS, Ahn SB, Lee SH, Kwon HM. First record of *Frankliniella occidentalis* and its
464 distribution and host plants in Korea. Korean J Appl Entomol. 1994; 33:127.
465 11. Ding T, Chi H, Gökçe A, Gao Y, Zhang B. Demographic analysis of arrhenotokous
466 parthenogenesis and bisexual reproduction of *Frankliniella occidentalis* (Pergande)
467 (Thysanoptera: Thripidae). Sci Rep. 2018; 8:3346.
468 12. Kim C, Choi D, Kang J, Ahmed SA, Kil E, Kwon G, et al. Thrips infesting hot pepper
469 cultured in greenhouses and variation in gene sequences encoded in TSWV. Korean J Appl

470	Entomol. 2021; 60:387-401.

471	13. Choi DY, Kim Y. PGE2 mediation of egg development in western flower thrips,

472	*Frankliniella occidentalis*. Arch Insect Biochem Physiol. 2022; e21949.

473	14. Choi DY, Kim Y. Transcriptome analysis of female western flower thrips, *Frankliniella*

474	*occidentalis*,	exhibiting	neo-panoistic	ovarian	development.	PLoS	ONE	2022;

475	17:e0272399.

476 15. Cho S, Kyung Y, Cho S, Shin S, Jeong D, Kim S, et al. Evaluation of susceptibility of
477 western flower thrips (*Frankliniella occidentalis*) and garden thrips (*F. intonsa*) to 51
478 insecticides. Korean J Appl Entomol. 2018; 57:221-231.
479 16. Bhuyain MMH, Lim UT. Relative susceptibility to pesticides and environmental conditions
480 of *Frankliniella intonsa* and *F. occidentalis* (Thysanoptera: Thripidae), an underlying
481 reason for their asymmetrical occurrence. PLoS ONE 2020; 15:e0237876.
482 17. Gao Y, Yoon KA, Lee JH, Kim JH, Lee SH. Overexpression of glutamate-gated chloride
483 channel in the integument is mainly responsible for emamectin benzoate resistance in the
484 western	flower	thrips	*Frankliniella	occidentalis*. Pest	Manag. Sci.	2022; doi:

485	10.1002/ps.7032.

486	18. Mouden S, Sarmiento KF, Klinkhamer PG, Leiss KA. Integrated pest management in

487	western flower thrips: past, present and future. Pest Manag Sci. 2017; 73:813-822.

488	19. Sampson C, Kirk WD. Can mass trapping reduce thrips damage and is it economically

489	viable? Management of the Western flower thrips in strawberry. PLoS ONE 2013;

490	8:e80787.

491 20. Olaniran OA, Sudhakar AV, Drijfhout FP, Dublon IA, Hall DR, Hamilton JG, et al. A male-
492 predominant cuticular hydrocarbon, 7-methyltricosane, is used as a contact pheromone in
493 the western flower thrips *Frankliniella occidentalis*. J Chem Ecol. 2013; 39:559-568.
494 21. Kirk WD, Hamilton JG. Evidence for a male-produced sex pheromone in the western
495 flower thrips *Frankliniella occidentalis*. J Chem Ecol. 2004; 30:167-174.
496 22. Hamilton JG, Hall DR, Kirk WD. Identification of a male-produced aggregation
497 pheromone in the western flower thrips *Frankliniella occidentalis*. J Chem Ecol. 2005;

498	31:1369-1379.

499 23. Kirk WDJ, de Kogel WJ, Koschier EH, Teulon DAJ. Semiochemicals for thrips and their
500 use in pest management. Annu Rev Entomol. 2021; 66:101-119.
501 24. Akinyemi AO, Kirk WDJ. Experienced males recognise and avoid mating with non-virgin
502 females in the western flower thrips. PLoS ONE 2019; 14:e0224115.
503 25. MacDonald KM, Hamilton JG, Jacobson R, Kirk WD. Analysis of anal droplets of the
504 western flower thrips *Frankliniella occidentalis*. J Chem Ecol. 2003; 29:2385-2389.
505 26. de Bruijn PJ, Egas M, Janssen A, Sabelis MW. Pheromone-induced priming of a defensive
506 response in Western flower thrips. J Chem Ecol. 2006; 32:1599-1603.
507 27. de Bruijn PJ, Egas M, Sabelis MW, Groot AT. Context-dependent alarm signalling in an

508	insect. J Evol Biol. 2016; 29:665-671.

509	28. Kim K, Kim M, Kwon G, Kim Y. Technologies required for development of trap-based

510	MAT control against the striped fruit fly, *Bactrocera scutellata*. Korean J. Appl. Entomol.

511	2017; 56:51-60.

512	29. SAS Institute Inc. SAS/STAT user’s guide, Release 6.03, Ed. Cary, NC. 1989.

513	30. Lee GS, Lee JH, Kang SH, Woo KS. Thrips species (Thysanoptera: Thripidae) in winter

514	season and their vernal activities on Jeju island, Korea. J Asia Pac Entomol. 2001; 4:115–

515	122.

516 31. Niassy S, Tamiru A, Hamilton JGC, Kirk WDJ, Mumm R, Sims C, et al. Characterization
517 of male-produced aggregation pheromone of the bean flower thrips *Megalurothrips*
518 *sjostedti* (Thysanoptera: Thripidae). J Chem Ecol. 2019; 45:348-355.
519 32. Akella SVS, Kirk WDJ, Lu YB, Murai T, Walters KFA, Hamilton JG. Identification of the
520 aggregation pheromone of the melon thrips, *Thrips palmi*. PLoS ONE 2014; 9:e103315.
521 33. Li X, Geng S, Zhang Z, Zhang J, Li W, Huang J, et al. Species-specific aggregation
522 pheromones contribute to coexistence in two closely related thrips species. Bull Entomol

523	Res. 2019; 109:119-126.

524	34. Zhu XY, Zhang PJ, Lu YB. Isolation and identification of the aggregation pheromone

525	released by male adults of *Frankliniella intonsa* (Thysanoptera: Thripidae). Acta Entomol

526	Sin. 2012; 55:376-385.

527	35. Teerling CR, Pierce HD, Borden JH, Gillespie DR. Identification and bioactivity of alarm

528	pheromone in the western flower thrips, *Frankliniella occidentalis*. 1993; 19:681-697.

529	36. Cao Y, Zhi J, Cong C, Margolies DC. Olfactory cues used in host selection by *Frankliniella*

530	*occidentalis* (Thysanoptera: Thripidae) in relation to host suitability. J Insect Behav. 2014;

531	27:41-56.

532 37. Jung S, Park M, Lee S, Choi K, Hong Y, Bae S. et al. Development of wax-typed pheromone
533 dispenser for mating disruption of the oriental fruit moth, *Grapholita molesta*, and its
534 application technique. Korean J Appl Entomol. 2008; 47:255-263.
535 38. Bleeker PM, Diergaarde PJ, Ament K, Guerra J, Weidner M, Schütz S, et al. The role of
536 specific tomato volatiles in tomato-whitefly interaction. Plant Physiol. 2009; 151:925-935.
537 39. Ho HY, Su YT, Ko CH, Tsai MY. Identification and synthesis of the sex pheromone of the
538 Madeira mealybug, *Phenacoccus madeirensis* Green. J Chem Ecol. 2009; 35:724-732.
539 40. Lee JS, Cho WK, Kim MK, Kwak HR, Choi HS, Kim KH. Complete genome sequences
540 of three tomato spotted wilt virus isolates from tomato and pepper plants in Korea and
541 their phylogenetic relationship to other TSWV isolates. Arch Virol. 2011; 156:725-728.
542 41. Jacobson AL, Kennedy GG. Specific insect-virus interactions are responsible for variation
543 in competency of different *Thrips tabaci* isolines to transmit different Tomato Spotted Wilt
544 Virus isolates. PLoS ONE 2013; 8:e54567.
545 42. Cook SM, Khan ZR, Pickett JA. The use of push-pull strategies in integrated pest
546 management. Annu Rev Entomol. 2007; 52:375-400.
547 43. Tyler-Julian K, Funderburk J, Srivastava M, Olson S, Adkins S. Evaluation of a push-pull
548 system for the management of *Frankliniella* species (Thysanoptera: Thripidae) in tomato.

549	Insects 2018; 9:187.

550	44. Niu Y, Han S, Wu Z, Pan C, Wang M, Tang Y. et al. A push-pull strategy for controlling the

551	tea green leafhopper (*Empoasca flavescens* F.) using semiochemicals from *Tagetes erecta*

552

553

and *Flemingia macrophylla*. Pest Manag Sci. 2022; 78:2161-2172.

554

555

## Figure captions

556 **Fig 1**. **Differential preference analysis of two flower thrips (*F. occidentalis* and *F. intonsa*)**
557 **for aggregation pheromone using a Y-tube olfactometer.** (A) Screening of active
558 components and mixture ratios in the aggregation pheromone. The pheromone components
559 were identified as LA (lavandulyl acetate), LMB (lavandulyl methylbutanoate), and NMB
560 (neryl methylbutanoate). (B) Choice tests of the thrips against two pheromone blends. Each
561 response (experimental unit) used 20 adults (< 3-days-old after emergence). Each treatment

was replicated four times. The detailed data are presented in Figure S1.

564 **Fig 2**. **Development of a yellow CAN trap (CAN) to attract *F. occidentalis* using the**
565 **aggregation pheromone.** (A) Comparison of the attractiveness of the CAN trap without
566 aggregation pheromone and a yellow sticky trap (PLATE). (B) Effect of CAN trap color on
567 attracting thrips. (C) Effect of aggregation pheromone on attracting thrips using the CAN trap,
568 in which the black CAN trap demonstrated a pheromone effect while the yellow CAN trap
569 demonstrated both pheromone and color effects. Pheromone components were prepared by
570 mixing lavandulyl acetate (LA) and neryl methylbutanoate (NMB) at a 1:7 mass ratio. Each
571 experimental unit represented a trap and was replicated three times. (D) Preparation of a sticky
572 bag for the CAN trap. (E) Dimensions (mm) of the CAN trap parts and adjustment of the CAN

trap height.

## 575 Fig 3. Field efficacy of a yellow CAN trap containing aggregation pheromone. (A)

576 Enhanced trapping efficiency of the CAN trap containing aggregation pheromone compared to
577 the yellow sticky plate (PLATE). CAN and PLATE traps used 20 and three replications,
578 respectively. An asterisk above the standard deviation bars indicates a significant difference
579 between two treatments at type I error = 0.05 (LSD test). (B) Enhanced pulling effect of the
580 CAN trap on trapping *F. occidentalis* and *B. tabaci* in different seasons. Two and three asterisks
581 above the standard deviation bars indicate significant differences between two treatments at
582 Type I error = 0.01 and type I error = 0.0001 (LSD test), respectively. NS indicates no
583 significant difference. (D) Composition of thrips trapped by the PLATE and CAN traps. The
584 average species frequencies in CAN and PLATE traps were obtained from 20 and three

replications, respectively.

587 **Fig 4**. **Development of a thrips repellent using an alarm pheromone of *F. occidentalis*.** The
588 alarm pheromone components were decyl acetate (10:Ac) and dodecyl acetate (12:Ac) at a
589 1:1.5 mass ratio. (A) Bioassay assessing repellent efficacy measured by the dispersal behavior
590 of adults and larvae from bean diet after the test component (10 μL) was dispensed onto a filter
591 paper disc. Arrows indicate the dispersal behavior of larvae. Different letters above the standard
592 deviation bars indicate a significant difference between means at type I error = 0.05 (LSD test).
593 (B) Repellent efficacy of single and dual components. (C) Dose-response of the pheromone
594 components. (D) Comparison of dual component pheromone (alarm pheromone) efficacy in *F.*
595 *occidentalis* (Fo) and *F. intonsa* (Fi). Each treatment was replicated three times. Asterisks
596 above the standard deviation bars indicate significant difference between two treatments (LSD
597 test): * at Type I error = 0.05, ** at Type I error = 0.01, and *** at Type I error = 0.0001. NS

indicates no significant difference.

600 **Fig 5**. **Field assay of alarm pheromone-based repellent efficacy against *F. occidentalis* in**
601 **a greenhouse cultivating hot peppers.** (A) Composition of the wax formulation of the
602 repellent. (B) Installation of the wax formulations (in a small cage) in five directions around a
603 test plant. (C) Effect of the repellent on preventing immigrating thrips for two weeks. Three
604 asterisks above the standard deviation bars indicate a significant difference between two
605 treatments at Type I error = 0.0001 (LSD test). (D) Determination of the effective period of the
606 wax formulation in field conditions. Different letters above the standard deviation bars indicate

a significant difference among means at Type I error = 0.05 (LSD test).

## 609 Fig 6. Application of push-pull strategy to *F. occidentalis* infesting hot peppers (200 plants)

610 **in a greenhouse (660 m^2^).** (A) A map indicating three field assay sites: Gudam (GD), Hahoe
611 (HH), and Songchun (SC). (B) Deployment of repellents and CAN traps in a GD greenhouse.
612 Adult occurrence in three sites. (C) Number of *F. occidentalis* in flowers. Each replication
613 represents the total number of thrips from 10 randomly selected flowers. Each measurement
614 was replicated three times. (D) TSWV infection rates in hot peppers and *F. occidentalis* over
615 one month (June 13 to July 13). Each measurement represents the percentage of diseased plants
616 out of 100 randomly chosen hosts. Each measurement was replicated three times. To determine
617 TSWV-infected thrips, multiplex PCR analysis was performed to discriminate viruliferous
618 thrips. In each site/collection, 30 thrips were used to analyze the viruliferous thrips for the
619 period. An asterisk above the standard deviation bars indicates a significant difference among

means at Type I error = 0.05 (LSD test). NS indicates no significant difference.

622 **Fig 7**. **A working model of the push-pull strategy against *F. occidentalis* using repellent**

## 623 (sound signal logo) and CAN trap (yellow can) in a greenhouse cultivating hot peppers.

624 Arrows indicate the push and pull moving directions.

Figure 1	[Click here to access/download;Figure;Figure1.jpg](https://www.editorialmanager.com/pone/download.aspx?id=31719243&guid=3144d50f-7cd3-465f-a8ca-e9eaf494a853&scheme=1)

Figure 2	[Click here to access/download;Figure;Figure2.jpg](https://www.editorialmanager.com/pone/download.aspx?id=31719244&guid=ffa54fdd-7804-450e-aa2f-4b0d1bac96b0&scheme=1)

,Figure 3	[Click here to access/download;Figure;Figure3.jpg](https://www.editorialmanager.com/pone/download.aspx?id=31719245&guid=d2b336a9-0432-4db5-a023-da91d03a227f&scheme=1)

Figure 4	[Click here to access/download;Figure;Figure4.jpg](https://www.editorialmanager.com/pone/download.aspx?id=31719246&guid=0cdc4063-cd0c-402f-a8e1-5b7d9d4827e1&scheme=1)

Figure 5	[Click here to access/download;Figure;Figure5.jpg](https://www.editorialmanager.com/pone/download.aspx?id=31719247&guid=c6054311-f702-4b3c-8c9e-791fdf113292&scheme=1)

Figure 6AB	[Click here to access/download;Figure;Figure6-A,B.jpg](https://www.editorialmanager.com/pone/download.aspx?id=31719248&guid=b4764ec1-f391-429a-8c3a-218d5ba01e75&scheme=1)

Figure 6CD	[Click here to access/download;Figure;Figure6-C,D.jpg](https://www.editorialmanager.com/pone/download.aspx?id=31719249&guid=292fbddd-8ef8-4e7e-8354-8fcd840b2be5&scheme=1)

Figure 7	[Click here to access/download;Figure;Figure7.jpg](https://www.editorialmanager.com/pone/download.aspx?id=31719250&guid=f9e54771-ef70-4b17-a0ea-5806cec4bb26&scheme=1)

Supporting Information

[Click here to](https://www.editorialmanager.com/pone/download.aspx?id=31719251&guid=e70d6818-1761-4c27-acb1-38db532377f2&scheme=1)

[/download](https://www.editorialmanager.com/pone/download.aspx?id=31719251&guid=e70d6818-1761-4c27-acb1-38db532377f2&scheme=1)

[**Supporting Information** Paper (PLoS ONE)-SI.docx](https://www.editorialmanager.com/pone/download.aspx?id=31719251&guid=e70d6818-1761-4c27-acb1-38db532377f2&scheme=1)
