# Supplementary material for: A push-pull strategy to control the western flower thrips, Frankliniella occidentalis, using alarm and aggregation pheromones
Source: PLoS One. 2023 Feb 24;18(2):e0279646. doi: 10.1371/journal.pone.0279646 (PMC9956899; doi:10.1371/journal.pone.0279646)
Supplement: Attachment — Submitted filename: Response to reviewers.docx [file pone.0279646.s004.docx]

**Response to reviewers’ comments**

**[Reviewer #1]**

**Comment #1-1**: Introduction. I believe that you need a much strong justification for studying the western flower thrips, Frankliniella occidentalis as pest I did not find any strong justification for using F. occidentalis compared to other insects such as aphids for example!

**Response**: The thrips as a serious pest is explained by direct feeding damage and indirect TSWV transmission. We did not find the direct analysis on the statistical data of the relative damage compared to aphids. Thus we did not add the further information.

**Comment #1-2**: Materials and methods. The overall level of details for the Materials and Methods is appropriate, however there are some informations should be add in the text:

Line 98 page 5: Please precise if it was the same laboratory conditions described below.

**Response**: It is clarified as follows: “…. reared in the laboratory conditions.”

**Comment #1-3**: Line 103-105 page 5: please give the purity of the molecules: Lavandulyl acetate (LA), lavandulyl methylbutanoate (LMB), and neryl methylbutanoate (NMB).

**Response**: Added

**Comment #1-4**: Line 108-110 page 6: Purity of molecules are missing here.

**Response**: Added

**Comment #1-5**: Line 114 page 6: add the purity of the alarm pheromone components

**Response**: added

**Comment #1-6**: Line 115: please correct Signal-Aldrich >> Sigma-Aldrich

**Response**: Corrected as suggested

**Comment #1-7**: Line 125: I did not find any justification for using hexane as solvent please explain.

**Response**: It is rephrased as follows: “To mix the pheromone with sticky material, 2 mg of pheromone components and 12 g of sticky material (Tanglefoot, Contech Electronics, Rochester, NY, USA) were dissolved in 6 mL of hexane. This mixture was then poured to a sticky plastic zipper bag (30 × 30 cm, HG Pack, Gunpo, Korea).”

**Comment #1-8**: Line 132: Give the purity of α-tocopherol.

**Response**: Added

**Comment #1-9**: Line 138: explain the use of a dark room

**Response**: Added as follows: “to avoid visual cue for the choice test”

**Comment #1-10**: Line 143: here, normally the Y-tube should be cleaned with methanol after each replication not only before and after the bio assay.

**Response**: As you see, we wrote the cleaning steps.

**Comment #1-11**: Line 143: give the sex of individuals: only females? Please justify

**Response**: Sex was discriminated. This information is added as follows: “Each test used 10  30 individuals of each sex and was replicated four times by changing the source and control for replication.”

**Comment #1-12**: Line 182: information about pesticides used in fields are missing here. Please add the active molecules of these insecticides.

**Response**: Added as follows: “Both fields were frequently treated with chemical insecticides including spinosad during the assay.”

**Comment #1-13**: Line 206-211: add references about used primers.

**Response**: Added

**Comment #1-14**: Line 218: reference is missing.

**Response**: Added

**Comment #1-15**: Line 220: Do you check the normality of values before using the one-way analysis of variance (ANOVA) ? please add the information.

**Response**: Added as follows “Percentage data were arsine-transformed and confirmed in normality using PROC UNIVARIATE in the SAS program [29].”

**[Reviewer #2]**

**Comment #2-1**: The manuscript does not provide a proper paragraph of Conclusions which must be drawn appropriately based on the data presented in the text. The data obtained in the study support the conclusions. All the experiments, trials have been conducted rigorously , with appropriate control and replication. So that also the descriptions of Material and Methods must be precse and complete. Although the manuscript has been presented in a correct and standard English, it might be useful to ask for a revision of the text by a mother language lecturer.

**Response**:

(1) At the end of Discussion, the conclusion is described as follows: “This control technique effectively suppressed the outbreak of F. occidentalis in a field assay and also prevented the occurrence of plant disease caused by TSWV in hot peppers. Although our current study directly evaluated the pulling efficacy of the attracting CAN trap, the pushing efficacy of the repellent fence treatment was indirectly predicted from a small-scale greenhouse experiment. Subsequent studies are needed to assess thrips occurrence outside of a test greenhouse treated with the alarm pheromone to improve the estimation of the pushing efficacy in field conditions.” This also explains a limitation of this technology in field conditions.

(2) Some details are added in M&M. These are marked in red color.

(3) Text is cleaned by a English-editing company, Harrisco, co.

**[Reviewer #3]**

**Comment #3-1**: Results section appears to be missing the F-values, and degrees of freedom in the reporting of the results of the ANOVA. There appear to be sections of the results section that might actually belong in the methods section, for example the details about the trap types and configuration.

**Response**: All F values are added. The trap was newly designed. Thus in M&M, the structure and detailed configuration were explained. In Results, the background rationale to construct the trap was explained.

**Comment #3-2**: The results reported for the trap comparison test use a Chi-squared value rather than an F-value which would be expected from the ANOVA that was purported to be used to analyze the results. The statistics reported in the results section do not match the statistical tests that were delineated in the methods section. Lines 304-306 only a P-value is reported, what statistical test was used here and what was the associated value? Additionally, were the data tested to confirm that they meet the assumptions of the statistical tests chosen for analysis? Did the data transformation correct for normality?

**Response**: All the percent data were transformed by arcsine and tested by PROC UNIVARIATE for normality. This information is added. All the F test values are added.

**Comment #3-3**: In the discussion section there is a lack of discussion about any potential shortcomings/weaknesses of the study and the discussion section should have more time spent discussing future directions.

**Response**: At the end of Discussion, the information is described as follows: “This control technique effectively suppressed the outbreak of F. occidentalis in a field assay and also prevented the occurrence of plant disease caused by TSWV in hot peppers. Although our current study directly evaluated the pulling efficacy of the attracting CAN trap, the pushing efficacy of the repellent fence treatment was indirectly predicted from a small-scale greenhouse experiment. Subsequent studies are needed to assess thrips occurrence outside of a test greenhouse treated with the alarm pheromone to improve the estimation of the pushing efficacy in field conditions.” This also explains a limitation of this technology in field conditions.

**Comment #3-4**: The abstract could use more information regarding the results of the study.

**Response**: The additional information is added as follows: “Field assay demonstrated the efficacy of the push-pull tactics by reducing thrips density in flowers of the hot peppers as well as in the monitoring traps. Especially, the enhanced mass trapping to the CAN trap compared to the conventional yellow sticky trap led to significant reduction in the thrips population. This novel push-pull technique would be applicable to effectively control F. occidentalis in field conditions.”

**Comment #3-5**: In the methods section under thrips rearing, it is stated that the thrips were fed a kidney bean diet for five days after germination. This could use some clarification as I believe the authors meant after hatching.

**Response**: Corrected as follows: “All thrips were fed a kidney bean (Phaseolus coccineus L.) diet according to the method described in our earlier study [12].”

**Comment #3-6**: Y-tube methods (line 137-146) could use more explanation. It says that thrips were used three days after emergence, is that referring to emergence from the egg (hatching), or emergence as adults from the pupal stage? Additionally, the methods state that each test used 10-30 individuals, were these individuals tested separately (one at a time in the olfactometer) or were they all places into the olfactometer at the same time, please clarify.

**Response**: Some uncertainty is rephrased as follows: “A Y-type olfactometer (the main Y-tube length, 5 cm; two branches 2 cm long; a 45^o^ angle between the branches; inner branch diameter, 5 cm) was placed in a dark room at 25 ± 1^o^C to avoid visual cue for the choice test. A flow of clean (charcoal-filtered) air at a rate of 0.6 L/min was split and passed through two glass vessels containing either an odor source or control (hexane) stimuli and entered each of the branches of the Y-tube. An air supply system (Power Air Pump, Seoul, Korea) was used for air filtration and flow rate control. Before and after the bioassay, the Y-tube was cleaned with 100% methanol. Test thrips were used three days after adult emergence. Each test used 10 ~ 30 individuals of each sex and was replicated four times by changing the source and control for replication.”

**Comment #3-7**: Line 189-190 discussing the sampling of flowers states: “in flowers in 10 randomly selected flowers was counted in three fields three times a week for 190 one month (June 13  July 13) once in a week”. This statement is confusing, was the sampling conducted three times a week or one time per week?

**Response**: This is rephrased as follows: “Number of F. occidentalis in flowers was counted from 10 randomly selected flowers with three replications in each field. The monitoring was performed once a week for one month (June 13  July 13).”

**Comment #3-8**: Line 222- should be arcsine perhaps?

**Response**: Corrected as suggested

**Comment #3-9**: In discussing the results of the field study of the push and pull components, it is stated that the Frankliniella occidentalis numbers are lower in the experimental greenhouse, but what were the results for each life stage and sex? Was it only the total number of all life stages and males and females that were reduced, or was there a difference in the response of the different life stages (larvae versus adults) or sexes?

**Response**: We did not examine larval density because we assessed the efficacy mostly based on the trapping adults on the yellow sticky trap. However, the figure 6C suggests the total number of larvae and adults. Thus the reduction by the push-pull can be explained by the total reduction not only by adults.
