# Supplementary material for: A push-pull strategy to control the western flower thrips, Frankliniella occidentalis, using alarm and aggregation pheromones
Source: PLoS One. 2023 Feb 24;18(2):e0279646. doi: 10.1371/journal.pone.0279646 (PMC9956899; doi:10.1371/journal.pone.0279646)
Supplement: Attachment — Submitted filename: Response to Reviewers comments.docx [file pone.0279646.s006.docx]

**Reviewers’ comments**

**[Reviewer #1]**

**Comment**: All corrections were done. I feel that this manuscript A push-pull strategy to control the western flower thrips, Frankliniella occidentalis,using alarm and aggregation pheromones is now acceptable for publication

**Response**: Thank you for a nice review!

**[Reviewer #2]**

**Comment**: I read carefully the revised text of your mns PONE -D-22-23616, titled " A push-pull strategy to control the Western Flower Thrips, Frankliniella occidentalis,using alarm and aggregation pheromones". Even I read the replies you provided to each reviewers. I appreciated the effort done in order to justify and preserve your previous "draft", or to accept and change some parts as suggested by reviewers. In particular, I am satisfied for the adding of several new data to minor/major issues recorded in M& M and Results Sections. Such as detailed records regarding some texts , i.e. in subprgrs. 2.4, 2.5, 2.9 that more improved the data presentation , technical clarification , i.e. 2.3, and statistical analysis , i.e. 2.11. Details were added also to Results Section , i.e. F and P values in 3.1, 3.2 and 3.4 subparagraphs. The new version of Discussion Section has more clearly recorded the main steps obtained in the technical part of this study, such as the control efficacy of push-pull tactics against Frankliniella occidentalis. Also the actractiveness of the aggregation pheromone compounds has been critically outlined for the other thrips species considered. Finally, the future perspectives of the novel push-pull tactics in the control of pest thrips might be proposed for open field applications. The well presented comments let me satisfied of the new version of the text, even without a separated Conclusions Section. Here, I attached the pdf of the revised version of the text that I consider suitable for publication.

**Response**: Thank you for a nice review!

**[Reviewer #3]**

**Comment**: Section 3-3 in the results section still reports statistical values (t-test and chi-squared) that are not mentioned in the methods section for statistical analyses performed. Are these the post-hoc analyses, or were they just forgotten in the methods section?

**Response**: The stat method is now added to the M&M.

**Comment**: At the end of section 3-3 there is an F-value reported of 1600.00. This is an exceptionally high F-value, is that the accurate F-value or is the decimal point in the wrong place, for example should it actually be 160 or 16? If it is indeed 1600, that is very impressive!

**Response**: We calculated the F value again and confirmed

**Comment**: All other comments have been addressed, the manuscript looks great.

**Response**: Thank you for a nice review!
